# Thermal Conductivity and Viscosity: Review and Optimization of Effects of Nanoparticles

**DOI:** 10.3390/ma14051291

**Published:** 2021-03-08

**Authors:** Kevin Apmann, Ryan Fulmer, Alberto Soto, Saeid Vafaei

**Affiliations:** Mechanical Engineering Department, Bradley University, Peoria, IL 61606, USA; kapmann@mail.bradley.edu (K.A.); rfulmer@mail.bradley.edu (R.F.); asoto@mail.bradley.edu (A.S.)

**Keywords:** thermal conductivity, viscosity, nanoparticles, optimization of effects of nanoparticles

## Abstract

This review was focused on expressing the effects of base liquid, temperature, possible surfactant, concentration and characteristics of nanoparticles including size, shape and material on thermal conductivity and viscosity of nanofluids. An increase in nanoparticle concentration can lead to an increase in thermal conductivity and viscosity and an increase in nanoparticle size, can increase or decrease thermal conductivity, while an increase in nanoparticle size decreases the viscosity of the nanofluid. The addition of surfactants at low concentrations can increase thermal conductivity, but at high concentrations, surfactants help to reduce thermal conductivity of the nanofluid. The addition of surfactants can decrease the nanofluid viscosity. Increasing the temperature, increased the thermal conductivity of a nanofluid, while decreasing its viscosity. Additionally, the effects of material of nanoparticles on the thermal conductivity and viscosity of a nanofluid need further investigations. In the case of hybrid nanofluids, it was observed that nanofluids with two different particles have the same trend of behavior as nanofluids with single particles in the regard to changes in temperature and concentration. Additionally, the level of accuracy of existing theoretical models for thermal conductivity and viscosity of nanofluids was examined.

## 1. Introduction

Currently, working fluids are used throughout the world through many different applications. These working fluids can consist of water, ethylene glycol, and various oils. They are used in many industries which include, but are not limited to: power generation, aerospace, medical field, and transportation. However, these fluids have a strong limiting factor when it comes to their ability to transfer heat. This is why there is a lot of research being done to try and improve this limiting factor. One of the main ways these working fluids are being modified is by the addition of nanoparticles to create what are called nanofluids. A nanofluid is a mixture of nanoparticles within a base fluid. Nanoparticles change the physical properties of the working fluids including the thermal conductivity and viscosity. Initially, attempts were made to increase the thermal properties of working fluids with the millimeter and micrometer sized particles. These fluids had many problems though such as clogging of fluid paths, abrasion, and pressure drop [1]. In an effort to create a working fluid without these problems in 1995, Choi [2] first added nanoparticles to working fluids to create a nanofluid. A significant increase in the thermal conductivity of the fluids was observed. Most metals almost always have a higher thermal conductivity than liquids, therefore introducing a metal to a working fluid improves the ability of that fluid to transfer heat.

A significant amount of research has been done to identify the specific parameters that determine the thermal conductivity and viscosity of a nanofluid. Such parameters include the concentration of nanoparticles, size of nanoparticles, surfactants, temperature, base fluid, shape of nanoparticle, and using a hybrid nanofluid. Less research has been done examining the effect of these parameters on viscosity, but viscosity is an important concern in the design of nanofluids. The resistance of a fluid to flow and random motion of particles and molecules is directly tied to the viscosity of that fluid [2]. Therefore, the heat transfer and pumping power required to pump a nanofluid could be greatly influenced by the characteristics of the nanofluid. This could have serious implications on the design of devices that utilize nanofluids.

This paper investigates the effects of concentration and characteristics of nanofluids on nanofluid viscosity and thermal conductivity. For the experimental work reviewed in this paper many of the nanofluids were created by the addition of commercially obtained nanoparticles into the base fluid and possible surfactant. Several experimental works relied on nanoparticles that were synthesized by the researchers for the creation of nanofluids. The procedures used to synthesize the nanoparticles will be discussed with the applicable papers. It will also explain how to optimize the effects of nanoparticles on thermal conductivity and viscosity of nanofluids, while also analyzing the level of accuracy of current theoretical models that exist. This will allow nanofluids to be designed in a manner that will maximize the thermal conductivity to allow for the most effective heat transfer, while simultaneously minimizing the viscosity to reduce pumping power.

## 2. Results and Discussion

### 2.1. Effects of Concentration of Nanoparticles

**Figure 1:**
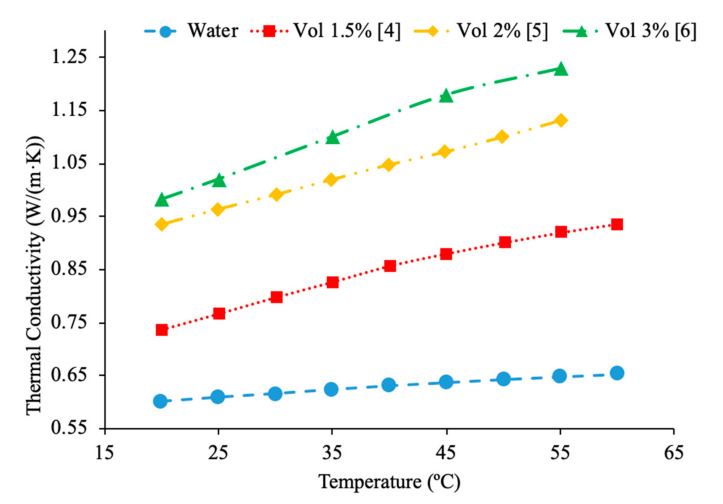
Thermal conductivity of Fe_3_O_4_–water nanofluids at 1.5%, 2%, and 3% volume fractions as a function of temperature [4,5,6].

Nanoparticle concentration has a significant impact when it comes to the relation to nanofluid thermal conductivity and viscosity. It was observed that as the concentration of nanoparticles increases thermal conductivity increases. Yeganeh et al. [3] measured the effect of concentration on the thermal conductivity of nanodiamond (ND)–water nanofluids. The thermal conductivity measurements were made using a KD2-pro instrument, which uses the transient line heat source method to measure thermal conductivity. Measurements were taken on volume fraction ranging from 0.8% to 3% and at temperatures of 30 to 50 °C. Across the range of temperatures tested, as the concentration increased, the thermal conductivity also increased. Additionally, the relationship between relative thermal conductivity and volume fraction was nonlinear. In addition, Sundar et al. [4] measured the thermal conductivity of Fe_3_O_4_–water nanofluids as a function of nanoparticle concentration by using a transient hotwire system. These nanoparticles were synthesized by using ferric chloride, ferrous chloride, and sodium hydroxide. Details can be seen in referenced paper. Volume fractions ranging from 0.2% to 2% were measured and an increase in thermal conductivity was observed as the volume fraction increased. This increase in thermal conductivity could be caused by an increase in Brownian motion of the nanoparticles at higher concentrations. Brownian motion creates microconvection in the surrounding liquid molecules, increasing thermal conductivity. Gao et al. [5] measured the thermal conductivity of Fe_3_O_4_–water nanofluids at volume fraction of 0.05%, 0.5%, and 2% using the transient hotwire method across a range of temperatures from 10 to 55 °C. The nanofluids were prepared with the co-precipitation method. Details can be seen in referenced paper. There was an increase in thermal conductivity with the increase in nanoparticle concentration. The increase in thermal conductivity is thought to be caused by an increase in Brownian motion, the formation of a nanolayer on the particles, and convection from the particle motion. Afrand et al. [6] measured the thermal conductivity of Fe_3_O_4_–water nanofluids at volume fractions ranging from 0.1% to 3%. The measurements were made across a temperature range of 20 to 55 °C using the transient hotwire method. It was observed that there was an increase in thermal conductivity as the concentration increased, which can be caused by an increase in Brownian motion. Brownian motion creates more interaction between the particles, leading to higher thermal conductivity. In Figure 1, the thermal conductivity of Fe_3_O_4_–water nanofluids are shown. From Figure 1, it can clearly be seen that the thermal conductivity increases as the volume fraction increases, but the largest increase occurs at lower concentrations. At higher concentrations, the thermal conductivity increases at a lower rate. Using the transient hotwire method, Godson et al. [7] measured the thermal conductivity of Ag–water nanofluids. The measurements were made on nanofluids with volume fractions of 0.3%, 0.6%, and 0.9%. It was shown that the thermal conductivity of the nanofluids increased with the increase in volume fraction. As the volume fraction increased, the velocity of the Brownian Motion of the particles and the thermophoresis of the nanoparticles increased. Thermophoresis is the motion of particles caused by a temperature gradient in the fluid. Sometimes, thermophoresis could have a larger effect than Brownian motion, however, both factors contribute to increased particle collision. Most of the time, these particle collisions would cause the increase in thermal conductivity. Li et al. [8] measured the thermal conductivity of both Al_2_O_3_–water and CuO–water nanofluids as a function of the volume fraction. The nanofluids tested had volume fractions of 2%, 6%, and 10% for the Al_2_O_3_–water nanofluids and volume fractions of 2%, 4%, and 6% for the CuO–water nanofluids. Both the Al_2_O_3_ and CuO nanofluids showed an increase in thermal conductivity as the volume fraction increased. The increase in thermal conductivity at higher concentrations can be explained by an increase in possible collision of particles and Brownian motion at higher concentrations. Brownian motion could cause stirring of the water molecules, which created microconvection within the liquid increasing heat transfer.

Additionally, Pryazhnikov et al. [9] measured the thermal conductivity of Al_2_O_3_–water and TiO_2_–water nanofluids as a function of volume fraction. The measurements were made using the nonstationary hotwire method on nanofluids with volume fractions ranging from 1% to 6%. Both nanofluids measured demonstrated an increase in thermal conductivity as the volume fraction increased. Xie et al. [10] measured the thermal conductivity of MgO-ethylene glycol (EG) nanofluids as a function of concentration. The measurements were made using the transient hotwire method on nanofluids with volume fractions ranging from 0.5% to 5% across a range of temperatures 10 to 60 °C. As the concentration of nanoparticles increased, the thermal conductivity increased. The highest thermal conductivity increase was measured in the nanofluid with a volume fraction of 5%, which had an increase in thermal conductivity of 40.6% at 30 °C. One may suggest that the increase in thermal conductivity with increased concentration was caused by an increase in particle aggregation which could happen. Particle aggregation could place the particles in a higher contact area with one another allowing for higher thermal conductivity. Yu et al. [11] measured the thermal conductivity of ZnO-EG nanofluids across a temperature range of 10 to 60 °C at various volume fractions. The thermal conductivity increased non-linearly as the concentration increased. Additionally, at a volume fraction of 5%, there was a 26.5% increase in thermal conductivity compared to the base fluid. Figure 1 shows the variation of thermal conductivity as a function of temperature for different particle volume fractions. This figure demonstrates that as temperature and particle volume increases, nanofluid thermal conductivity increases.

**Figure 2:**
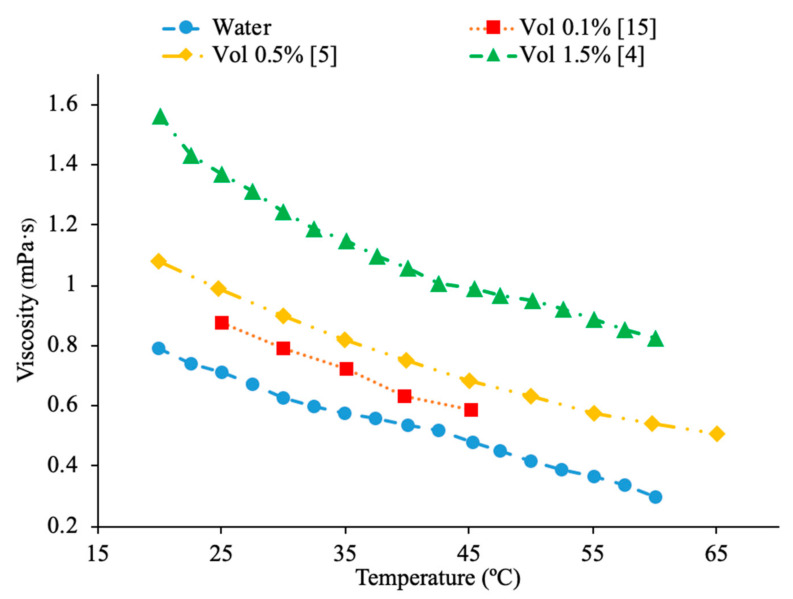
Viscosity of Fe_3_O_4_–water nanofluids at 0.1%, 0.5%, and 1.5% volume fractions as a function of temperature [4,5,15].

In addition, there have been studies concluding that as the concentration of nanoparticles increases, there is an increase in the viscosity of the nanofluid. Nguyen et al. [12] studied the effect of nanoparticle concentration on the viscosities of Al_2_O_3_–water and CuO–water nanofluids. The viscosity was measured using a ‘piston-type’ calibrated viscometer. The particle diameters of these fluids were 36 and 47 nm for water–Al_2_O_3_ and 29 nm for water–CuO. These fluids were exposed to room temperature of roughly 25 °C. The particle volume concentrations varied from 0.15% to 13%. As the particle volume concentration increased, so did the nanofluid viscosity. The 47 nm water–Al_2_O_3_ relative viscosity increased from 1.12 to 1.6, then to 3.0, and then to 5.2 for particle volume concentrations of 1%, 4%, 9%, and 12% respectively. Furthermore, Yiamsawas et al. [13] also measured the effect of nanoparticle concentration on the viscosity of nanofluids. TiO_2_–water nanofluids at volume fractions from 1% to 8% and Al_2_O_3_–water nanofluids at a volume fraction from 1% to 4% were measured using a Cannon Instrument capillary tube viscometer across a temperature range of 15 to 60 °C. The viscosity of the TiO_2_–water nanofluid increased as the volume fraction of nanoparticles increased. The Al_2_O_3_–water nanofluid also showed an increase in viscosity as the volume fraction increased. It was observed that the increase in viscosity caused by an increase in concentration of nanoparticles was less at high temperatures. That could happen because of the greater distance between the molecules of the base fluid at these high temperatures. Kole et al. [14] performed viscosity measurements on Al_2_O_3_ nanofluids with engine coolant as a base fluid. The engine coolant was a mix of 50% propylene glycol and 50% water, and the viscosity measurements were made using an LDVD-II-Pro Brookfield programmable viscometer. The Al_2_O_3_-engine coolant nanofluids were measured at volume fractions ranging from 0.1% to 1.5% and the viscosity increased with an increase in volume fraction. These results showed a nonlinear relationship between volume fraction and relative viscosity. Sundar et al. [4] also measured the viscosity of Fe_3_O_4_–water nanofluids as a function of the particle concentration. The measurements were made using TA Instruments AR-1000 Rheometer and Fe_3_O_4_–water nanofluids with volume fractions ranging from 0.2% to 2% were tested. The experiments showed an increase in the viscosity as the volume fraction increased. The nanofluids tested were also found to behave as Newtonian fluids. The increase in viscosity with the increase in volume fraction can be caused by an increased interaction between particles. Gao et al. [5] measured the viscosity of Fe_3_O_4_–water nanofluids at a volume fraction from 0.05% to 2% across a temperature range of 10 to 65 °C. The measurements were made using a Brookfield viscometer and as the concentration increased the viscosity of the nanofluids increased. Malekzadeh et al. [15] measured the viscosity of Fe_3_O_4_–water nanofluids at volume fractions from 0.1% to 1% using a Brookfield viscometer across a temperature range from 25 to 45 °C. It was observed that there is an increase in viscosity with an increase in concentration due to the increased molecular interaction between the nanoparticles and base liquid at higher concentrations. In Figure 2, the viscosity of Fe_3_O_4_–water nanofluids are shown. Figure 2 shows the variation of viscosity as a function of temperature for different nanoparticle volume fractions. This figure shows that as temperature increases, viscosity decreases. On the other hand, it also shows that as nanoparticle volume concentration increases, viscosity increases. Godson et al. [7] measured the viscosity of Ag–water nanofluids as a function of particle concentration using a reverse flow viscometer. The Ag–water nanofluids measure had volume fractions of 0.3%, 0.6%, and 0.9%, and the viscosity was observed to increase with an increase in the volume fraction. Yu et al. [16] measured the effect of concentration on the viscosity of AlN-ethylene glycol (EG) and AlN-propylene glycol (PG) nanofluids. The measurements were made using an LDVD-II-Pro Brookfield programmable viscometer on nanofluids at 20 °C with volume fractions from 5% to 9%. For both nanofluids tested, as the volume fraction increased, the viscosity also increased.

**Figure 3:**
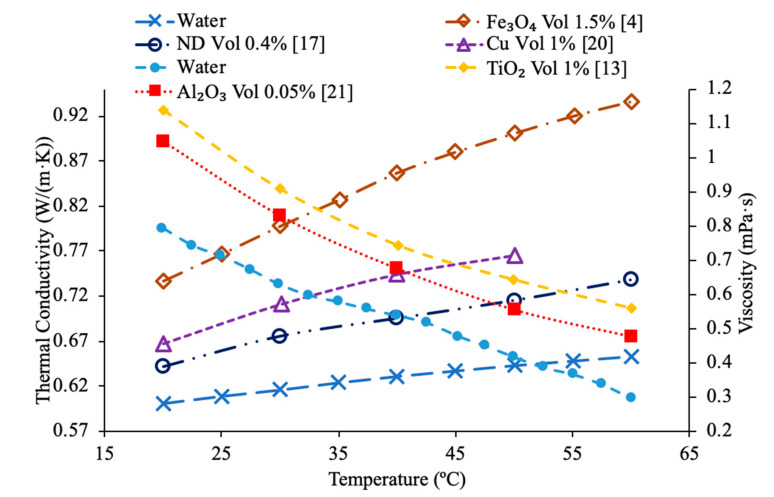
Thermal conductivity (increasing with temperature) and viscosity (decreasing with temperature) for various water-based nanofluids at various volume fractions [4,13,17,20,21].

**Figure 4:**
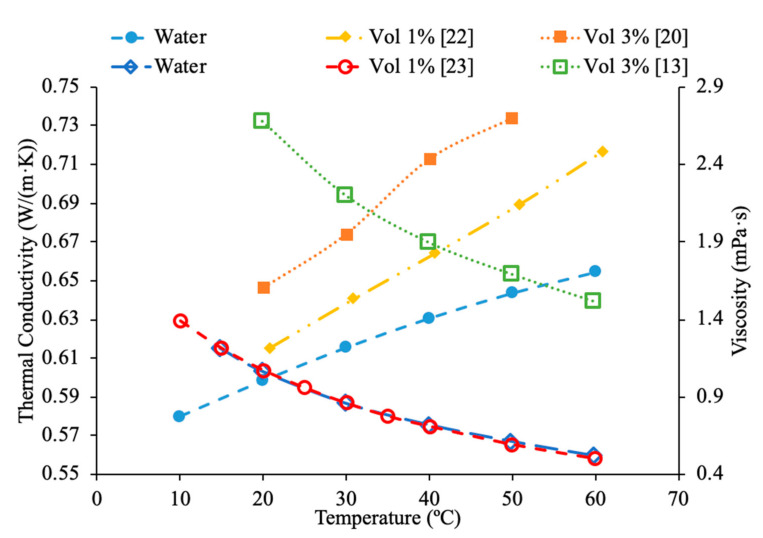
Thermal conductivity (increasing with temperature) and viscosity (decreasing with temperature) for Al_2_O_3_–water nanofluids at a volume fraction of 1% and 3% [13,20,22,23].

Sundar et al. [17] measured the thermal conductivity and viscosity of ND–water nanofluids. Sulfuric acid and nitric acid were used to remove carbon impurities from the nanodiamond prior to them being used to produce nanofluids. Details can be seen in referenced paper. The thermal conductivity measurements were made using transient hotwire method and the viscosity measurements were made using an A&D vibro viscometer. The nanofluids tested had concentrations from 0.2% to 1% and measurements were taken from 20 to 60 °C. Across the range of temperatures tested, as the concentration increased, the thermal conductivity and viscosity both increased. The largest enhancement in thermal conductivity was seen in the nanofluid with a volume fraction of 1% at 60 °C, which had a thermal conductivity 22.86% higher than the base fluid. At a volume fraction of 1% and a temperature of 60 °C, the viscosity was 1.79 times greater than the base fluid, which was the largest increase of any of the measurements taken. It can be seen that the thermal conductivity increases as the concentration increases when the data from various studies are compared, and the viscosity is shown to increase as the concentration increases. Pastoriza-Gallego et al. [18] measured the thermal conductivity and viscosity of Al_2_O_3_–ethylene glycol nanofluids as a function of volume fraction. The measurements were made using the transient hotwire method for thermal conductivity and a Schott rotational viscometer for viscosity. Volume fractions ranging from 1.5% to 8.6% were used for the thermal conductivity measurements while volume fractions from 0.5% to 6.6% were used for the viscosity measurements. The measurements reveal an increase in thermal conductivity and viscosity as the volume fraction increases. At 50 °C, the thermal conductivity of the nanofluid at a volume fraction of 8.6% had a thermal conductivity 18.4% higher than the base fluid, while the nanofluid at a volume fraction of 1.5% only had a 2.7% increase. At 50 °C, the viscosity of the nanofluid with a volume fraction of 6.6% had a viscosity 2.1 times the base fluid while the nanofluid with a volume fraction of 2.1% had a viscosity 1.3 times greater than the base fluid. Sundar et al. [19] measured the thermal conductivity and viscosity of nanofluids made using Al_2_O_3_ nanoparticles and a base liquid made of water and ethylene glycol. The thermal conductivity measurements were made using a KD2 Pro Thermal Analyzer and the viscosity measurements were made using an AR-1000 Rheometer. The nanofluids tested had volume fractions between 0.3% and 1.5% and the measurements were taken at temperatures between 20 and 60 °C. As the concentration of the nanofluid increased, the thermal conductivity and viscosity both increased. In Figure 3, the viscosity and thermal conductivity are shown for various water-based nanofluids. It can be concluded from Figure 3, that even though different materials of nanoparticles are used, the increase in concentration of nanoparticles increases the thermal conductivity and viscosity of the nanofluids. The highest thermal conductivity is seen in the Fe_3_O_4_–water nanofluid with a volume fraction of 1.5%, and the highest viscosity is seen in the TiO_2_–water nanofluid with a volume fraction of 1%. Figure 4 shows the thermal conductivity and viscosity of water-based Al_2_O_3_ nanofluid at volume concentrations of 1% and 3%. When looking at the figure, the Al_2_O_3_ water-based nanofluid at 4% volume concentration has the highest thermal conductivity and viscosity following the Al_2_O_3_ water-based nanofluid at the 3%, 2%, and then 1% having the lowest thermal conductivity and viscosity. Therefore, it is concluded that as the concentration of the nanoparticles within a nanofluid increases the thermal conductivity and viscosity increases.

### 2.2. Effects of Nanoparticle’s Size

**Figure 5:**
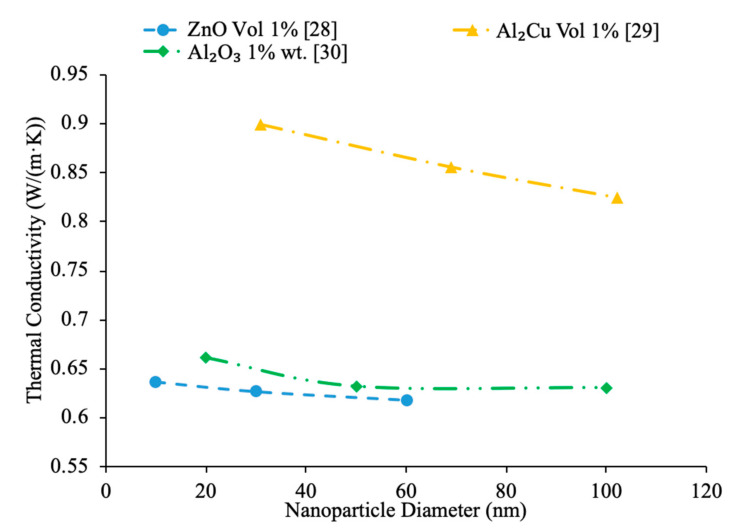
Thermal conductivity of water-based SiO_2_, Al_2_O_3_, and TiO_2_ nanofluids at 1% volume fraction as a function of nanoparticle diameter [28,29,30].

Furthermore, the size of nanoparticles has a great effect on nanofluid thermal conductivity and viscosity. As nanoparticle size increases, thermal conductivity of the nanofluid decreases, and other times as nanoparticle size increases, thermal conductivity increases. Kwek et al. [24] performed measurements on the thermal conductivity on Al_2_O_3_–water nanofluids. The nanofluids had a volume fraction of 5% and nanoparticles with diameters 10, 25, 35, 80 and 150 nm. The transient hotwire method was used to measure the thermal conductivity of the nanofluids. The data collected showed an initial decline in thermal conductivity as the size of the particle diameter begins to increase once the particle diameter reaches 35 nm the thermal conductivity begins to increase as the nanoparticle size increases. The initial decline in thermal conductivity is thought to be caused by a decrease in Brownian motion as the size of the particles increase. Smaller particles can move faster and have a greater Brownian motion, which creates more convection in the base fluid. Additionally, nanoparticles can carry energy as results of diffusive heat transfer, which depends on many factors including size and speed of nanoparticles. Diffusive heat transfer allows the heat absorbed by the particles to be carried to other locations throughout the base liquid. Beck et al. [25] also measured how the diameter of Al_2_O_3_ nanoparticles affect the thermal conductivity of Al_2_O_3_–water nanofluids using nanofluids containing particles ranging from 8 to 282 nm in diameter. The thermal conductivity was measured using the liquid metal transient hot wire device. The measurements were made on nanofluids with a 4% volume fraction at room temperature. The thermal conductivity of the nanofluid was found to decrease as the size of the nanoparticles decreased below around 50 nm in size. This was due to an increase in phonon scattering in the smaller particles. Rudyak et al. [26] measured the thermal conductivity of a variety of water-based nanofluids using different materials and diameters of nanoparticles. Measurements were taken using the hotwire method and using nanoparticles ranging from 10 to 150 nm in SiO_2_–water, Al_2_O_3_–water, and TiO_2_–water nanofluids at 2% volume fraction. All the nanofluids tested showed an increase in relative thermal conductivity as the size of the nanoparticles increased. It is thought that the ratio of the diameter of the nanoparticle to the diameter of the base fluid is a parameter in the thermal conductivity of nanofluids [27]. Chon et al. [22] measured the thermal conductivity of Al_2_O_3_–water nanofluids with nanoparticle diameters of 150, 47 and 11 nm. Increasing the particle size reduced the thermal conductivity. The decrease in thermal conductivity with the increase in particle size can be explained by the effect of particle size on Brownian velocity. Larger particles have a lower Brownian motion, meaning there is less movement of the nanoparticles in the base fluid. The motion of the particles in the base fluid allows for the particles to transfer energy, so reducing the motion will reduce energy transfer. In Figure 5, the thermal conductivity as a function of particle size is shown, demonstrating the observed decrease in thermal conductivity with increase in particle size (increase in diameter of nanoparticles). In nanofluids made Al_2_Cu nanoparticles, the decrease in thermal conductivity as nanoparticle size increases is the greatest. Whereas the thermal conductivity decreased at a very small rate for the Al_2_O_3_ and ZnO nanofluids in terms of increasing particle size.

**Figure 6:**
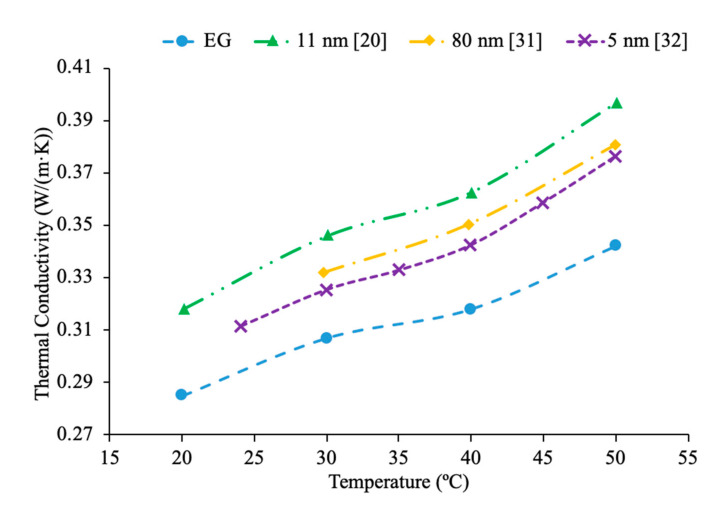
Thermal conductivity of Al_2_O_3_–ethylene glycol (EG) nanofluids as a function of temperature at a volume fraction of 1% with nanoparticle diameters of 5, 11 and 80 nm [20,31,32].

**Figure 7:**
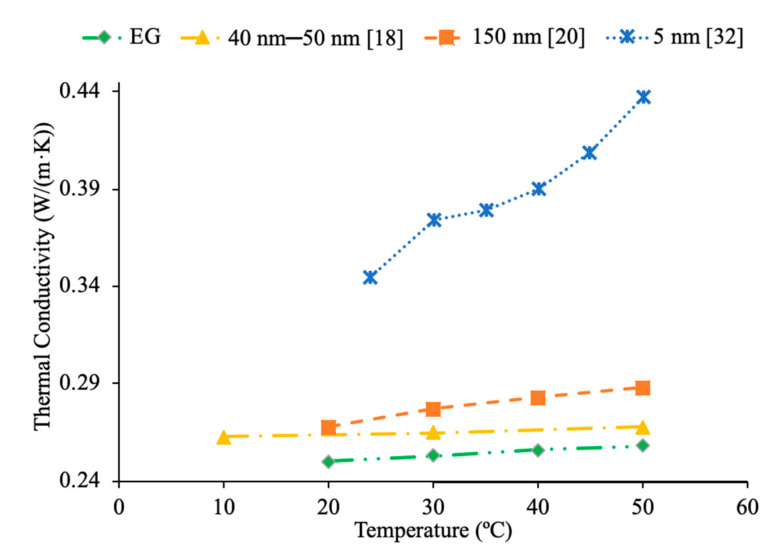
Al_2_O_3_–ethylene glycol (EG) nanofluids at 3% volume fraction with nanoparticle diameters of 5, 150 and 40–50 nm as a function of temperature [18,20,32].

Patel et al. [20] measured the thermal conductivity of Al_2_O_3_–ethylene glycol nanofluids at a particle fraction of 3% and particle diameters of 11 and 150 nm. Measurements were also made using Al_2_O_3_–EG nanofluids at 1% volume fraction and 11 and 45 nm particles. These measurements showed an increase in thermal conductivity as the particle size decreased, which can be caused by an increase in Brownian motion and an increase in the surface area to volume ratio for the smaller nanoparticles. Moreover, greater Brownian motion allows for more paths for heat transfer. Additionally, a greater surface area to volume ratio increases thermal conductivity since heat transfer is a function of surface area. Murshed et al. [31] measured the thermal conductivity of Al_2_O_3_–EG nanofluids at 1% volume fraction and with nanoparticles with a diameter of 80 nm. The thermal conductivity increased with nanoparticle volume fraction increasing. Esfe et al. [32] studied the thermal conductivity of Al_2_O_3_ nanofluids with ethylene glycol (EG) as a base liquid with varying concentrations. In particular, he studied the thermal conductivity of these nanofluids with 5 nm nanoparticles over a range of concentrations of 0.2% to 5% and a range of temperatures of 24 to 50 °C using KD2-Pro thermal analyzer. The thermal conductivity of the nanofluids increased with nanoparticle concentration. In Figure 6, it can be seen that the highest thermal conductivity was measured for the nanofluid containing the 11 nm particles followed by the 80 nm particles and then the 5 nm particles. This suggests that sometimes the smallest nanoparticles provide the highest thermal conductivity, but this does not always hold true. Pastoriza-Gallego et al. [18] also measured the thermal conductivity of Al_2_O_3_–EG nanofluids, and the nanoparticles used in the measurements were between 40 and 50 nm. Figure 7 explains that the thermal conductivity of the nanofluid with nanoparticles with a diameter of 40–50 nm is lower than the nanofluids containing 5 and 150 nm diameter nanoparticles. This agrees with what has been seen in other research, whereas nanoparticle diameter increases, there is an initial decrease in thermal conductivity followed by an increase, but the smallest particles still have the highest thermal conductivity.

**Figure 8:**
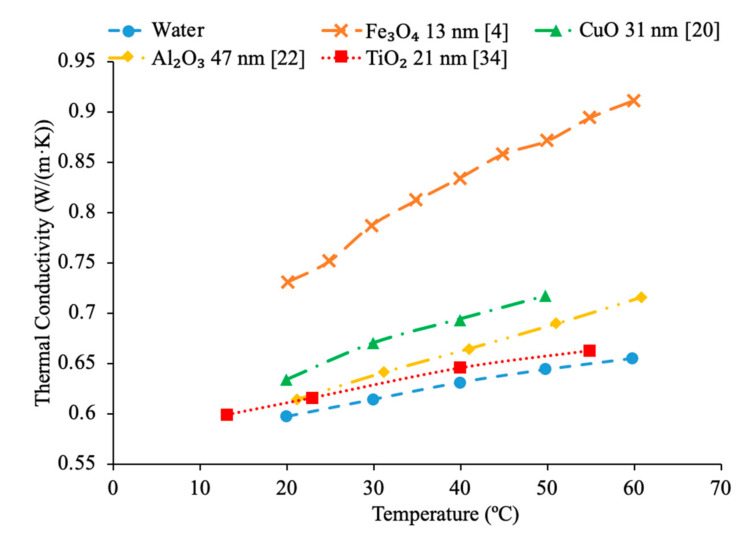
Thermal conductivity of water-based nanofluids of CuO, Fe_3_O_4_, Al_2_O_3_, and TiO_2_ as a function of temperature with various sized nanoparticles of 13, 21, 31 and 47 nm [4,20,22,34].

Kim et al. [28] also looked at how the variation in nanoparticle size affects the thermal conductivity of a nanofluid. The transient hotwire technique was used to measure the thermal conductivity of ZnO-water and TiO_2_–water nanofluids. Unlike the other experiments that were looked at, as the size of the nanoparticles increased, the thermal conductivity consistently decreased. The decrease in thermal conductivity was not very large suggesting that the change in size of the particle may not have had a significant effect. Omrani et al. [33] measured the thermal conductivity of nanofluids made with various sized multiwalled carbon nanotubes (MWCNT) and water. The nanofluids had a volume fraction of 0.05% and the measurements were taken across a range of temperatures from 10 to 45 °C using the transient hotwire method. All of the nanofluids tested increased in thermal conductivity as the temperature increased, but the largest increase was seen in nanofluids containing MWCNTs with a diameter of just above 8 nm and a length of 10–30 μm. The smallest increase in thermal conductivity with temperature was seen in the nanofluid made with nanoparticles with a diameter of just under 50 nm and a length of 0.5–2 μm. The thermal conductivity is thought to be related to the aspect ratio of the MWCNT, as the highest increase in thermal conductivity was measured in the nanofluid with the highest aspect ratio nanoparticles, while the lowest increase was seen in the nanofluid made with the smallest aspect ratio MWCNTs. Higher aspect ratio leads to more agglomeration, which may allow for more heat transfer between particles. Figure 8 gives the thermal conductivity versus temperature for various nanofluids at different sizes of 13, 21, 31 and 47 nm. As can be seen by the figure, the Fe_3_O_4_ with 13 nm particles has the highest thermal conductivity across the range of temperatures following CuO with 31 nm particles, Al_2_O_3_ with 47 nm particles and TiO_2_ with 21 nm particles. This demonstrates that both the size and material affect the thermal conductivity of the nanofluid. While the Fe_3_O_4_, CuO, and Al_2_O_3_ nanofluids follow the trend whereas the particle size increases the thermal conductivity decreases, the TiO_2_ nanofluid is an exception being at a small size and a low thermal conductivity. Therefore, if both the particle size and material are changed the effect it has on thermal conductivity cannot necessarily be predicted. In general, it was observed that as the size of the nanoparticles increases, the thermal conductivity of the nanofluid can either increase or decrease. However, most experimental results showed that smaller nanoparticles within a nanofluid have a higher thermal conductivity than bigger nanoparticles. The nanoparticle size can affect the Brownian motion, nanoparticle-nanoparticle interactions, and energy transport from one point to another.

**Figure 9:**
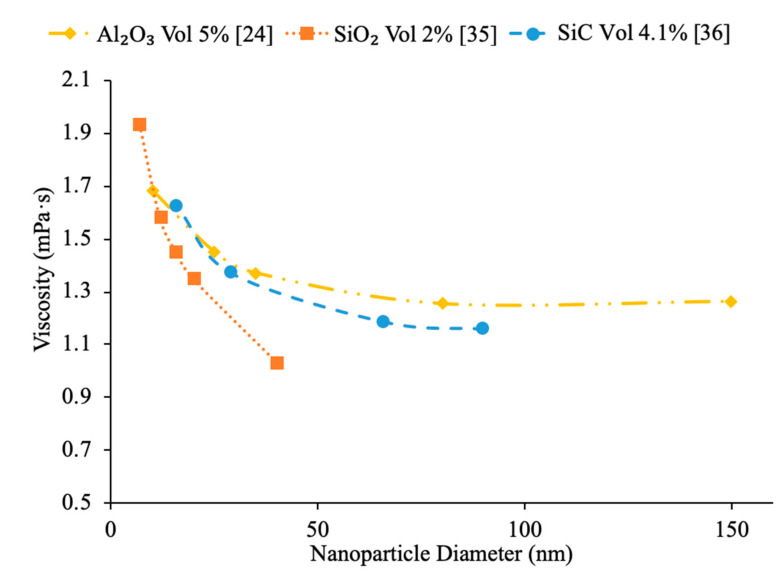
Viscosity of SiO_2_, Al_2_O_3_, and TiO_2_ water-based nanofluids as a function of nanoparticle diameter [24,35,36].

**Figure 10:**
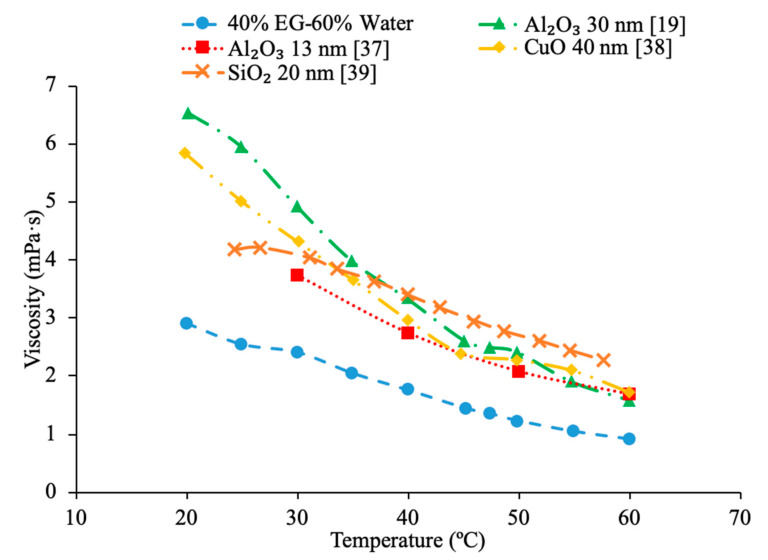
Viscosity of CuO, Al_2_O_3_, and SiO_2_ nanofluids with 40% ethylene glycol (EG) and 60% water as a base liquid as a function of temperature with various sized nanoparticles of 13, 20, 30 and 40 nm [19,37,38,39].

**Figure 11:**
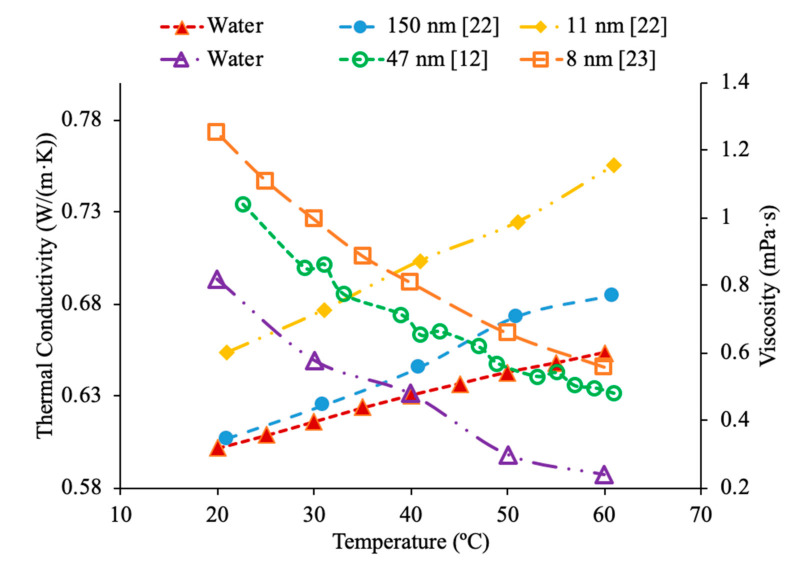
Thermal conductivity (increasing with temperature) and viscosity (decreasing with temperature) of various water-based nanofluids at 1% volume fraction and different sized nanoparticles [12,22,23].

Additionally, there have been studies to determine the effect of nanoparticle size to the viscosity of the nanofluid and researchers have found that there is a decrease in viscosity with an increase in nanoparticle size. Jia-Fei et al. [35] looked at how the size of nanoparticles affect the viscosity of a nanofluid. Measurements were performed using SiO_2_–water nanofluids and the viscosity measurements were made using a Ubbelohde-type capillary viscometer. SiO_2_–water nanofluids with nanoparticle diameters of 7 nm, 12 nm, 16 nm, 20 nm, 40 nm were all tested at volume fractions of 0.1%, 0.2%, 0.4%, 0.8%, 1.2%, 1.6%, and 2%. At each concentration, the viscosity of the nanofluid decreases as the size of the nanoparticle increases. Kwek et al. [24] performed measurements on the viscosity of Al_2_O_3_–water nanofluids. The nanofluids had a 5% volume fraction of Al_2_O_3_ nanoparticles and nanoparticles with diameters 10, 25, 35, 80 and 150 nm. The viscosity measurements were made using a Contraves LS 40 standard controlled rheometer. It was observed that as the size of the nanoparticle increased, the viscosity of the nanofluid decreased, until the particle reached about 85 nm, then the viscosity approached a constant value. Sometimes, the decrease in viscosity as size of nanoparticles increases can be caused by the fact that smaller nanoparticles tend to group together and form clustered nanoparticles more than larger nanoparticles do. Particle agglomeration is the formation of nanoparticles into a group, which leads to a higher viscosity for fluids containing these particles. Rudyak et al. [26] measured the viscosity of a variety of water-based nanofluids using different materials and diameters of nanoparticles. The measurements were taken using Brookfield viscometers and using nanoparticles ranging from 10 to 150 nm. SiO_2_–water, Al_2_O_3_–water, and TiO_2_–water nanofluids at 2% volume fraction all showed a decrease in viscosity as the diameter of the nanoparticle increased. Figure 9 shows viscosity versus nanoparticle diameter for various nanofluids. It can be seen that at each concentration the viscosity of each nanofluid decreases as particle diameter increases. Omrani et al. [33] measured the viscosity of nanofluids made with various sized multiwalled carbon nanotubes (MWCNT) and water. The nanofluids had a volume fraction of 0.05% and the measurements were taken across a range of temperatures from 10 to 45 °C using a Brookfield viscometer for viscosity. The viscosity of the nanofluids all decreased as the temperature increased. The difference in viscosity between the various nanofluids across the range of temperatures was very small, but the nanofluid with the highest viscosity had MWCNTs diameter of just under 50 nm and a length of 0.5–2 μm. The nanofluid made using MWCNTs with a diameter of just above 8 nm and a length of 10–30 μm had the lowest viscosity across the range of temperatures. The nanofluid with the lowest viscosity measured was the nanofluid with the highest aspect ratio. The aspect ratio had a relatively small impact on viscosity though. In Figure 10, the viscosity as a function of temperature for various sized nanofluids are compared. It can be concluded when looking at the figure that the Al_2_O_3_ nanofluid with 30 nm sized nanoparticles has the highest viscosity from 20 to around 37 °C and then the SiO_2_ nanofluid with 20 nm sized nanoparticles has the highest viscosity. At 60 °C, the CuO and Al_2_O_3_ nanofluid viscosities are basically the same, while the SiO_2_ nanofluid is higher. From this, it can be seen that the material influences viscosity as well as size. Therefore, simply knowing the size is not enough to predict viscosity, but the material also needs to be known. The effects of nanoparticle size on thermal conductivity and viscosity are shown in Figure 5, Figure 6, Figure 7, Figure 8, Figure 9, Figure 10, Figure 11, respectively. The exact effects of nanoparticle size on thermal conductivity and viscosity was not found yet, for given parameters such base liquid, concentration and characteristics of nanoparticles. These parameters have a significant role on the effects of nanoparticle size on thermal conductivity and viscosity which have to be discovered in future work.

In addition, in Figure 11, the thermal conductivity and viscosity of water-based nanofluids with different sized nanoparticles at 1% volume concentration are given as a function of temperature. It can be seen that the highest thermal conductivity measured is for the nanofluid containing 8 nm Al_2_O_3_ nanoparticles, which are the smallest particles measured. The lowest viscosity was for the nanofluid made using 150 nm Al_2_O_3_ nanoparticles. This reflects what other research has shown, which is that generally the smallest particles will have the highest thermal conductivity. The lowest viscosity was found in the Al_2_O_3_ nanofluid made with the largest diameter of particles at 150 nm, which reflects that as particle size increases the viscosity decreases. Gangadevi et al. [21] measured the thermal conductivity and viscosity of Al_2_O_3_–water nanofluids with 50 nm nanoparticles. The measurements were made using a KD2 Pro thermal property analyzer for thermal conductivity and a Brookfield viscometer for viscosity. Okonkwo et al. [40] measured the thermal conductivity of Al_2_O_3_–water nanofluids with 29.2 nm particles. The measurements were also made using a KD2 Pro thermal property analyzer for thermal conductivity and a Brookfield viscometer for viscosity. When comparing the data from Gangadevi et al. [21] to Okonkwo et al. [40], the 29.2 nm nanoparticle size had a higher thermal conductivity and viscosity than the larger nanoparticle size of 50 nm.

### 2.3. Effects of Surfactants

**Figure 12:**
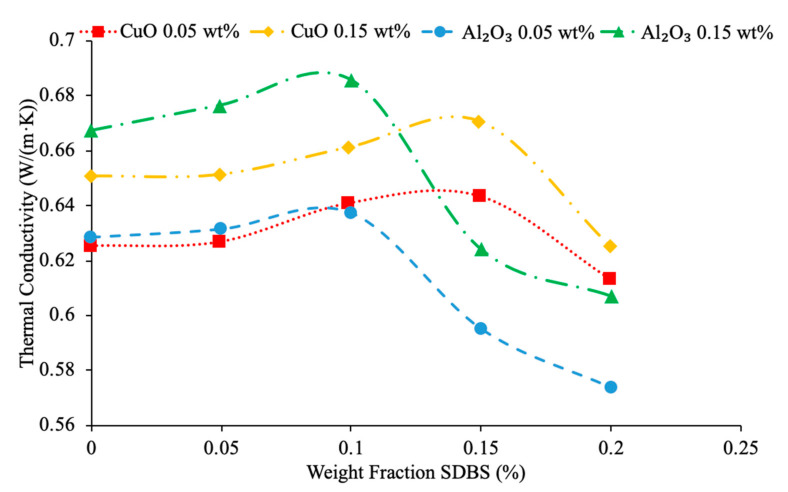
Thermal conductivity of water-based Al_2_O_3_ and CuO nanofluids as a function of weight fraction of sodium dodecyl benzene sulfonate (SDBS) surfactant [43].

Surfactants are used to create more stable nanofluids and prevent the nanoparticles from agglomeration and deposition. Instability in a nanofluid can have a negative effect on the thermal conductivity [41]. Experimental results have shown that in low concentrations, surfactants help increase thermal conductivity, but if the concentration of a surfactant is too high, it will reduce the thermal conductivity [42]. Khairul et al. [43] performed measurements on the thermal conductivity of CuO–water nanofluids and Al_2_O_3_–water nanofluids at various volume concentrations. Sodium dodecyl benzene sulfonate (SDBS) surfactant was used as well to stabilize the nanofluid. The SDBS negatively charges the surface of the nanoparticles creating electrostatic force that causes the nanoparticles to repel each other creating a more stable nanofluid. The measurements taken by Khairul et al. [43] using a KD2 Pro Thermal property analyzer to measure thermal conductivity of CuO–water and Al_2_O_3_–water nanofluids at weight percent of 0.05% and 0.15% are given as a function of surfactant concentration in Figure 12. When looking at Figure 12, it can be seen that the thermal conductivity of both the CuO–water nanofluids and Al_2_O_3_–water nanofluids initially increased with the addition of surfactant, but the thermal conductivity eventually reached a maximum value. The initial increase in thermal conductivity with the addition of surfactant can be caused by the fact that nanoparticles become less clustered and can move more freely in the base liquid. An additional layer of surfactant may deposit on nanoparticles by adding further surfactant into base liquid, which prevent heat transfer and energy transfer between nanoparticles. Therefore, a surfactant can increase thermal conductivity, but only in the right concentration.

Das et al. [44] performed measurements on the thermal conductivity of water-based nanofluids containing TiO_2_ nanoparticles and used cetyl trimethyl ammonium bromide (CTAB) and sodium dodecyl sulfate (SDS) as surfactants. The thermal conductivity measurements were taken across a range of temperatures from 20 to 60 °C using a KD2 Pro thermal analyzer, which utilizes the transient hotwire technique. The measurements of thermal conductivity were made using 0.1%, 0.5%, and 1% volume fractions of TiO_2_ nanoparticles in water-based nanofluids containing CTAB and SDS surfactants. At all concentrations and temperatures tested the thermal conductivity of the TiO_2_–SDS–water nanofluid was higher than the TiO_2_–CTAB–water nanofluid. Freitas et al. [45] measured the thermal conductivity of nanofluids using multiwalled carbon nanotubes (MWCNT) with water as a base fluid and several different surfactants used to improve the stability of the nanofluid. The surfactants used were Arabic gum (AG) at 0.25 weight percent, Triton’s X-100 (TrX) at 0.25 weight percent, and MWCNTs with a COOH acid group attached to them. The measurements of thermal conductivity were made using the hotwire method on nanofluids with a weight fraction of 0.5% and 1% across a range of temperatures from 30 to 60 °C. Each nanofluid was measured to increase in thermal conductivity with the increase in temperature and weight fraction. The highest thermal conductivity across the range of temperatures and at 1% weight fraction measured was the COOH–MWCNT–water nanofluid, followed by the MWCNT–AG–water, and MWCNT–TrX–water. In general, one can suggest that there is a certain concentration of surfactant that maximizes the thermal conductivity of a nanofluid that must be determined to optimize the heat transfer abilities of a nanofluid, and different surfactants will have different effects on the thermal conductivity of nanofluids.

**Figure 13:**
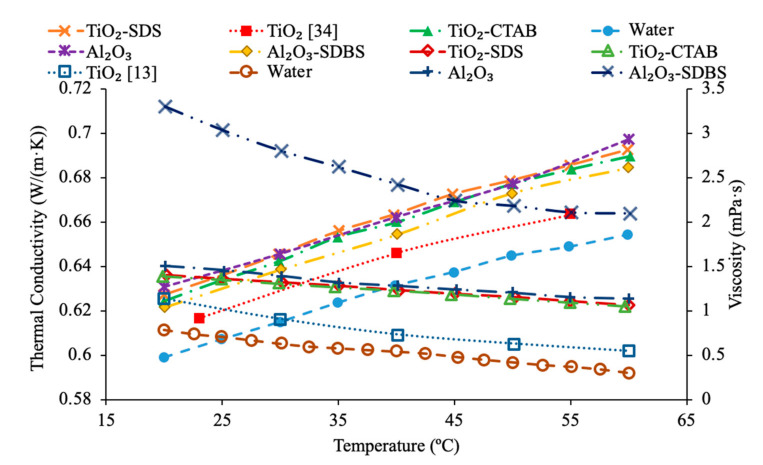
Thermal conductivity (increasing with temperature) and viscosity (decreasing with temperature) of TiO_2_–water nanofluids at 1% volume fraction with 0.39% SDS and 0.39% cetyl trimethyl ammonium bromide (CTAB) surfactants [44], TiO_2_ with no surfactant [13,34], and Al_2_O_3_–water nanofluids at 1% volume fraction with and without SDBS surfactant [46].

**Figure 14:**
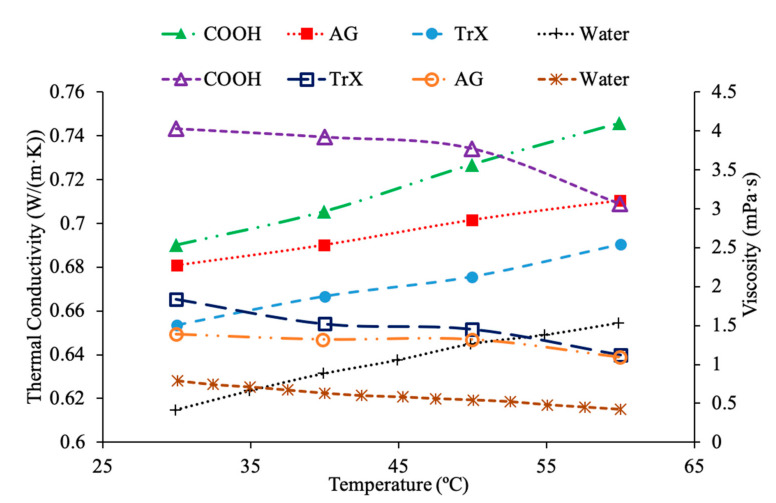
Thermal conductivity (increasing with temperature) and viscosity (decreasing with temperature) of multiwalled carbon nanotube (MWCNT)–water nanofluid at 1 wt %, with a COOH acid group, Triton’s X-100(TrX) at 0.25 weight percent, or Arabic Gum (AG) at 0.25 wt % surfactants shown using measurements [45].

Additionally, Khairul et al. [43] performed measurements on the viscosity of CuO–water nanofluids and Al_2_O_3_–water nanofluids with SDBS surfactant. The viscosity measurements were made using an AR-G2 rotational rheometer made by TA Instruments. As the concentration of surfactant increased, the viscosity of both CuO–water nanofluids and Al_2_O_3_–water nanofluids generally decreased, but there was some fluctuation making it difficult to establish a clear relationship between SDBS concentration and viscosity. The minimum viscosity for the Al_2_O_3_ nanofluid occurred at 0.1 weight percent of SDBS and the CuO nanofluid reached a minimum viscosity at 0.15 weight percent SDBS. Das et al. [44] also performed measurements on the viscosity of water-based nanofluids containing TiO_2_ nanoparticles and using CTAB and SDS as surfactants. The viscosity measurements were also taken across a range of temperatures from 20 to 60 °C using a DV-II + Pro standard programmable viscometer. The viscosity measurements were made using volume fractions of 0.1%, 0.5%, and 1% TiO_2_ nanoparticles. The viscosity was almost the same for the TiO_2_–CTAB–water nanofluid and the TiO_2_–SDS–water nanofluid. In Figure 13, the measurements for the thermal conductivity and viscosity of TiO_2_–water nanofluids with SDS and CTAB surfactants are shown using measurements [44]. The measurements for the TiO_2_–water nanofluids with surfactant at 1% volume concentration are also compared to measurements [34] for the thermal conductivity of TiO_2_–water nanofluids without surfactant and measurements [13] for the viscosity of TiO_2_–water nanofluids without surfactant. It can be seen that the thermal conductivity of both nanofluids containing surfactant have a higher thermal conductivity than the nanofluid without surfactant. Additionally, the TiO_2_–water nanofluid containing SDS has a higher thermal conductivity than the TiO_2_–water nanofluid containing CTAB [44]. In addition, both nanofluids containing surfactants also had a higher viscosity than the nanofluid without surfactant. Lastly, it can be seen that the TiO_2_–water nanofluids with CTAB and SDS surfactant have virtually the same viscosity. Freitas et al. [45] measured the thermal conductivity of nanofluids made using multiwalled carbon nanotubes (MWCNT) with water as a base fluid and several different surfactants used to improve the stability of the nanofluid. The surfactants used were Arabic gum (AG) at 0.25 weight percent, Triton’s X-100 (TrX) at 0.25 weight percent, and MWCNTs with a COOH acid group attached to them. The nanofluids tested had weight percentages of 0.125% and 1%, and it was observed that for each nanofluid as the weight percent increased the viscosity also increased. The nanofluids containing COOH had the highest viscosity followed by the nanofluids containing AG, followed by TrX. In Figure 14, the thermal conductivity and viscosity measurements for MWCNT–water nanofluids at a concentration of 1 wt % are shown [45]. The nanofluids contain 0.25 wt % Triton’s X-100(TrX), 0.25 weight percent Arabic Gum (AG) or COOH acid groups on the MWCNTs as surfactant. It can be seen that the nanofluid containing COOH has the highest thermal conductivity and viscosity. It was observed that the nanofluid containing AG has the lowest viscosity while the nanofluid containing TrX has the lowest thermal conductivity.

### 2.4. Effects of Temperature

**Figure 15:**
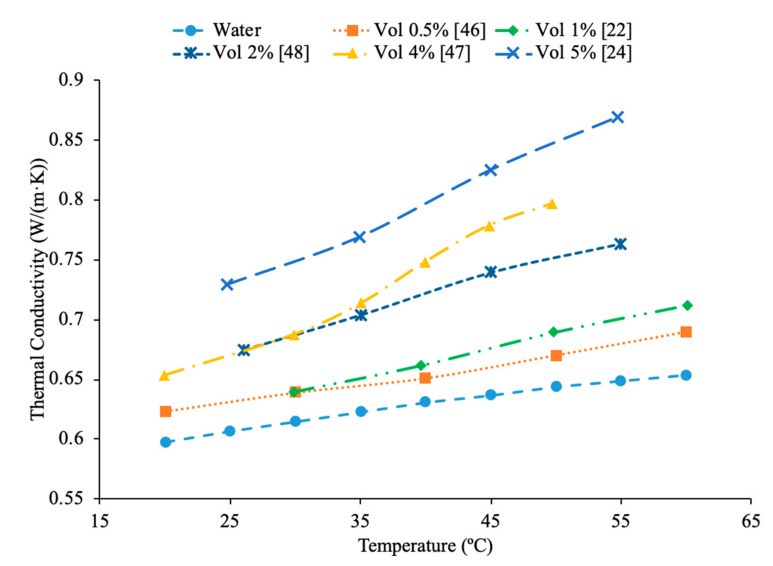
Thermal conductivity of Al_2_O_3_–water nanofluids as a function of temperature [22,24,46,47,48].

Temperature plays an important role in the effect of nanofluid thermal conductivity and viscosity as well. Researchers have done multiple studies with this topic and have concluded that a higher temperature associates with higher thermal conductivity of nanofluids. The nanofluids have shown that they are more temperature dependent than their base fluids suggesting that the observed increase in thermal conductivity is not just simply explained by the increase in the thermal conductivity of the base fluid [1]. Kwek et al. [24] performed measurements on the effect of temperature on thermal conductivity on Al_2_O_3_–water nanofluids. The nanofluids had volume fractions of 1%, 3%, and 5% and they were tested at temperatures ranging from 15 to 55 °C. The transient hotwire method was used to measure the thermal conductivity of the nanofluids. As the temperature increased, the thermal conductivity of the nanofluids also increased, and the effect of raising the temperature was greatest in the nanofluid with a 1% volume fraction. Chon et al. [22] also measured the effect of temperature on the thermal conductivity of Al_2_O_3_–water nanofluids. The measurements were made using the transient hotwire method on nanofluids with a 1% volume fraction and nanoparticles with diameters of 11 nm, 47 nm, and 150 nm. As the temperature of the nanofluids increased from 20 to 70 °C the thermal conductivity increased in all of the nanofluids. The increase in thermal conductivity can be as results of an increase in Brownian motion. The increased Brownian motion allows the particles to transfer energy throughout the fluid. Das et al. [47] measured the thermal conductivity of Al_2_O_3_–water nanofluids at volume fractions of 1% and 4% using the transient hotwire method. The measurements were taken across a range of temperatures from 21 to 51 °C and there was an increase in the thermal conductivity of the nanofluid with an increase in temperature. The nanofluids with a concentration 1% had an increase in thermal conductivity of 2% compared to the base fluid at 21 °C, while at 51 °C there was an increase of 10.8%. The increase in thermal conductivity with increase in temperature is caused by an increase of nanoparticle velocity and consequently Brownian motion at higher temperatures. Increase in Brownian motion would cause more energy transport. In Figure 15, the thermal conductivity of Al_2_O_3_–water nanofluids at different volume fractions as a function of temperature are compared. It can be concluded when looking at this graph, that the thermal conductivity of Al_2_O_3_–water nanofluids at any concentration shown increases with temperature, which is expected based on the conclusion earlier.

Additionally, Godson et al. [7] measured the effect of temperature on the thermal conductivity of Ag–water nanofluids using the transient hotwire method. The nanofluids studied had volume fractions of 0.3%, 0.6%, and 0.9% and the thermal conductivity was measured across temperatures ranging from 50 to 90 °C. An increase in Brownian motion at higher temperatures can be the reason for the increase in thermal conductivity. This is due to an increase in Brownian motion leading to an increase in particle collisions and energy transport. Krishnakumar et al. [49] measured the thermal conductivity of Al_2_O_3_–ethylene glycol nanofluids as a function of temperature. A KD2 Pro Thermal Property Analyzer, which relies on the transient hotwire method, was used to perform the measurements on nanofluids with volume fractions up to 1%. Each of the volume fractions tested showed an increase in thermal conductivity as the temperature increased. The increase in thermal conductivity was nonlinear and at higher temperature the percent increase in thermal conductivity of the nanofluid compared to the base fluid was higher. Maheshwary et al. [50] measured the effect of temperature on the thermal conductivity of TiO_2_–water nanofluids with weight percentages from 0.5% to 2.5%. At each weight percent tested the thermal conductivity increased as the temperature increased. Yu et al. [16] measured the effect of temperature on the thermal conductivity of AlN-ethylene glycol (EG) and AlN-propylene glycol (PG) nanofluids at a volume fraction of 10%. The transient short hotwire technique was used to perform measurements from 10 to 60 °C and it was observed that there was an increase in thermal conductivity as the temperature increased for both nanofluids tested. The increase in thermal conductivity measured in the nanofluid was similar to the increase that was found in just the base fluid as temperature increases. Shima et al. [51] used the transient hotwire method to measure the thermal conductivity of Fe_3_O_4_–water nanofluids as a function of temperature. These nanoparticles were produced by chemical co-precipitation. The measurements were taken using a nanofluid with a volume fraction of 1.02% across a range of temperatures from 25 to 50 °C and it was observed that as the temperature increased the thermal conductivity increased as well. The increase in thermal conductivity was attributed to an increase in Brownian motion as the temperature increased. An increase in Brownian motion leads to greater heat transfer in the base fluid. Esfe et al. [52] measured the thermal conductivity of Mg (OH)_2_–EG nanofluids as a function of temperature. The transient hotwire technique was used to measure the thermal conductivity of nanofluids with volume fraction from 0.1% to 2% across a range of temperatures from 24 to 55 °C. It was observed that as the temperature increased the thermal conductivity of the nanofluids increased, and the effect was more pronounced for higher volume fractions. The increase in thermal conductivity can be due to an increase in the kinetic energy of the particles at higher temperatures. Kinetic energy causes the nanoparticles to collide more creating more energy, which leads to better heat transfer abilities. Sundar et al. [53] measured the thermal conductivity of nanofluids made with a water–ethylene glycol (EG) mixture for a base liquid and Fe_3_O_4_ nanoparticles. These nanoparticles were synthesized by the chemical co-precipitation method by combining ferric chloride and ferrous chloride with distilled water. The sodium hydroxide could be added to the solution and the resulting precipitate could be filtered out and dried to obtain the nanoparticles. Details can be seen in referenced paper. The base liquids used were water and 20% EG, 40% EG, and 60% EG. The nanofluids had volume fractions from 0.2% to 2% and the measurements were taken from 20 to 60 °C using the transient hotwire method. Each of the nanofluids tested had an increase in thermal conductivity as the temperature increased, which is suggested to be because of an increase in Brownian motion. The increase in thermal conductivity based on temperature was highest in the nanofluids with a greater amount of water. The nanofluid with a base fluid that was 20% EG and a volume fraction of 2% had a 21.69% increase in thermal conductivity at 20 °C compared to the base fluid and a 46% increase at 60 °C. While the nanofluid with a base fluid that was 60% EG and a volume fraction of 2%, had a 15.7% increase in thermal conductivity compared to the base fluid at 20 °C and a 33% increase when compared to the base fluid at 60 °C. Effects of temperature on nanofluid thermal conductivity can be seen in Figure 1, Figure 6, Figure 7, Figure 8 and Figure 15.

**Figure 16:**
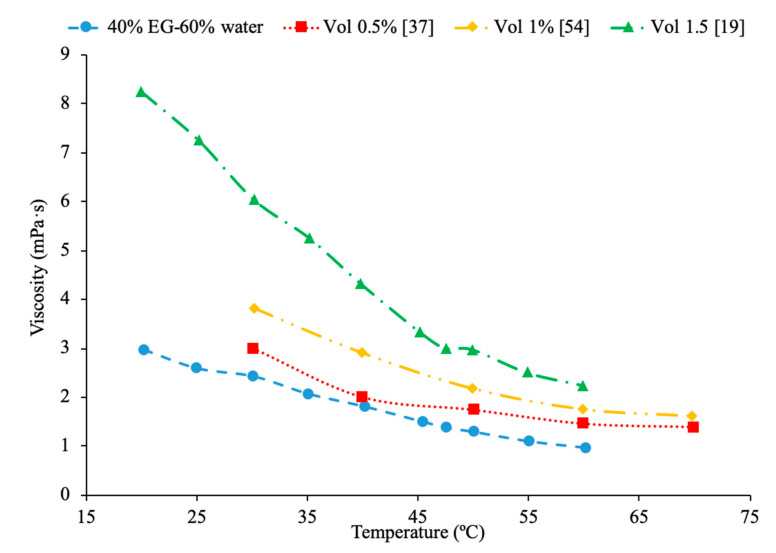
Viscosity as a function of temperature for various Al_2_O_3_-40% ethylene glycol (EG)-60% water volume concentrations [19,37,54].

In addition, Kwek et al. [24] performed measurements on the effect of temperature in the viscosity of Al_2_O_3_–water nanofluids. The nanofluids had volume fractions of 1%, 2%, and 3% of Al_2_O_3_ nanoparticles and the viscosity measurements were made using a Contraves LS 40 standard controlled rheometer. The measurements were taken at temperatures from 15 to 55 °C. The viscosity of the nanofluids decreases as the temperature increases, and the effect of increasing temperature diminishes as the temperature increases. The reduction in viscosity as the temperature increases can be due to the weakening of intermolecular forces. Nguyen et al. [12] measured the effect of temperature on the viscosity of CuO–water nanofluids. A ViscoLab450 Viscometer was used to measure the viscosity of nanofluids containing 29 nm nanoparticles at volume fractions of 1%, 4.5%, 7%, and 9% at temperatures from room temperature to about 75 °C. Each of the nanofluids tested showed a decrease in viscosity as the temperature increased, and the nanofluid with 9% volume fraction had the largest decrease in viscosity due to a temperature increase. As the temperature increased, the change in temperature also began to have a smaller effect on the viscosity, as the viscosity started to approach a constant value at high temperatures. Godson et al. [7] also measured the effect of temperature on the viscosity of Ag–water nanofluids. The viscosity was measured using a reverse flow viscometer, and nanofluids with 0.3%, 0.6%, and 0.9% volume fractions were measured. The measurements were taken across a range of temperatures from 50 to 90 °C and as the temperature increased, there was a decrease in the viscosity for each volume fraction tested. Krishnakumar et al. [49] measured the viscosity of Al_2_O_3_–ethylene glycol nanofluids as a function of temperature. The measurements were made using a Brookfield LVDV-II+Pro, plate and cone rheometer on nanofluids with 0.1% and 1% volume fraction with a nanoparticle diameter of 13 nm. The measurements were taken from 20 to 70 °C and there was a decrease in viscosity as the temperature increased. The decrease in viscosity was greater in the initial temperature values and then the viscosity eventually started to approach a constant. Shima et al. [51] measured the viscosity of Fe_3_O_4_–kerosene nanofluids as a function of temperature. The measurements were made using a rotational rheometer on nanofluids with volume fractions of 2.7%, 5%, and 9.5% across a range of temperatures from 25 to 50 °C. It was observed that as the temperature increased the viscosity of the nanofluid decreased. The decrease in viscosity that occurred as the temperature increased was consistent with the decrease in viscosity of the base fluid. Esfe et al. [52] measured the viscosity of Mg (OH)_2_-ethylene glycol nanofluids as a function of temperature using a Brookfield viscometer. The measurements were made using nanofluids with volume fractions from 0.1% to 2% and across a range of temperatures from 24 to 65 °C and it was observed that as the temperature of the nanofluid increased the viscosity decreased. The nanofluids with a higher concentration of nanoparticles experienced a greater decrease in viscosity as temperature increases. Pastoriza-Gallego et al. [18] measured the viscosity of Al_2_O_3_–EG nanofluids with volume fractions from 1% to 4.8% across a range of temperatures from 10 to 50 °C using a rotational viscometer. The viscosity of all the nanofluids tested decreased with an increase in temperature. Abdul Hamid et al. [37] measured the viscosity of Al_2_O_3_-40% ethylene glycol-60% water nanofluids at volume fractions from 0.5% to 2% using a Brookfield rheometer. The measurements were taken across a range of temperatures from 30 to 70 °C and there was a decrease in the viscosity of the nanofluids as the temperature increased. Chiam et al. [54] measured the viscosity Al_2_O_3_-40% ethylene glycol-60% water nanofluids at volume fractions from 0.2% to 1% using a Brookfield rheometer. The measurements were taken across a temperature range from 30 to 70 °C, and there was a decrease in viscosity as the temperature increased. The decrease in viscosity at high temperatures is caused by an increase in the intermolecular distance in the base liquid at high temperature. In Figure 16, the viscosity of Al_2_O_3_-40% ethylene glycol-60% water nanofluids at various volume concentrations as a function of temperature are compared. When looking at this figure, it can be shown that the viscosity of these nanofluids decrease with temperature, which is expected based on the reasoning earlier. Effects of temperature on nanofluid viscosity can be seen in Figure 2, Figure 10 and Figure 16.

**Figure 17:**
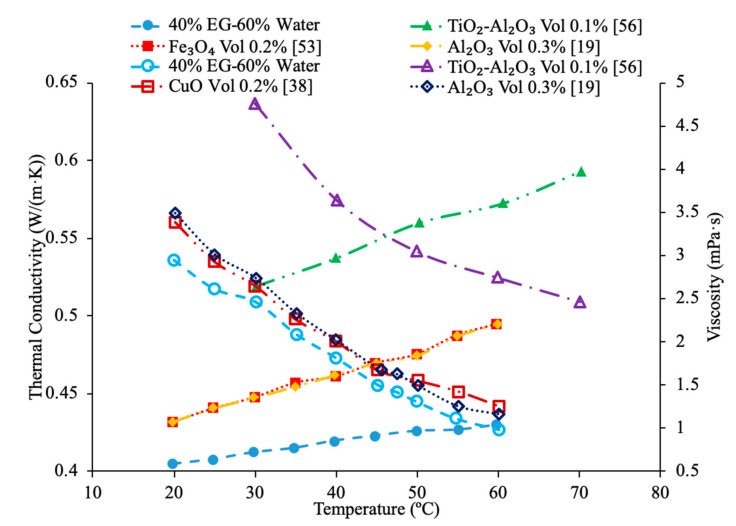
Thermal conductivity (increasing with temperature) and viscosity (decreasing with temperature) of nanofluids in a base liquid of 40% EG and 60% water [19,38,53,56].

**Figure 18:**
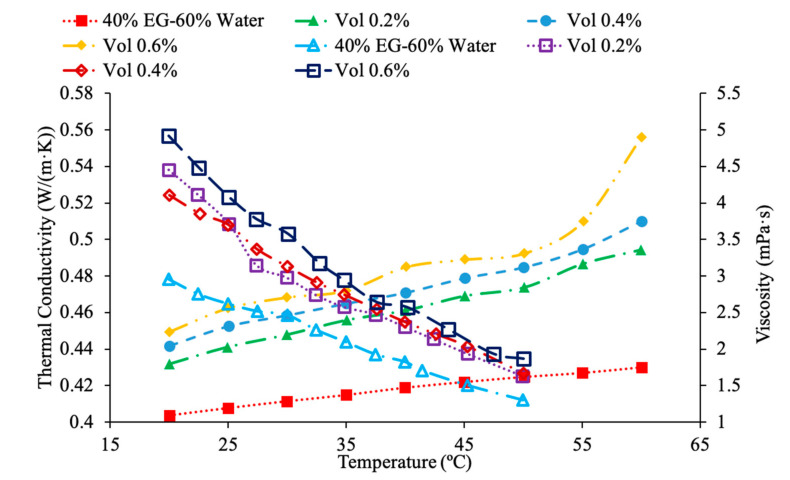
Thermal conductivity (increasing with temperature) and viscosity (decreasing with temperature) for Fe_3_O_4_-40% ethylene glycol (EG)-60% water at volume concentrations of 0.2%, 0.4%, and 0.6%. Thermal conductivity measurements from [53] and viscosity measurements from [57].

Various studies have concluded that the thermal conductivity of a nanofluid increases with temperature, while the viscosity decreases with temperature. Li et al. [55] measured both the thermal conductivity and viscosity of SiC-engine coolant nanofluids as a function of temperature. The measurements were taken from 10 to 50 °C and as the temperature increased the thermal conductivity increased while the viscosity decreased. The trend in the nanofluids as temperature increases followed the trend of the engine coolant base fluid which is 40% ethylene glycol and 60% water. Sundar et al. [4] measured the thermal conductivity and viscosity of Fe_3_O_4_–water nanofluids as a function of temperature. As the temperature increased, it was seen that the thermal conductivity increased while the viscosity decreased, which was shown in other studies. Gangadevi et al. [21] measured the viscosity and thermal conductivity of Al_2_O_3_, CuO and Al_2_O_3_–CuO hybrid nanofluids with water as a base liquid across temperatures from 20 to 60 °C. As the temperature increased, the thermal conductivity of the nanofluid increased, while the viscosity decreased. In Figure 17, the nanofluids in a base liquid of 40% EG and 60% water increases in thermal conductivity with an increase in temperature while there is a decrease in viscosity for an increase in temperature. Figure 18 shows the thermal conductivity and viscosity for nanofluids in a base liquid of 40% Fe_3_O_4_ and 60% EG at various volume concentrations of 0.2%, 0.4%, and 0.6%. It can be concluded when looking at the graph the 40% Fe_3_O_4_ and 60% EG at a volume concentration of 0.6% has the highest thermal conductivity and viscosity, rather the 0.2% has the lowest thermal conductivity and viscosity. It is also important to note that the trend stays consistent as temperature increases, thermal conductivity increases and viscosity decreases for the various nanofluids.

### 2.5. Effects of Base Fluid

Studies have concluded that the thermal conductivity of the base fluid has a direct relationship with the nanofluid’s thermal conductivity. Specifically, the thermal conductivity of the nanofluids increases in relation to the thermal conductivity of the base fluid used in the analysis. Wang et al. [58] performed measurements on the thermal conductivity of ionic liquid-based nanofluids also called ionanofluids. The base fluid used in these experiments was 1-hexyl-3-methylimidazolium tetra-fluorocarbonate ([HMIM]BF_4_) and nanoparticles made of both graphene and multi-walled carbon nanotubes (MWCNT) were used in the measurements. Graphene sheets were created by combining graphite powder with H_2_SO_4_ and KMnO_4_. Deionized water and H_2_O_2_ were then added to the mixture and the temperature was increased. The solution was then washed with HCl and the deionized water and the graphene oxide (GO) produced was allowed to dry. Finally, hydrazine hydrate was added to a GO-water mixture to convert the GO to graphene nanosheets. Details can be seen in referenced paper. The thermal conductivity measurements were performed using a Hot Disk TPS 2500 S thermal constants analyzer and the thermal conductivity was measured across a range of temperatures from 25 to 65 °C. The ([HMIM]BF_4_) ionanofluids behave similar to other base fluids in that as the temperature and the concentration of nanoparticles increases the thermal conductivity increases as well. The thermal conductivity increased by 11.8% to 12.3% for the ([HMIM]BF_4_) based ionic liquid-based nanofluid containing 0.03 weight% graphene and 15.5% to 18.6% for the nanofluid with 0.06% graphene as the temperature increased from 25 to 65 °C. The MWCNT ionic liquid-based nanofluids also showed increases in thermal conductivity as the temperature was raised from 25 to 65 °C. The MWCNT-([HMIM]BF_4_) with 0.03 weight percent MWCNT nanofluid showed increases in thermal conductivity of 3.9% to 8.4% and the 0.06 weight percent MWCNT nanofluid showed an increase in thermal conductivity of 13.0% to 13.2%. Some ionic liquids have the advantage of low vapor pressure and liquid properties across a wide range of temperatures. This allows ionic liquid based nanofluids to be used under a wide range of temperature and pressure conditions.

**Figure 19:**
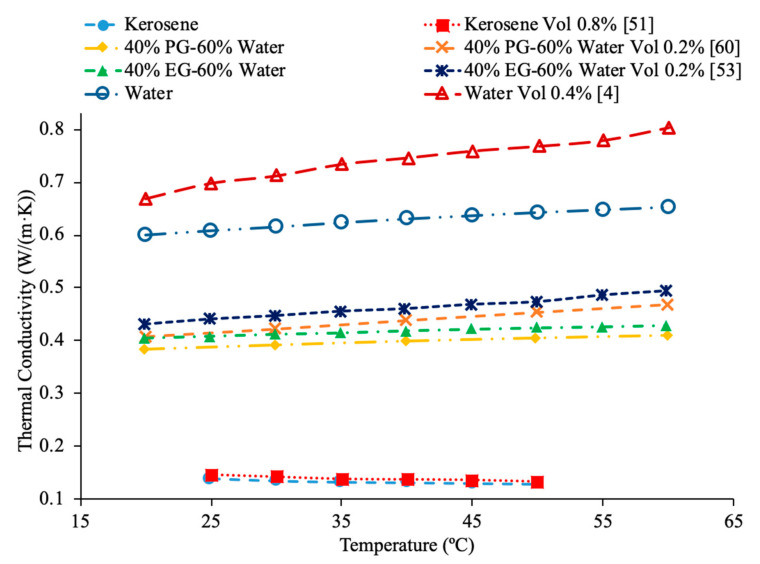
Thermal conductivity versus temperature for Fe_3_O_4_ nanofluids and various base fluids are shown [4,51,53,60].

**Figure 20:**
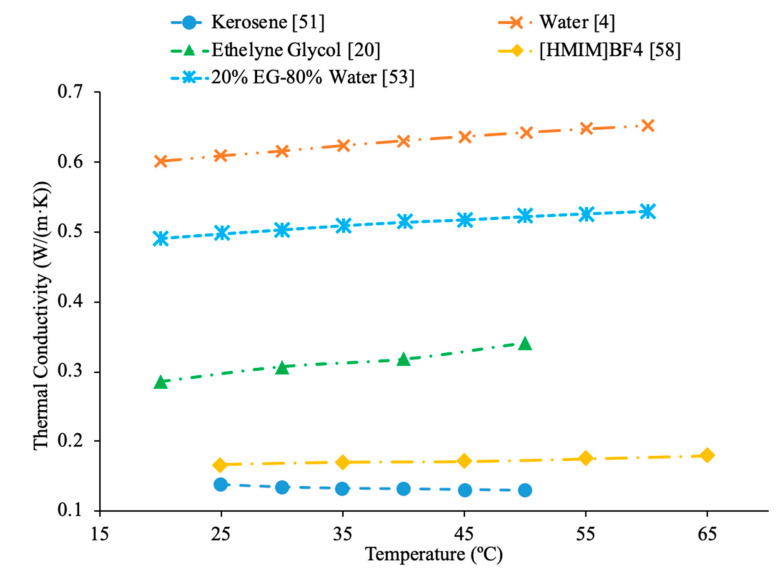
Thermal conductivity versus temperature for various base fluids [20,51,53,58].

AL-Waeli et al. [59] performed measurements using SiC nanoparticles in base fluids of water, 35% ethylene glycol (EG) and water, and 35% propylene glycol (PG) and water. The thermal conductivity measurements were made using a HOT DESK TPS 500 thermal conductivity meter. Cetyl-trichromyl ammonium bromide (CTAB) was used as a surfactant in the experiments to improve the stability of the nanofluids. When the thermal conductivities of all three SiC nanofluids with a weight concentration of 0.5% were measured across a range of temperatures from 25 to 60 °C there was no significant difference in the thermal conductivity. The change in base fluid did not affect the thermal conductivity in a meaningful way. Shima et al. [51] measure the thermal conductivity of Fe_3_O_4_–water, Fe_3_O_4_–kerosene, and Fe_3_O_4_–hexadecane nanofluids using the transient hotwire technique. The measurements were made on nanofluids with a volume fraction of 1.02% for the water-based nanofluid, 1.65% and 6.08% for the hexadecane based nanofluid, and 0.8%, 2.7%, 5%, 7.8%, and 9.5% for the kerosene based nanofluids. The thermal conductivity increased in all three nanofluids and the volume fraction increased. The thermal conductivity measurements were also taken across a range of temperatures from 25 to 50 °C and while the water-based nanofluid saw an increase in thermal conductivity as the temperature increased the kerosene and hexadecane nanofluids both saw a decrease in thermal conductivity as temperature increased. The trends observed in the thermal conductivity of the nanofluids are the same as those seen in the base fluids, as pure water increased in thermal conductivity as the temperature increased, while the pure kerosene and pure hexadecane both decreased in thermal conductivity as the temperature increased. Sundar et al. [19] measured the thermal conductivity of Al_2_O_3_ nanofluids in base liquids containing 20% ethylene glycol-80% water, 40% ethylene glycol-60% water, and 60% ethylene glycol-40% water. As the concentration of ethylene glycol increased, the thermal conductivity of the nanofluid decreased. This is because ethylene glycol has a lower thermal conductivity than water. As a result, as more ethylene glycol was added to the base liquid the thermal conductivity decreased. In Figure 19, the effect of temperature on the thermal conductivity of Fe_3_O_4_ nanofluids in base fluids of kerosene, water, 40% ethylene glycol-60% water, 40% propylene glycol-60%water is shown. The highest thermal conductivity is measured for the nanofluid in a water base liquid, while the lowest thermal conductivity is measured for the nanofluid in a kerosene base liquid. In Figure 20, the effect of temperature on thermal conductivity of several base fluids are compared, where it can be seen that water has the highest thermal conductivity. When ethylene is then added to the water to form a mixture the thermal conductivity is reduced. [HMIM]BF_4_ has a thermal conductivity lower than ethylene glycol, but greater than kerosene.

**Figure 21:**
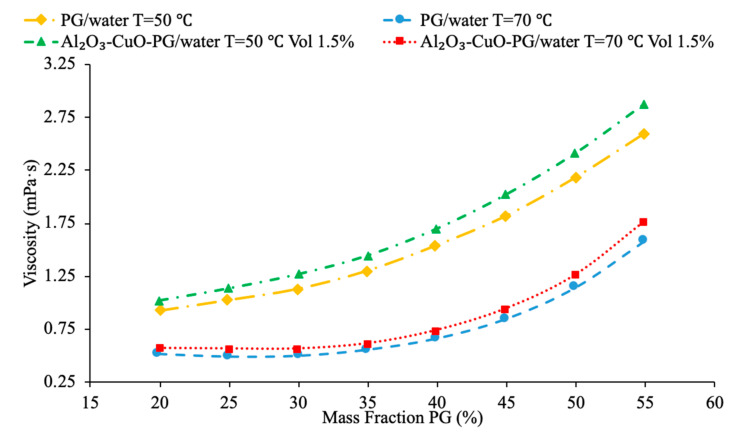
Viscosity of Al_2_O_3_/CuO nanofluid in base fluid of water and propylene glycol (PG) with changing mass fraction of PG [61].

Wang et al. [58] also performed measurements on viscosity of the MWCNT–([HMIM]BF_4_) and graphene–([HMIM]BF_4_) nanofluids. The viscosity measurements were taken using a DV-2+Pro viscometer and the viscosity was measured across a range of temperatures from 25 to 75 °C. Raising the temperature in the nanofluids also showed a decrease in viscosity. As the temperature increased from 25 to 75 °C, the viscosity of the 0.03 weight percent graphene nanofluid dropped from 217.4 mPa*s down to 40.6 mPa*s. Unlike most base fluids the viscosity of the ionic liquid-based nanofluid made with ([HMIM]BF_4_) actually had a lower viscosity than pure ([HMIM]BF_4_). This can be caused by the self-lubrication of the MWCNTs and graphene in the base fluid. AL-Waeli et al. [59] also performed viscosity measurements on the SiC-water, SiC-35% ethylene glycol-65% water, and SiC-35% propylene glycol-65% water nanofluids. The viscosity measurements were made using a Brookfield Model: LVDV-III ultra-programmable rheometer. The viscosity did change depending on the base fluid. The SiC-water, SiC-35% ethylene glycol-65% water, and SiC-35% propylene glycol-65% water showed 0.063%, 12.66%, and 16.66% increases in viscosity, respectively, compared to pure water. Both ethylene glycol and propylene glycol have a higher viscosity than water so the nanofluids made with a mixture of ethylene glycol and propylene glycol with water had a significant increase in viscosity compared to pure water. Shima et al. [51] measure the viscosity of Fe_3_O_4_–kerosene nanofluids using a rotational rheometer. The measurements were taken on nanofluids with volume fractions of 2.7%, 5%, and 9.5% and across a range of temperatures from 25 to 50 °C. It was observed that in the kerosene based nanofluid the viscosity increased with an increase in thermal conductivity, but decreased with an increase in temperature. Pure kerosene also displays a decrease in viscosity as temperature increases, meaning the nanofluid follows the same trend as that of the base fluid. Yu et al. [16] measured the viscosity of AlN-EG and AlN-PG nanofluids at volume fractions from 5% to 9% using a viscometer. The viscosity enhancement of the nanofluids compared to the base fluids is very close for both nanofluids. They both show an increase in viscosity as the concentration increases, and they both show the same increase in viscosity as compared to the base fluid. Abdul Hamid et al. [37] measured the viscosity of Al_2_O_3_ nanoparticles dispersed in a mixture of water and ethylene glycol using a rheometer. The three base liquids used were 40%:60%, 50%:50%, and 60%:40% water to ethylene glycol by volume. The measurements were taken across a range of temperatures from 30 to 70 °C and as the concentration of ethylene glycol increased, the viscosity of the nanofluid increased as well. This is because the viscosity of ethylene glycol is higher than the viscosity of water. All three fluids tested showed a decrease in viscosity as the temperature increased, therefore the nanofluid is following the same trend as the base fluid. Kumar et al. [61] measured the viscosity of hybrid nanofluids made with Al_2_O_3_ and CuO nanoparticles in equal parts by volume and base fluids made with water and ethylene glycol and water and propylene glycol were used. As the mass fraction of ethylene glycol and propylene glycol increased in each fluid the viscosity increased. This is because both ethylene glycol and propylene glycol have a higher viscosity than water. Additionally, it was studied that as the nanofluids made using a mixture of water and ethylene glycol were found to have a lower viscosity than those made using water and propylene glycol. This was due to ethylene glycol having a lower viscosity than propylene glycol. In Figure 21, the effect of viscosity of the nanofluid with water and propylene glycol as the base fluid is shown as a function of the mass percent of PG using measurements from [61]. Since PG has a higher viscosity than water, it can be seen that as the mass percent of propylene glycol increases the viscosity of the fluid increases.

**Figure 22:**
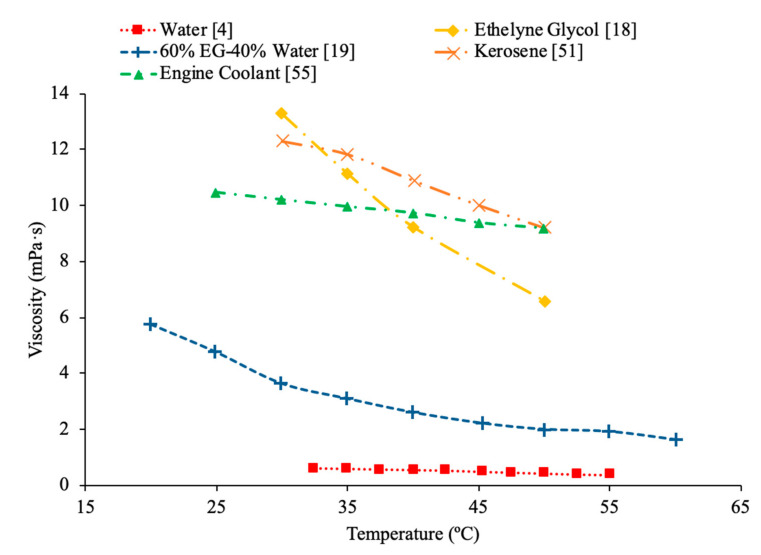
Comparison of viscosity of various base fluids as a function of temperature [18,19,51,55].

Timofeeva et al. [62] measured the viscosity of nanofluids made using SiC nanoparticles and base fluids of water and a 50%:50% mixture of water to ethylene glycol were used. The measurements were taken using nanoparticles with diameters of 16 nm, 29 nm, 66 nm, and 90 nm. The relative viscosity was looked at in these measurements. The relative viscosity of the water-based nanofluids was higher than that of the water–ethylene glycol mixture nanofluids. Therefore, the increase in viscosity caused by the addition of nanoparticles was higher in water than in the water–ethylene glycol mixture. Even though it has been shown in numerous other studies that water-based nanofluids have lower viscosity than water–ethylene glycol based nanofluids, water-based nanofluids have a higher relative viscosity. This suggests that the viscosity of water-based nanofluids is more affected by the addition of nanoparticles than the viscosity of water–ethylene glycol based nanofluids. In Figure 22, the viscosity of several base fluids as a function of temperature is shown. It can be concluded that all the base fluids looked at decrease in viscosity as the temperature increased. Ethylene glycol has the highest viscosity at low temperatures, but the viscosity significantly decreases as the temperature increases. At higher temperature engine coolant and kerosene have the highest viscosity. Water has the lowest viscosity, but the addition of ethylene glycol to the water to form a mixture raises the viscosity.

**Figure 23:**
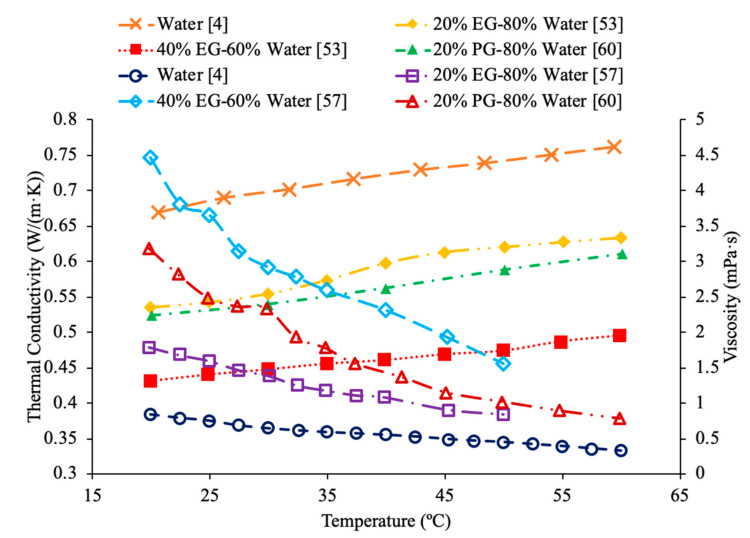
Thermal conductivity (increasing with temperature) and viscosity (decreasing with temperature) for Fe_3_O_4_ nanofluids at 0.2% volume fraction in base fluids of water, water–ethylene glycol (EG), and water–propylene glycol (PG) [53,57,60].

**Figure 24:**
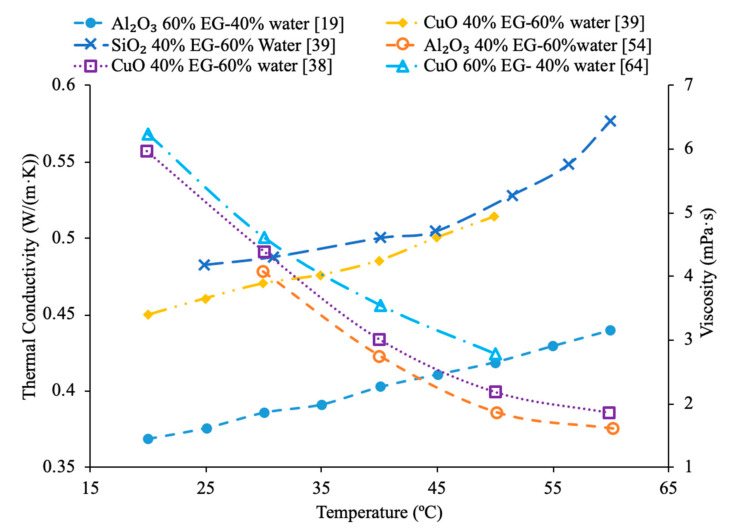
Thermal conductivity (increasing with temperature) and viscosity (decreasing with temperature) of various nanofluids with a base liquid that varies in percent ethylene glycol (EG) and percent water [19,38,39,54,63,64].

Figure 23 conveys thermal conductivity and viscosity versus temperature for Fe_3_O_4_ nanofluids at 0.2% volume fraction in various base fluids of water, water–ethylene glycol (EG), and water–propylene glycol (PG). It can be concluded that the Fe_3_O_4_ nanofluid with 60% EG has the highest viscosity, but the lowest thermal conductivity. Additionally, it can be seen that the Fe_3_O_4_ water-based nanofluid has the highest thermal conductivity, but the lowest viscosity. This was an interesting result, but signifies that the base liquid of EG is more viscous than the base liquids of PG and water. Additionally, these results show that the base liquid of water has better thermal conductivity properties than the base liquids of EG and PG. Figure 24 shows the thermal conductivity and viscosity as a function of temperature for various nanofluids that varies in the base liquid from the particular percent of ethylene glycol (EG) and percent water. When looking at the graph, it can be concluded that the SiO_2_ nanofluid with 40% EG and 60% water has a higher thermal conductivity than the SiO_2_ nanofluid with 60% EG and 40% water. This is due to the fact mentioned earlier that ethylene glycol has a lower thermal conductivity than water resulting in a lower thermal conductivity for the SiO_2_ nanofluid with a higher percentage of ethylene glycol. Additionally, it can be seen that the CuO nanofluid with 60% EG and 40% water has a higher viscosity than the CuO nanofluid with 40% EG and 60% water. This is due to ethylene glycol having a higher viscosity than water resulting in a higher viscosity for the CuO nanofluid with a higher percentage of ethylene glycol.

### 2.6. Effect of Shape of Nanoparticles

**Figure 25:**
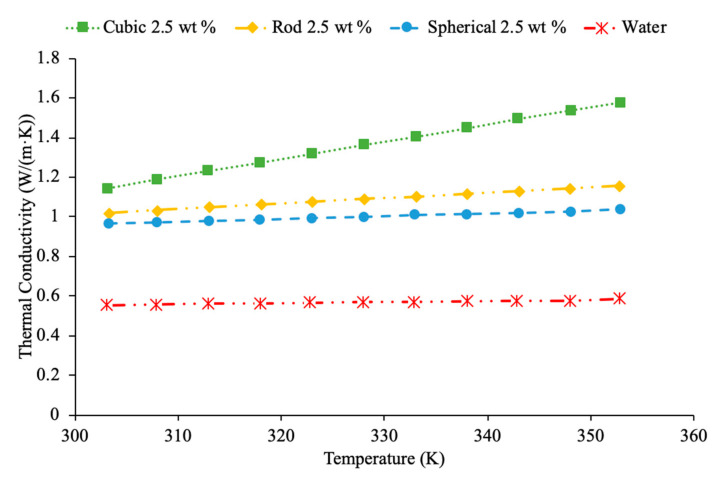
Thermal conductivity versus temperature for different shaped nanoparticles for TiO_2_ water-based nanofluids using measurements [50].

In addition, the shape of nanoparticles has been studied by researchers and there has been a common result that a higher surface area to volume ratio leads to higher thermal conductivity of the nanofluid. Maheshwary et al. [50] studied the effects of TiO_2_ water-based nanofluids when changing the shape of its nanoparticles. Three shapes were studied: cubic, rod, and spherical shaped nanoparticles, with the effect on the thermal conductivity of the nanofluid. Figure 25 shows the thermal conductivity as a function of temperature for TiO_2_ water-based nanofluid comparing cubic, rod, and sphere-shaped nanoparticles [50]. The cubic shaped nanoparticles have a higher thermal conductivity than the rod and spherical shaped nanoparticles. The cubic shaped nanoparticles have a higher surface area to volume ratio when compared to the rod and spherical shaped nanoparticles, but this does not always hold true as it depends on the diameter and height of the nanoparticles used when varying the shapes. A higher surface area to volume ratio means higher heat transfer as heat transfer depends on surface area. Main et al. [65] measured the thermal conductivity of sphere, rod and needle shaped Al_2_O_3_-1-Butyl-3-methylimidazolium bis(trifluoromethylsulfonyl) imide ((C4 mim) (NTf2)) nanofluids. The nanofluid containing needle shaped particles had the highest thermal conductivity. The high aspect ratio of the needle shaped nanoparticles can be a source of the high thermal conductivity as high aspect ratio relates to a high surface area to volume ratio. Zhu et al. [66] measured the thermal conductivity of CuO-dimethicone nanofluids with spherical and wire shaped nanoparticles. At a constant temperature of 25 °C, as the concentration of nanoparticles increased, the thermal conductivity increased linearly. The thermal conductivity of the nanofluid containing wire shaped particles was higher than the sphere-shaped particles. The small diameter particles provide the best increase in thermal conductivity due to their high aspect ratio in other research [65]. The high aspect ratio of the wires could explain their superior enhancement in thermal conductivity to the sphere-shaped particles as aspect ratio strongly correlates to the surface area to volume ratio.

**Figure 26:**
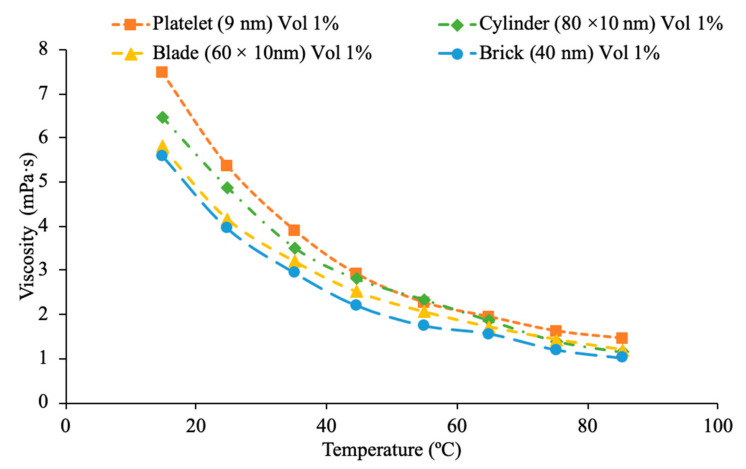
Viscosity versus temperature for different shapes of nanoparticles (platelets, cylinders, blades, and bricks) at 1% volume of Boehmite Alumina (AlO (OH)) nanofluid with 50% ethylene glycol (EG) and 50% water using measurements [67].

**Figure 27:**
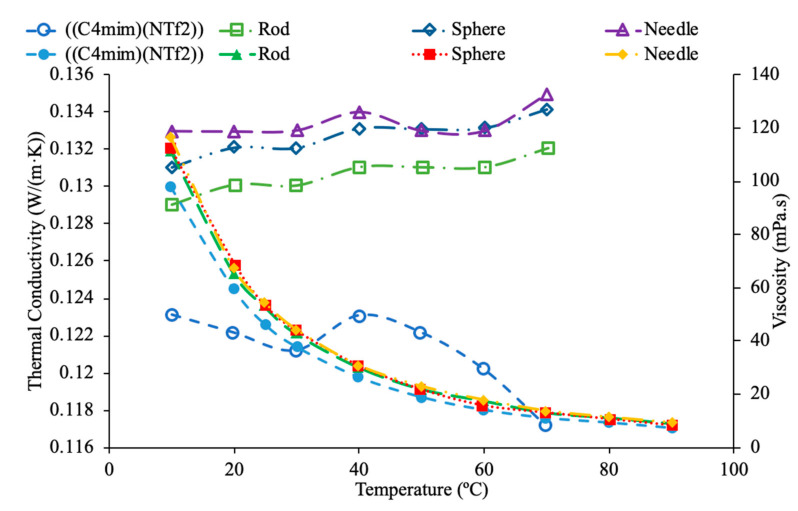
Thermal conductivity (increasing with temperature) and viscosity (decreasing with temperature) for 1-Butyl-3-methylimidazolium bis(trifluoromethylsulfonyl) imide ((C4mim) (NTf2)) based nanofluid with Al_2_O_3_ particles that are 2–5 nm diameter and 70–100 nm length needles, 10 nm diameter and 100 nm length rods, and 10 nm diameter spheres at 1 wt % [65].

Furthermore, the shape of nanoparticles plays also an important role in the viscosity of a nanofluid. Main et al. {65] measured the viscosity of sphere, rod and needle shaped Al_2_O_3_-1-butyl-3-methylimidazolium bis(trifluoromethylsulfonyl) imide ((C4 mim) (NTf2)) nanofluids. There was no significant difference in the viscosity of the three nanofluids. Each nanofluid had a viscosity greater than the base liquid and there was a decrease in the effect of the nanoparticles on the viscosity at high temperatures. Zhu et al. [66] measured the viscosity of CuO-dimethicone nanofluids with spherical and wire shaped nanoparticles. CuO nanowires were created by heating Cu foils in air, which then led to CuO nanowires forming on a Cu substrate. At a constant temperature of 25 °C, as the concentration of nanoparticles increased, the viscosity increased as well. There was, however, no significant difference in the viscosity of the two nanofluids with different shaped nanoparticles. It was proposed that since the concentration was less than 1% volume for all the nanofluids tested that the concentration was too low to measure the effect of the particle shape. There may be more of a relationship between particle shape and viscosity at high concentrations. Timofeeva et al. [67] also studied the effects of nanoparticle shape within Boehmite Alumina (AlO (OH)) nanofluids in a base liquid of 50% ethylene glycol and 50% water on viscosity. Specifically, the shapes that were analyzed were platelets, cylinders, blades, and bricks. The platelets were 9 nm, cylinders were 80 × 10 nm, blades were 60 × 10 nm, and the bricks were 40 nm. In Figure 26, the viscosity is conveyed as a function of temperature for the various shapes used at 1% vol using measurements [67]. It can be clearly concluded when looking at the graph that the platelet structure has the highest viscosity, while the brick structure has the lowest viscosity. In Figure 26, it can be seen that for each nanofluid tested as the temperature increases the viscosity decreases while the thermal conductivity increases. The needle and sphere nanoparticles provide the largest increase in thermal conductivity with the needle shaped particles being slightly higher. The viscosity however is not dependent on the particle shape. All three nanoparticle shapes used create a nanofluid with a viscosity higher than the base fluid, but there is no significant difference in the viscosity of the three nanofluids. Figure 27 conveys the thermal conductivity and viscosity as a function of temperature for various shaped nanofluids with ((C4 mim) (NTf2])) as a base liquid. Specifically, shaped nanoparticles of sphere, rod, and needle are compared with the measurements from [65]. It can be concluded from the graph that the difference in shape is not clear on its effects on the viscosity of the nanofluid as they all are crowding each other. On the other hand, it is evident that the needle shaped nanoparticles have a higher thermal conductivity than the sphere- and rod-shaped nanoparticles over the range of temperatures shown, except for the range between 50 to 60 °C where the sphere shaped has the highest thermal conductivity.

### 2.7. Effect of Material of Nanoparticles

**Figure 28:**
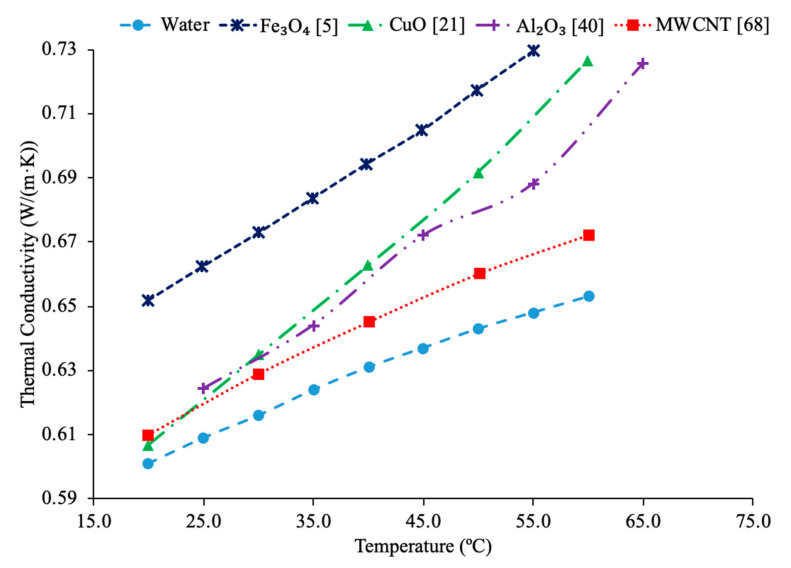
Thermal conductivity of CuO, Al_2_O_3_, Fe_3_O_4_ and MWCNT water-based nanofluid as a function of temperature at a volume concentration of 0.05% [5,21,40,68].

In addition to the effect of shape of nanoparticles on thermal conductivity and viscosity, there is also a correlation between the material of nanoparticles on its effect on thermal conductivity of the nanofluid. It has been determined that the thermal conductivity of the nanoparticle has an impact on the thermal conductivity of the nanofluid, providing one possible explanation for the variation of thermal conductivity based on the particle material. Gangadevi et al. [21] measured the thermal conductivity of both Al_2_O_3_–water and CuO–water nanofluids at volume fractions of 0.05%, 0.1% and 2%, across a range of temperatures from 20 to 60 °C using a KD2 Pro thermal property analyzer. Both nanofluids tested saw an increase in thermal conductivity with temperature, but the CuO–water nanofluid saw a greater increase than the Al_2_O_3_–water nanofluid. The Al_2_O_3_–water nanofluid increased in thermal conductivity by 11.23% for the 0.2% volume fraction as the temperature increased from 20 to 60 °C, while the CuO–water nanofluid at 0.2% volume fraction saw a 12.15% increase in thermal conductivity as temperature increased from 20 to 60 °C. Obviously, it is necessary to conduct more investigations to understand the correlation between the thermal conductivity of the nanoparticles and the thermal conductivity of the nanofluid. Li et al. [8] also found that the thermal conductivity of CuO–water nanofluids was greater than Al_2_O_3_–water nanofluids. Measurements on both nanofluids were made using the steady state method and it was shown that CuO–water nanofluids at 2% and 6% volume fraction both showed a greater enhancement in thermal conductivity across temperatures from 27.5 to 64.7 °C than the Al_2_O_3_–water nanofluid at 2% and 6% volume fraction. It was observed that the material of nanoparticles can affect the thermal conductivity of the nanofluid. Using the transient hotwire method Xing et al. [68] measured the thermal conductivity of nanofluids made using a variety of carbon nanotubes. The nanofluids all used water as a base liquid and short single wall carbon nanotubes (S-SWCNT), long single wall carbon nanotubes (L-SWCNT), and multiwall carbon nanotubes (MWCNT) were used. The three fluids were tested at 0.24% volume fraction across a range of temperatures from 10 to 60 °C, and it was seen that at 60 °C the L-SWCNT-water nanofluid had the highest thermal conductivity enhancement of 9.8%, followed by S-SWCNT with 5.07%, and MWCNT with 3.38%. Therefore, the L-SWCNT-water nanofluid has a higher thermal conductivity than S-SWCNT and MWCNT. L-SWCNT particles have a higher aspect ratio than the S-SWCNT and MWCNT which creates more contact between the base fluid, leading to higher heat transfer abilities. Figure 28 gives the thermal conductivity versus temperature for the various nanofluids tested. It can be concluded when looking at the figure, that the Fe_3_O_4_ nanofluid has the highest thermal conductivity compared to the rest of the nanofluids shown. It is also important to note that out of the nanofluids made using carbon nanotubes, the L-SWCNT-water nanofluid had the highest thermal conductivity followed by the S-SWCNT-water nanofluid and then the M-SWCNT-water nanofluid. Therefore, the particular type of material of nanoparticles used certainly plays a role in the thermal conductivity properties of the nanofluid.

**Figure 29:**
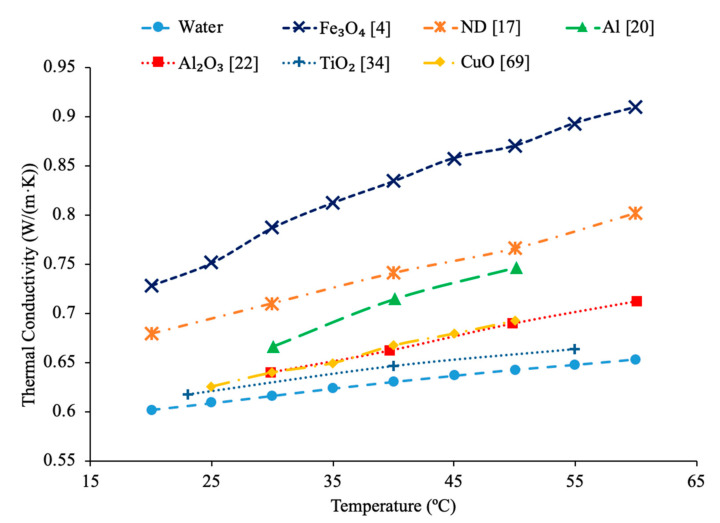
Thermal conductivity of CuO, Al_2_O_3_, Fe_3_O_4_, Al, TiO_2_ and ND water-based nanofluid as a function of temperature at a volume concentration of 1% [4,17,20,22,34,69].

Patel et al. [20] measured the thermal conductivity of Al-EG, Cu-EG, Al_2_O_3_–EG nanofluids using the transient hotwire method. The nanofluids were measured at a volume fraction of 2% across a temperature range of 20 to 50 °C. Each nanofluid increased in thermal conductivity as the temperature increased, with the highest thermal conductivity measured in the Cu-EG across the full range of temperatures. The smallest increase in thermal conductivity was measured in the nanofluid containing Al_2_O_3_ nanoparticles. The nanoparticle material has a significant impact on nanofluid thermal conductivity, however, it is very important to investigate how to engineer the nanofluid to have the highest impact of nanoparticle materials. Cu, Al, and Al_2_O_3_, nanoparticles have a thermal conductivity of 383 W/m·k, 204 W/m·k, and 27 W/m·k respectively. Therefore, the same trend seen in the thermal conductivity of the nanofluids is seen in the thermal conductivity of the nanoparticles. Figure 29 gives the thermal conductivity of various water-based nanofluids as a function of temperature. As can be seen by the graph, the Fe_3_O_4_ has the highest thermal conductivity, while TiO_2_ has the lowest conductivity and it can be clearly shown that it varies on the particular material of nanoparticles used. Therefore, the material of nanoparticles studied certainly plays a role on the thermal conductivity properties of a nanofluid.

**Figure 30:**
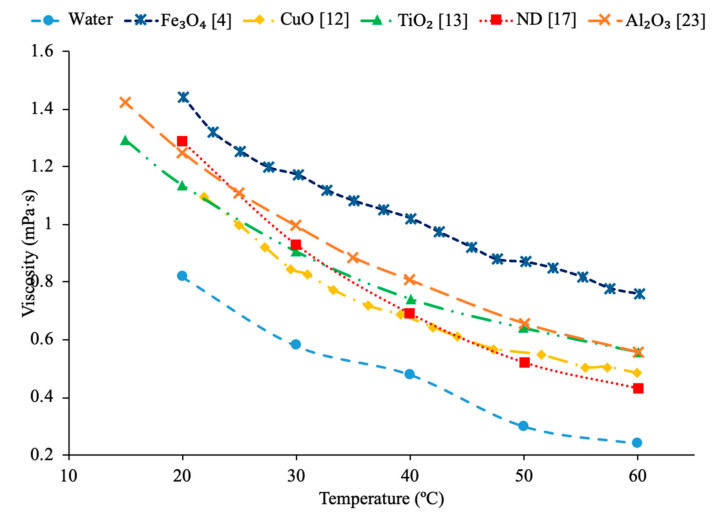
Viscosity of Al_2_O_3_, TiO_2_, ND, Fe_3_O_4_ and CuO water-based nanofluids at a volume fraction of 1% as a function of temperature [4,12,13,17,23].

**Figure 31:**
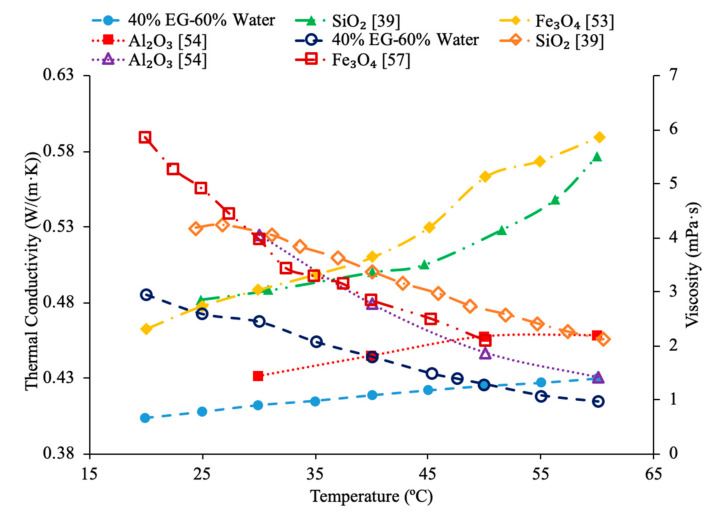
Thermal conductivity (increasing with temperature) and viscosity (decreasing with temperature) for SiO_2_, Fe_3_O_4_, and Al_2_O_3_ 40% ethylene glycol (EG)-60% water based nanofluids [19,39,53,57].

The material used in nanofluids has also been shown to affect the viscosity of the nanofluid. Yiamsawas et al. [13] measured the viscosity of Al_2_O_3_ and TiO_2_ water-based nanofluids using a capillary tube viscometer. The nanofluids tested had volume fractions from 1% to 4% and the measurements were taken across a range of temperatures from 15 to 60 °C. It was seen that across the range of temperatures tested the viscosity of both nanofluids decreased as temperature increased, but the viscosity of the Al_2_O_3_ nanofluids were higher than the TiO_2_ nanofluids. For the nanofluids with a 1% volume concentration the Al_2_O_3_ nanofluids had average viscosity that was 19.2% higher than the TiO_2_ nanofluid across the range of temperatures. Nguyen et al. [12] measured the viscosity of water-based CuO and Al_2_O_3_ nanofluids at volume fractions of 1%, 4%, 7% and around 9%. The measurements were taken using a piston type viscometer across a temperature range of about 22.5 to 70 °C. The CuO–water nanofluids had a higher viscosity than the Al_2_O_3_–water nanofluids. At a temperature of 30 °C the Al_2_O_3_ nanofluid had a viscosity of 0.8 mPa*s, 1.3 mPa*s, 1.7 mPa*s, and 3.6 mPa*s for volume fractions of 1%, 4%, 7%, and 9.4% respectively. At the same temperature the CuO nanofluid had a viscosity of 0.9 mPa*s, 1.5 mPa*s, 3.1 mPa*s and 6.5 mPa*s for particle concentrations of 1%, 4.5%, 7% and 9% respectively. Sundar et al. [17] measured the viscosity of ND–water nanofluids at volume fractions of 0.2%, 0.4%, 0.6%, 0.8%, and 1% using an A&D-vibro viscometer. The measurements were taken across a temperature range of 20 to 60 °C and the viscosity was measured to decrease as the temperature increased. Figure 30 gives the viscosity as a function of temperature for various nanofluids at a volume fraction of 1%. It can be seen in this figure that the Fe_3_O_4_ nanofluid had the highest viscosity, while at high temperatures the ND nanofluid had the lowest viscosity. At lower temperatures the CuO nanofluid had the lowest viscosity. Figure 31 shows the thermal conductivity and viscosity for various nanofluids as a function of temperature. When looking at Figure 31, it can be concluded that the Al_2_O_3_ had the highest viscosity up to about 40 °C and then SiO_2_ had the highest viscosity. Additionally, it can be seen that the SiO_2_ had the highest thermal conductivity below 30 degrees and then Fe_3_O_4_ had the highest thermal conductivity past the 30-degree mark. Therefore, the material of nanoparticles used within a nanofluid does affect the thermal conductivity and viscosity of a nanofluid.

### 2.8. Hybrid Nanofluids

**Figure 32:**
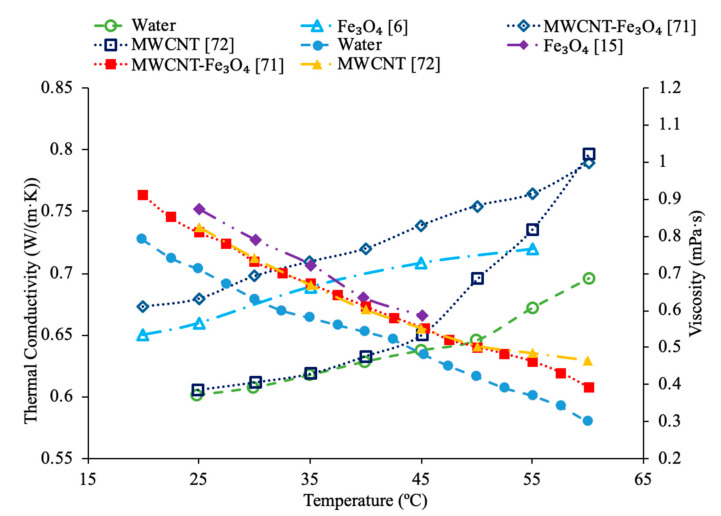
Thermal conductivity (increasing with temperature) and viscosity (decreasing with temperature) Fe_3_O_4_–MWCNT hybrid nanofluid, MWCNT and Fe_3_O_4_ with water as a base fluid at a volume of 0.1% [6,15,71,72].

Nanofluids are not limited to only containing one kind of nanoparticle. The mixture of a base liquid with two different nanoparticles and possible surfactant is called a hybrid nanofluid. Since there are several possible effects of a hybrid nanofluid, it is very important to engineer the hybrid nanofluids in a way that the second nanoparticles play a positive role in enhancing the thermal conductivity of the mixture of base liquid with the first and second nanoparticles. The second nanoparticles can enhance, deteriorate or have no significant effects on hybrid nanofluids. It is very important to engineer the hybrid nanofluids in a way that the second nanoparticles play a positive role in enhancing the thermal conductivity of a mixture of base liquid with the first and second nanoparticles. The nanoparticles found in hybrid nanofluids can be divided into three. Metal matrix nanocomposites, ceramic matrix nanocomposites, and polymer matrix nanocomposites [70]. These hybrid nanofluids demonstrate a similar relationship to standard nanofluids in regard to the effects of temperature and concentration on thermal conductivity. As the temperature of the hybrid nanofluid increases the thermal conductivity tends to increase. Sundar et al. [71] measured the thermal conductivity and viscosity for a multiwall carbon nanotube (MWCNT)–Fe_3_O_4_ water hybrid nanofluid at volume concentrations of 0.1%, and 0.3% and across a range of temperatures from 20 to 60 °C. Hybrid nanoparticles were made by the dispersion of carboxylated carbon nanotubes in distilled water. Then ferrous chloride and ferric chloride were added to the solution. Finally, sodium hydroxide was used as a reducing agent which resulted in the formation of MWCNT–Fe_3_O_4_ hybrid nanocomposite. Details can be seen in referenced paper. The thermal conductivity increased as the concentration of nanoparticles increased. As the temperature of the fluid increased, the thermal conductivity of the nanofluids with a higher concentration increased at a greater rate. As the temperature increased, the viscosity decreased. An increase in concentration was found to increase this viscosity. In Figure 32, the measurements for the thermal conductivity and viscosity of water-based MWCNT–Fe_3_O_4_ nanofluids [71] are compared to Fe_3_O_4_–water nanofluid thermal conductivity measurements [6], Fe_3_O_4_–water viscosity measurements [15], and MWCNT–water thermal conductivity and viscosity measurements [72], with all nanofluids at a concentration of 0.1% volume. It can be seen that across the range of temperatures, the MWCNT–Fe_3_O_4_ hybrid nanofluid has the highest thermal conductivity until about 60 °C. Then, the MWCNT–Fe_3_O_4_ and MWCNT have about the same thermal conductivity. The average thermal conductivity enhancements compared to the base fluid are 4.44% and 9.58% for the MWCNT and Fe_3_O_4_ nanofluids respectively. The average thermal conductivity for the MWCNT–Fe_3_O_4_ hybrid nanofluid is 15.25%. This demonstrates that the hybrid nanofluid has a thermal conductivity that is greater than either of the nanofluids containing only a single particle. The hybrid nanofluid viscosity is clearly lower than the Fe_3_O_4_ nanofluid and almost equal to the MWCNT nanofluid until about 50 °C. After 50 °C, the MWCNT and the hybrid nanofluid begin to diverge and the hybrid nanofluid clearly had a lower viscosity. Therefore, the MWCNT–Fe_3_O_4_ hybrid nanofluid had the lowest viscosity. Based on the combined effects of the thermal conductivity and viscosity it can be seen that the MWCNT–Fe_3_O_4_ nanofluid had a higher thermal conductivity and a lower viscosity than MWCNT or Fe_3_O_4_ nanofluids.

**Figure 33:**
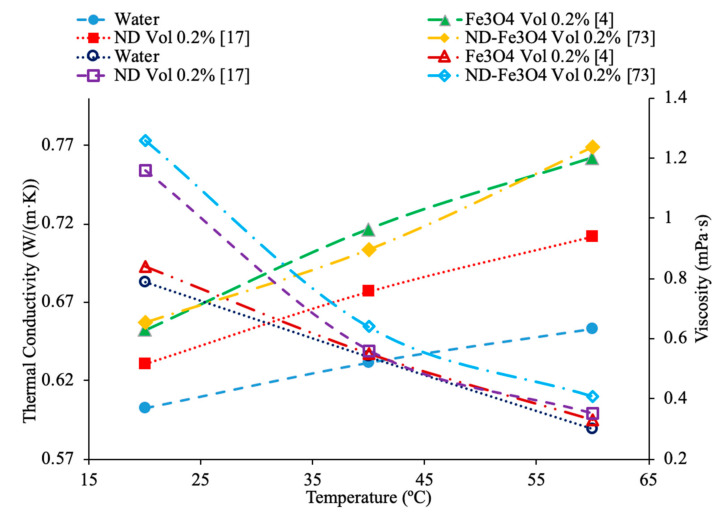
Thermal conductivity (increasing with temperature) and viscosity (decreasing with temperature) for ND–Fe_3_O_4_, ND, Fe_3_O_4_ water-based nanofluids at a volume concentration of 0.2% [4,17,73].

Sundar et al. [73] also measured the thermal conductivity and viscosity of nanodiamond (ND)–Fe_3_O_4_–water hybrid nanofluid. This was created by ND combining with FeCl_3_·6H_2_O and FeCl_2_·4H_2_O in distilled water and NaOH. The solution was then washed after magnetic stirring to remove excess ions. Details can be seen in referenced paper. It was observed that nanodiamond (ND)–Fe_3_O_4_ hybrid nanofluid provides better properties since nanodiamonds have a significantly higher thermal conductivity than the Fe_3_O_4_ nanoparticles. That would allow for a hybrid nanofluid to utilize both of those properties. The measurements were taken at 0.05%, 0.10%, and 0.20% volume fractions and across a range of temperatures of 20 °C, 40 °C, and 60 °C. As the temperature was raised from 20 to 60 °C, the ND–Fe_3_O_4_–water hybrid nanofluid showed a 11.1%, 13.1%, and 17.0% increase in thermal conductivity for volume fractions of 0.05%, 0.10%, and 0.20% respectively. As the temperature was raised from 20 to 60 °C, the ND–Fe_3_O_4_–water hybrid nanofluid showed a 69.1%, 68.3%, and 67.5% decrease in viscosity for volume fractions of 0.05%, 0.10%, and 0.20% respectively. In Figure 33, the measurements [73] for the thermal conductivity and viscosity at a volume fraction of 0.2% for ND–Fe_3_O_4_–water hybrid nanofluid is compared to measurements [4] for thermal conductivity and viscosity of Fe_3_O_4_–water nanofluid which is then compared to the thermal conductivity and viscosity measurements [17] for ND–water nanofluids. When looking at Figure 33, it can be concluded that across the range of temperatures the ND–Fe_3_O_4_ hybrid nanofluid [73] and the Fe_3_O_4_–water nanofluid [4] have approximately the same thermal conductivity, and have a higher thermal conductivity than the ND–water nanofluid [17]. The hybrid nanofluid has the highest viscosity while the ND and Fe_3_O_4_ nanofluid have approximately the same viscosity at temperatures above 40 °C. Therefore, despite the high thermal conductivity of nanodiamonds, the addition of nanodiamonds to the Fe_3_O_4_ nanofluid to create a hybrid nanofluid was not necessarily beneficial to the properties of the Fe_3_O_4_ nanofluid. At 60 °C, the ND–Fe_3_O_4_ hybrid nanofluid only had a 0.93% increase in thermal conductivity compared to the Fe_3_O_4_ nanofluid. The ND–Fe_3_O_4_ hybrid nanofluid also had a 24.24% increase in viscosity compared to the Fe_3_O_4_ nanofluid at 60 °C. In this case the hybrid nanofluid has minimal benefit to the thermal conductivity of the nanofluid compared to a nanofluid containing only one particle. The hybrid nanofluid does, however, have a significant impact on viscosity with the ND–Fe_3_O_4_ nanofluid having a much higher viscosity of the Fe_3_O_4_ nanofluid.

**Figure 34:**
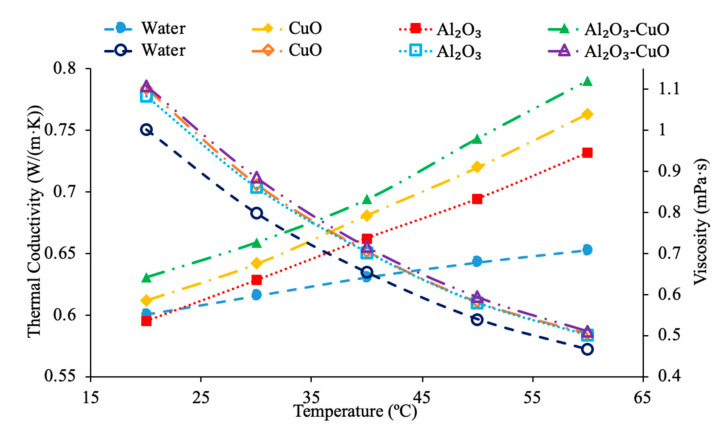
Thermal conductivity (increasing with temperature) and viscosity (decreasing with temperature) of water-based Al_2_O_3_, CuO, and Al_2_O_3_–CuO nanofluid at a volume concentration of 0.2% [21].

The properties of the ND–Fe_3_O_4_ nanofluid are not clearly better than the properties of the Fe_3_O_4_ nanofluid in this case Gangadevi et al. [21] measured the thermal conductivity of Al_2_O_3_, CuO and Al_2_O_3_–CuO hybrid nanofluids with water as a base liquid across temperatures from 20 to 60 °C using a KD2 Pro thermal property analyzer. The thermal conductivity of the Al_2_O_3_–CuO hybrid nanofluid had a higher thermal conductivity across the range of temperatures measured, than either the CuO or Al_2_O_3_ nanofluids. At a 0.2% vol–ume fraction and 60 °C the Al_2_O_3_–CuO hybrid nanofluid had a thermal conductivity 8% higher than the Al_2_O_3_ nanofluid and 3.5% higher than the CuO nanofluid. Gangadevi et al. [21] also measured the viscosity of Al_2_O_3_, CuO and Al_2_O_3_–CuO hybrid nanofluids with water as a base liquid across temperatures from 20 to 60 °C using a Brookfield viscometer. The Al_2_O_3_–CuO hybrid nanofluid had the highest viscosity of the three nanofluids, but not by a significant amount. At 60 °C, and a 0.2% volume fraction the Al_2_O_3_–CuO nanofluid had a viscosity that was only 1.43% higher than CuO and 2.20% higher than Al_2_O_3_. The results for the viscosity and thermal conductivity of each nanofluid at 0.2% volume fraction are given in Figure 34. In Figure 34, it can be seen that the Al_2_O_3_–CuO hybrid nanofluid had the highest thermal conductivity and viscosity across the range of temperatures measured. There is a greater difference in the thermal conductivity than in the viscosity of the three nanofluids. This suggests that the hybrid nanofluid has a greater effect on thermal conductivity than on viscosity.

**Figure 35:**
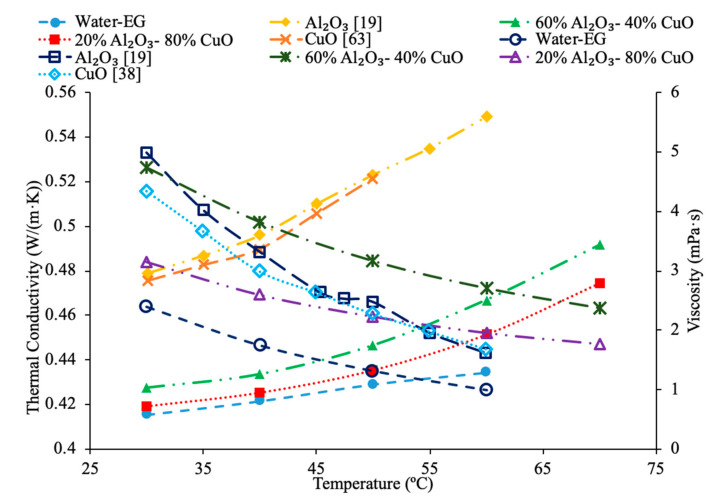
Thermal conductivity (increasing with temperature) and viscosity (decreasing with temperature) for Al_2_O_3_–CuO nanofluid at different ratios of Al_2_O_3_ to CuO [74] compared to Al_2_O_3_ and CuO nanofluids all in a base fluid of 40% ethylene glycol (EG)-60% water at volume fraction of 1% [19,38,63].

Wanatasanappan et al. [74] measured the thermal conductivity and viscosity of Al_2_O_3_: CuO water/EG hybrid nanofluids using different ratios of Al_2_O_3_ and CuO nanoparticles. The measurements were made across a range of temperatures from 30 to 70 °C using a KD2 Pro thermal analyzer for thermal conductivity and a Brookfield Rheometer for viscosity. The nanofluids had Al_2_O_3_: CuO ratios of 20:80, 40:60, 50:50, and 60:40, all were at a volume fraction of 1% and the base fluid had a ratio of 60% water to 40% EG. All the nanofluids tested had an increase in thermal conductivity as the temperature increased, and the highest thermal conductivity was measured for the nanofluid with a 60:40 ratio of Al_2_O_3_: CuO. At 70 °C, the 60:40 nanofluid had a 12.32% increase in thermal conductivity compared to the base fluid while the 50:50 nanofluid only had a 4.7% increase at the same temperature. The increased thermal conductivity with the increase in temperature is explained by the nanoparticles having higher kinetic energy at higher temperatures and therefore greater Brownian Motion. Brownian motion then creates more random movement of particles in the base fluid allowing for them to better transfer heat in the fluid. All of the nanofluids tested along with the base fluid had a decrease in viscosity with the increase in temperature. The nanofluid with the lowest viscosity across the range of temperatures was the nanofluid with a 20:80 ratio of Al_2_O_3_: CuO. The decrease in viscosity as the temperature increases is due do a weakening of the intermolecular forces between the nanoparticles and the base fluid. In Figure 35, the measurements [74] for thermal conductivity and viscosity of hybrid nanofluids with a 20:80 and 60:40 ratio of Al_2_O_3_: CuO are compared to measurements [19] for Al_2_O_3_ nanofluids and measurements [38,63] for CuO nanofluids. All the nanofluids in Figure 35 are at a volume fraction of 1% and in a base fluid of 40% ethylene glycol and 60% water. It can be seen that both the Al_2_O_3_ and CuO nanofluids have a higher thermal conductivity than the hybrid nanofluids. The viscosity trend is less clear as at low temperatures the 20% Al_2_O_3_-80% CuO hybrid nanofluid has the lowest viscosity. At higher temperatures the Al_2_O_3_ and CuO nanofluids have the lowest viscosity and are practically the same. Unlike what was seen by Gangadevi et al. [21], the use of hybrid nanofluid in this case had a negative effect on both the thermal conductivity and viscosity. The Al_2_O_3_–CuO hybrid nanofluid had a lower thermal conductivity than either the CuO or Al_2_O_3_ nanofluids. At least at high temperatures the hybrid nanofluid also had a higher viscosity than either of the nanofluids with a single particle. Therefore, it is not clear in all situations that the use of a hybrid nanoparticle will improve the properties of a nanofluid. Urmi et al. [56] measured the thermal conductivity and viscosity of Al_2_O_3_–TiO_2_–Water/EG hybrid nanofluids. The nanofluids tested were made with a base fluid with 40% EG and 60% water, and volume fractions of 0.02% to 0.1%. The ratio of the nanoparticles in the fluid was 20% Al_2_O_3_ to 80% TiO_2_. The measurements were made using a KD2 pro thermal property analyzer for thermal conductivity and a LVDV III Ultra Rheometer for viscosity across a range of temperatures from 30 to 80 °C. It was determined that as the temperature increased the thermal conductivity of the nanofluid increased while the viscosity decreased at all concentrations. The thermal conductivity of the Al_2_O_3_–TiO_2_ hybrid nanofluid was higher than the thermal conductivity of either the TiO_2_ or Al_2_O_3_ nanofluids. The viscosity of the hybrid nanofluid was greater than the viscosity of the Al_2_O_3_ nanofluid, but less than the viscosity of the TiO_2_ nanofluid. Therefore, the hybrid nanofluid clearly had better properties than the TiO_2_ nanofluid. The hybrid nanofluid also had better properties than the Al_2_O_3_ nanofluid with regard to thermal conductivity, but not viscosity. This relationship has been seen with several hybrid nanofluids where the use of a hybrid nanofluid increases both the thermal conductivity and viscosity compared to a nanofluid with a single particle used. Further investigation is necessary in order to understand the effects of second particles or engineer the nanofluid to have the best effects on thermal conductivity and viscosity. Additional details for the experimental data analyzed within this review paper are shown below in Table A1 in Appendix A.

## 3. Theoretical Models

These following theoretical models being presented are based on a single nanofluid and not hybrid nanofluids.

### 3.1. Effective Medium Theory

**Figure 36:**
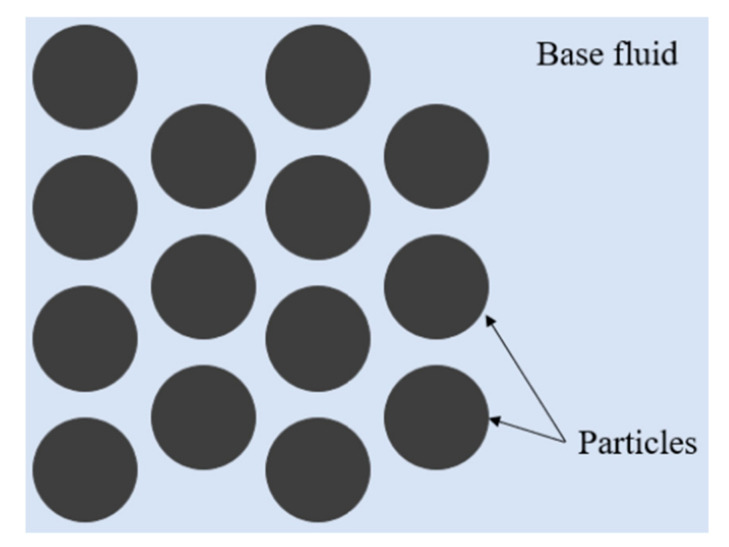
Effective medium theory static and homogenous assumption.

The effective medium theory (EMT) is a method used to predict the thermophysical properties of a fluid mixture. EMT models rely on the volume fraction of the solute and the properties of the particle and base fluid. EMT models assume a stationary and homogenous dispersion of particles within the base fluid as shown in Figure 36.

This static based theory was first developed by Maxwell [75] in 1873 where Maxwell experimented with micro sized particles and channels. His research resulted in the formulation of the following equation shown below in Equation (1).

(1) $$k_{nf}=\frac{k_{p}+2k_{bf}+2\phi (k_{p}-k_{bf)}}{k_{p}+2k_{bf}-\phi (k_{p}-k_{bf)}}k_{bf}$$

where $k_{p}\;$is the thermal conductivity of the nanoparticle, $k_{bf}\;$is the thermal conductivity of the base fluid, $k_{nf}\;$is the thermal conductivity of the nanofluid and *ϕ* the volume concentration of particles within a base fluid. This model is effective for calculating thermal conductivity of mixtures using micro and milli sized particles within a base fluid under relatively low concentrations of less than 2%. Recently, nanoparticles have been found to be more effective for increasing thermal conductivity than larger particles. Further work to try to improve EMT thermal conductivity models to include nanofluids was done by Timofeeva et al. [62]. Their research was based on experiments conducted using Al_2_O_3_–water and Al_2_O_3_–ethylene glycol nanofluids. In the model created by Timofeeva et al. [62], the nanoparticles are assumed to be static; the thermal conductivity of the particles is much greater than that of the base fluid, the particles are spherical, and the volume fraction is low. The EMT based model is given as:

(2) $$k_{nf}=\left(1+3\phi \right)k_{bf}$$

It is known that nanoparticles can form agglomerates due to interacting particles when dispersed in a liquid and Hamilton Crosser [76] believed that the shape of the agglomerates formed could impact the thermal conductivity of the nanofluids. Hamilton and Crosser [76] proposed considered the agglomerate shape and proposed the following equation:

(3) $$k_{nf}=k_{bf}[\frac{k_{p}+\left(n-1\right)k_{bf}-\left(n-1\right)\left(k_{bf}-k_{p}\right)\phi }{k_{p}+\left(n-1\right)k_{bf}+\left(k_{bf}-k_{p}\right)\phi }]$$

where n is an empirical constant based on the shape of the nanoparticle agglomerates formed. It was determined that n can be calculated based on the sphericity of the agglomerates. It was determined that n is three for spherical particles and six for cylindrical particles. A model was also created by Wasp et al. [77] which reduces down to the Hamilton Crosser [76] model when the particle agglomerates are described as spheres given as:

(4) $$k_{nf}=k_{bf}\frac{k_{p}+2k_{bf}-2\phi \left(k_{bf}-k_{p}\right)}{k_{p}+2k_{bf}+\phi \left(k_{bf}-k_{p}\right)}$$

Based on experimental work done using Ag–water nanofluids, Godson et al. [7] proposed the following model for the thermal conductivity of nanofluids as the other models that have been proposed predicted a thermal conductivity which was lower than the measured thermal conductivity. The model is given as:

(5) $$k_{nf}=k_{bf}\left(0.9692\phi +0.9508\right)$$

Sundar et al. [4] worked on looking at the effect of thermal conductivity with temperature. Sundar et al. [4] created his own experiment to analyze the effect of changes in temperature. A correlation was given to show his experimental empirical equation shown as:

(6) $$k_{nf}=k_{bf}{\left(1+10.5\phi \right)}^{0.1051}$$

With a concentration of ϕ < 2.0%, a temperature ranging 20 °C < T < 60 °C, an average deviation of 3.5%, and standard deviation of 4.2%, Sundar et al. [4] had the ability to create a stronger model of an EMT based equation that can show the thermal conductivity of a nanofluid as a function of temperature with volume fractions and temperatures within the provided ranges. This was accomplished by placing the given values for the thermal conductivities of the base fluid at a given temperature. There are more theoretical models and correlations related to effective medium approach that can be seen in references [2,4,53,62,67].

### 3.2. Nanolayer Method

**Figure 37:**
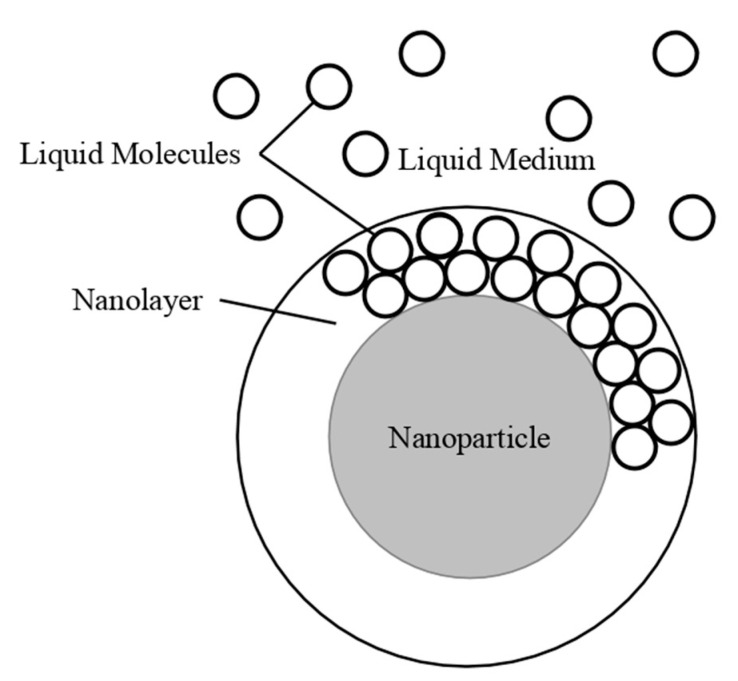
Depiction of nanolayer phenomena. Liquid molecules adhering to the surface of nanoparticles.

Layering of liquid molecules at the particle–liquid interface is a strong mechanism behind the thermal conductivity enhancements of nanofluids. The nanolayer is an ordered liquid structure that is formed on the outside of a nanoparticle that is caused by the interactions between the nanoparticle and the liquid molecules. This process of layering can be seen in the illustration in Figure 37.

The nanolayers’ molecule structure has a higher sense of order than the structure of the bulk liquid, which causes the nanolayer to be denser than the base fluid. Due to this nanolayer having an ordered state, it has a higher thermal conductivity than the bulk liquid and lower than the solid nanoparticle. This nanolayer acts as a thermal bridge between the base liquid and the nanoparticle helping to merge the thermal conductivities between them and increasing the overall thermal conductivity of the nanofluid.

Xie et al. [78] examined the structures formed by the nanolayer model and suggested with the assumption of a statistically homogeneous and isotropic nanolayer that:

(7) $$k_{nf}-k_{bf}=(3\Theta \phi +\frac{3\Theta^{2}\phi^{2}}{1-\Theta \phi })k_{bf}$$

where Θ is defined as,

(8) $$\Theta =\frac{\left(\frac{k_{nl}-k_{bf}}{k_{nl}+2k_{bf}}\right)[\left({1+\frac{\delta_{nl}}{r_{p}})}^{3}-\left[\frac{\left(k_{p}-k_{nl}\right)\left(k_{bf}+2k_{nl}\right)}{\left(k_{p}+2k_{nl}\right)\left(k_{bf}-k_{nl}\right)}\right]\right]}{{\left(1+\frac{\delta_{nl}}{r_{p}}\right)}^{3}+2\left[\left(\frac{k_{nl}-k_{bf}}{k_{nl}+2k_{bf}}\right)\left(\frac{k_{p}-k_{nl}}{k_{p}-2k_{nl}}\right)\right]}$$

where $k_{nl}$ is the thermal conductivity of the nanolayer, $r_{p}$ is the radius of nanoparticle, and $\delta_{nl}$ is the thickness of the nanolayer. The results from the provided equation show that the model is able to predict the effective thermal conductivities of different nanofluids. The following assumption that all the nanofluids were under by steady state heat conduction was taken into consideration. The physicochemical properties of the nanolayer are based on the: base fluid, suspended nanoparticles, and the interactions between them. Moreover, there are no expressions for determining the thermal conductivity of the nanolayer because of the complexities of the layer itself and the variables associated to it. The nanolayer thermal conductivity is mostly assumed to be a multiple of 2 or 3 of the base fluid’s thermal conductivity. The above equation is based on the general heat conduction equation in spherical coordinates, as well as, to the hard sphere fluid model. The equation created by Xie et al. [78] is mainly based on the assumption that the thermal conductivity of the nanolayer has a linear distribution. The thermal conductivity enhancement of the nanofluid increases inversely with the transition from a thermal insulation material to a conductive material. Further enhancements of the model would be to take into consideration of the addition of the factors such as shape, inclusion, and surface chemistry. A decrease in nanoparticle size and an increase in nanolayer thickness causes an increase in the effective thermal conductivity. A smaller nanoparticle size, causes the surface area to increase, which results in a larger nanolayer thickness. Therefore, this signifies that there is a correlation between nanoparticle size and the nanolayer itself.

Taking into consideration the effect of thickness, volume fraction, nanolayer effect, and thermal conductivity of the nanolayer and particle size, an equation was created by Leong et al. [79], as:

(9) $$k_{nf}=\frac{\left(k_{p}-k_{nl}\right)\phi k_{nl}[2\left(\beta_{nl}{)}^{3}-\beta^{3}+1\right]+\left(k_{p}+2k_{nl}\right)\beta_{nl}{}^{3}[\phi \beta^{3}\left(k_{nl}-k_{bf}\right)+k_{bf}]}{\beta_{nl}{}^{3}\left(k_{p}+2k_{nl}\right)-\left(k_{p}-k_{nl}\right)\phi \left(\beta_{nl}{}^{3}+\beta^{3}-1\right)}$$

where $\beta_{nl}$ and β are dimensionless nanolayer constants determined as,

(10) $$\beta_{nl}=1+\frac{\gamma }{2}$$

(11) β=1+γ

where γ is the ratio of nanolayer thickness to the nanoparticle radius and represented by:

(12) $$\gamma =\frac{\delta_{nl}}{r_{p}}$$

This equation created by Leong et al. [79] is used to calculate the effective thermal conductivity of the nanofluid based on nanoparticle size and the nanolayer effect. Additionally, if the nanolayer thickness is super small or if there is none, Equation (9) reduces to Maxwell [75] equation. The nanolayer itself has different thermo-physical components than the bulk liquid and the nanoparticle, solidifying itself as a separate part of the nanofluid. Leong et al. [79] divided the thermal conductivity into two parts which consisted of: (1) calculation of the gradients of temperature and the temperature fields, and (2) modeling of the effective thermal conductivity. There are three parts that make up the thermal conductivity of the nanoparticle which consist of: the thermal conductivity of the nanoparticle, the thermal conductivity of the base liquid, and the thermal conductivity of the nanolayer. The nanoparticles are sphere-shaped and they are separated at a distance where there are no interactions between the nanoparticles. The temperature fields are all continuous in the three parts.

Tinga et al. [80] had come up with a model that considers a complex dielectric constant of a multiphase nanofluid in 1973. The multiphases are made up of confocal ellipsoidal shell air–water–cellulose. This model was simplified to determine the thermal conductivity, and not the dielectric constant, by taking into consideration water as the nanolayer, air as the host medium, and the cellulose as a solid particle. The model is shown as:

(13) $$\frac{k_{nf}}{k_{bf}}=(1+\frac{3\phi \left[\left(\beta^{3}-1\right)\left(2k_{nl}+k_{p}\right)\left(k_{nl}-k_{bf}\right)-\left(k_{nl}-k_{p}\right)\left(2k_{nl}+k_{bf}\right)\right]}{\left(2k_{bf}+k_{nl}\right)\left(2k_{nl}+k_{p}\right)-\frac{2}{\beta^{3}-1}\left(k_{nl}-k_{bf}\right)\left(k_{nl}-k_{p}\right)-3\phi k_{nl}\left(k_{p}-k_{bf}\right)}$$

Yu and Choi [81] changed the Maxwell [75] equation to also have the effect of an ordered nanolayer which is shown as:

(14) $$\frac{k_{nf}}{k_{bf}}=(1+\frac{k_{p}+2k_{bf}+2\left(k_{p}-k_{bf}\right){\left(1-\gamma \right)}^{3}\phi }{\left(2k_{bf}+k_{nl}\right)\left(2k_{nl}+k_{p}\right)-\frac{2}{\beta^{3}-1}\left(k_{nl}-k_{bf}\right)\left(k_{nl}-k_{p}\right)-3\phi k_{nl}\left(k_{p}-k_{bf}\right)})$$

This equation proposed that a strongly ordered nanolayer acts as a thermal bridge for the thermal conductivities between the nanoparticle and the bulk liquid. The nanolayer plays an important role in the thermal conductivity of the nanofluids studied. The highly ordered nanolayer is proposed to have a higher thermal conductivity than the base liquid, which is an addition to the Maxwell [75] equation. This equation takes into consideration that the thermal energy transport within the nanofluid is diffusive. This is understood because of the average nanoparticle distance within the nanofluid being much larger than the mean free path of the molecules within the base liquid. Additionally, it was assumed that the nanolayer is placed around the nanoparticle to create an equivalent particle. The particle concentration is so low to cause no overlap when combining the equivalent particles. For larger nanoparticles, the nanolayer does not play as significant of a role causing the Yu and Choi [81] equation to be reduced to the Maxwell [75] equation. There are more theoretical models and correlations related to nanolayer approach that can be seen in references [8,10,11,82].

### 3.3. Brownian Model

**Figure 38:**
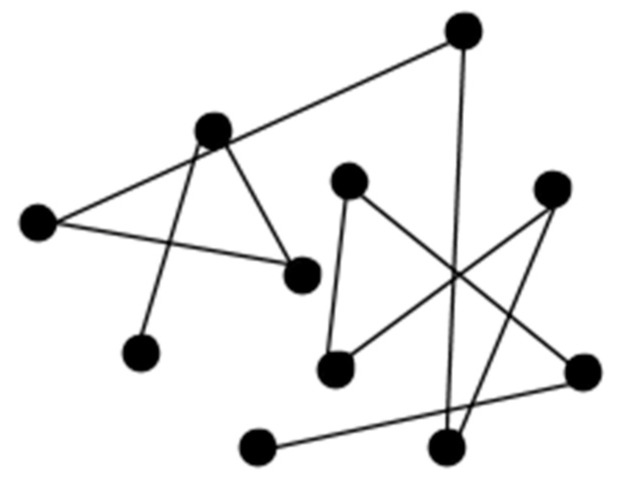
A representation of a singular nanoparticle under random motion caused by Brownian motion.

Brownian theory-based models consider the random motion of nanoparticles within a fluid from various collisions from molecules in the surrounding nanofluid, increasing the thermal conductivity of nanofluids. Convection locally can be caused by the Brownian random motion of the particles within the base fluid as well, which is shown in Figure 38.

Prasher et al. [83] determined the thermal conduction caused by Brownian motion and localized convection. They proposed the following equation:

(15) $$\frac{k_{nf}}{k_{bf}}=(1+\frac{k_{p}+2k_{bf}+2\left(k_{p}-k_{bf}\right){\left(1-\gamma \right)}^{3}\phi }{\left(2k_{bf}+k_{nl}\right)\left(2k_{nl}+k_{p}\right)-\frac{2}{\beta^{3}-1}\left(k_{nl}-k_{bf}\right)\left(k_{nl}-k_{p}\right)-3\varphi k_{nl}\left(k_{p}-k_{bf}\right)}$$

where:

(16) $$Re=\frac{1}{\nu }\sqrt{\frac{18k_{b}T}{\pi \rho d_{p}}}$$

As the particle size increases, the Reynolds number (Re) approaches zero. With this, the Equation (15) should reduce to the Maxwell [75] equation. The constants $k_{bf},v,d_{p},\rho ,$ and *T* are the Boltzmann constant, kinematic viscosity of the liquid, diameter of the nanoparticle, density, and temperature, respectively. The constants Pr, Bi, and A are the Prandtl number of the base fluid, the Biot number of the nanoparticle, and an empirical constant, respectively. The constant $k_{m}$ is the matrix conductivity which is created from the convection of the motion of a single sphere and is represented as:

(17) $$k_{m}=k_{bf}[1+\frac{1}{4Re}Pr]$$

The Biot number of the nanoparticle is represented by the following equation:

(18) $$Bi=\frac{2R_{b}k_{m}}{d_{p}}$$

where $R_{b}\;$is the thermal boundary resistance between various fluids and nanoparticles. Prasher et al. [83] presented that there are additional components to consider than just conduction when determining the thermal conductivity of nanofluids. They proposed that the Brownian motion of nanoparticles and convection need to also be considered when determining the thermal conductivity of a nanofluid. They provided several components for the thermal energy transfer within nanofluids which consisted of: an interparticle potential, translational Brownian motion, and the convection in the nanofluid due to Brownian motion of the particles. Their data supported that the local convection from the Brownian motion of the nanoparticles had the biggest impact on the thermal conduction of the nanofluid. The effect of the interfacial layering did not have much of an impact for larger nanoparticles. As the nanoparticle size increased, the effects of layering and convection are diminished and, therefore, the conduction models are sufficient enough in predicting the thermal conductivity of the nanofluid. Due to nanoparticles being so small, the existence of the interparticle surface forces become important and can provide various energy modes for thermal transport. The Brownian–Reynolds number can be considered when looking at the convective forces of the nanoparticles. In particular, the Brownian–Reynolds number is applied to be able to determine the matrix thermal conductivity that is formed by the convection from the motion of a single sphere.

Koo and Kleinstreuer [84] created a model that uses the effects particle volume fraction, temperature dependence, and nanoparticle size. In addition, the particle phase and properties of the base liquids are taken into consideration to determine the Brownian motion of the nanoparticles, and is shown in the following equation:

(19) $$k_{nf}=[\frac{k_{p}+2k_{bf}-2\phi \left(k_{bf}-k_{p}\right)}{k_{p}+2k_{bf}+\phi \left(k_{bf}-k_{p}\right)}]k_{bf}+[5\times 10^{4}\zeta \phi \rho_{bf}c_{bf}\sqrt{\frac{k_{b}T}{\rho_{p}d_{p}}}f\left(T,\phi ,etc.\right)]k_{bf}$$

where:

(20) $$f\left(T,\phi \right)=\left(-6.04\phi +0.4705\right)T+\left(1722.3\phi -134.63\right)$$

where ζ is an empirical constant that is based on the volume fraction of solute. Koo and Kleinstreur [84] determined the following ζ properties from the experimental data they analyzed. Table 1 below displays their findings from their experimental data.

Brownian motion was proposed to cause micro-mixing that enhanced the thermal conductivity. The enhanced thermal conductivity was considered as additive to the thermal conductivity of a static dilute suspension. Experimental results have confirmed that Brownian motion is more significant at higher temperatures due to a large amount of energy and vibration in the fluid. Two nanoparticles were used in the calculations to determine the enhancement in thermal conductivity as a result of Brownian motion. The nanoparticles were placed in two different temperature fields with time averaged motions. The average distance for the particles to move in the same direction without any deviation in its path was varied. It was assumed that there was steady flow in the Stokes regime in order to estimate the region of the affected fluid volume. This allowed for a quantitative comparison of induced heat transfer and micro-mixing. The shape of the nanoparticle affects the shape and size of the fluid volume. Therefore, Brownian motion not only contributes to the motion of the nanoparticles, but the larger fluid body, which contributes to micro mixing. Additionally, interparticle potential at high and low concentrations was considered, and it was determined that low concentrations show low dependency on thermal conductivity since there is less particle interaction. Curve fitting was used from experimental analysis to determine the models because of the complexity of the considered effects.

The Koo and Kleinstreuer [84] model was improved upon by Vajiha and Das [85] by modifying the empirical correlations. The model by Kool and Kleinstreuer [84] closely matched the 133 experimental data points collected by Vajiha and Das [85]. Vajiha and Das [85] used three different types of nanofluids in their experimental work. Although the Koo and Kleinstreuer [84] model matched their data better than the other Brownian models, Vajiha and Das [85] sought to expand the range of validity of the model. The same base of Equation (20) was used by Vajiha and Das [85] in their model, but the empirical correlations were modified to cover a wider range of temperatures and concentrations. Table 2 shows these empirical correlations.

(21) $$f\left(T,\phi \right)=\left(0.028217\phi +0.003917\right)\frac{T}{T_{0}}+\left(0.030669\phi -0.00391123\right)$$

Vajiha and Das [85] were able to increase the range of temperature and concentration as well as the nanoparticle materials that could be considered. This effectively increased the range of validity for predicting nanofluid thermal conductivity.

Chon et al. [22] used linear regression analysis and Buckingham-Pi theorem on Al_2_O_3_–water nanofluid data to determine a model for the thermal conductivity of nanofluids. This model which also considered the Brownian motion of nanoparticles is given as:

(22) $$k_{nf}=(1+64.7\phi^{0.746}{\left(\frac{d_{bf}}{d_{p}}\right)}^{0.369}(\frac{k_{np}}{k_{bf}}{)}^{0.746}Pr^{0.9955}Re^{1.2321})k_{bf}$$

where:

(23) $$Pr=\frac{c_{pbf}\mu_{bf}}{k_{bf}}$$

(24) $$Re=\frac{\rho_{bf}k_{b}T}{3\pi \mu_{bf}{}^{2}l_{bf}}$$

where $l_{bf},{µ}_{bf,}$ and $c_{pbf}$ are the mean free path, specific heat, and dynamic viscosity of the base fluid. Chon et al. [22] consider a mean free path value of 0.17 nm over the entire temperature range throughout their work.

Patel et al. [20] collected experimental data for Al_2_O_3_ and CuO nanofluids with base liquids of water, ethylene glycol and oil. Then a nonlinear regression model was detained from this large set of data. An increase in thermal conductivity as the particles size decreased was attributed to greater Brownian motion of the smaller nanoparticles and the high surface area of the smaller particles. Since heat transfer is a function of surface area and smaller nanoparticles have a higher surface area to volume ratio, the heat transfer in the fluid will increase with smaller nanoparticles. This will then result in an increase in the thermal conductivity of the nanofluid. The model determined by Patel et al. (20) is given as:

(25) $$k_{nf}=k_{bf}(1+0.135{\left(\frac{k_{p}}{k_{bf}}\right)}^{0.273}\phi^{0.467}{\left(\frac{T}{20}\right)}^{0.547}(\frac{100}{d_{p}}{)}^{0.234})$$

This model is valid for nanoparticles with thermal conductivities ranging from 20–400 W/(m·K) and nanoparticle size from 10–150 nm. The range of allowable base fluid thermal conductivity is 0.1–0.7 W/(m·K). The valid temperatures and volume fraction ranges are 20–60 °C and 0.1–3%, respectively. Additionally, the particle size is considered in nanometers and the temperature is considered in Celsius.

Brownian motion as one of the main mechanisms of thermal conductivity enhancements, was also considered by Corcione [86]. Corcione [86] recognized that the Maxwell [75] and Hamilton Crosser [76] models failed to accurately predict the thermal conductivity when compared to experimental data over a range of temperatures. Corcione considered the effect of dimensionless numbers such as the Reynolds number and Prandtl number of the base fluid as the temperature changed in the model. Corcione [86] considered data for Al_2_O_3_, CuO, and TiO_2_ nanofluids with base liquids of water an ethylene glycol. The data was compiled from 13 sources such as Chon et al. [22], Eastman et al. [87], Lee et al. [88], and Murshed et al. [89]. The model determined from the data is given as:

(26) $$k_{nf}=k_{bf}(1+4.4Re^{0.4}Pr^{0.66}{\left(\frac{T}{T_{fr}}\right)}^{10}{\left(\frac{k_{p}}{k_{bf}}\right)}^{0.03}\phi^{0.66}$$

where $T_{fr}$ is the freezing temperature of the base fluid and the Reynolds number is given as:

(27) $$Re=\frac{2\rho_{bf}k_{bf}T}{\pi \mu_{bf}{}^{2}d_{p}}$$

The validity of the models is limited based on the data used to determine the model. The model can be used for a temperature range of 21–51 °C and a volume fraction range of 0–9%. Additional Brownian motion thermal conductivity models are given in references [8,89,90,91,92].

### 3.4. Empirically Determined Viscosity Models

While increasing thermal conductivity of a nanofluid is desirable to improve the heat transfer capabilities of the nanofluid, the effect on viscosity of the nanofluid must also be considered. The pumping power required for a working fluid is related to the viscosity of the fluid. Therefore, understating the viscosity of a fluid is necessary to optimize the system in which it will be used. Several of the theoretical models to describe the viscosity of a nanofluid will be discussed in this section.

One of the first attempts to model the viscosity of a fluid-particle mixture was done by Einstein [93] in the 1900s. This early model was used to predict the effective viscosity of mixture fluids and was applicable in limited low volume concentration applications *ϕ* < 0.02%.

(28) $$\mu_{nf}=\left(1+2.5\phi \right)\mu_{bf}$$

where $\mu_{nf}$ is the viscosity of the nanofluid and $\mu_{bf}\;$is the viscosity of the base fluid. The model proposed is most accurate at low particle concentrations. Brinkman [94] attempted to expand on the model proposed by Einstein [93] based on the work done by [93] in the following model:

(29) $$\mu_{nf}=\mu_{bf}(\frac{1}{{\left(1-\phi \right)}^{2.5}})$$

The model proposed by Brinkman [94] was found to be effective for volume fractions up to 2%. Based on experimental work done using Al_2_O_3_–water nanofluids Wang et al. [95] proposed the following model:

(30) $$\mu_{nf}=\mu_{bf}\left(123\phi^{2}+7.3\phi +1\right)$$

Based on experimental data collected using Ag–water nanofluids, Godson et al. [7] proposed the following model through regression analysis and line of best fit:

(31) $$\mu_{nf}=\mu_{bf}\left(1.005+0.497\phi -0.1149\phi^{2}\right)$$

Recently, Maϊga et al. [96] used experimental data from Lee et al. [88], Eastman et al. [87], and Wang et al. [95] and curve fitting to determine a model to predict the viscosity of a specific nanofluid. The least square method was used in the curve fitting and forced convection flow of Al_2_O_3_–water and Al_2_O_3_–ethylene glycol nanofluids was considered. The model determined is given in Equation (32):

(32) $$\mu_{nf}=\mu_{bf}\left(1+7.3\phi -123\phi^{2}\right)$$

Nguyen et al. [12] also used curve fitting to determine models for the viscosity of nanofluids made with nanoparticles of a particular size and material. Experimental data was collected for 36 and 47 nm Al_2_O_3_–water nanofluids, as well as 29 nm CuO–water nanofluids. Then models were created for each of the three nanofluids based on the experimental data collected. Based on the experimental data the viscosity of Al_2_O_3_–water and CuO–water nanofluids are very similar until a volume fraction of about 4%. Beyond a volume fraction of 4% the CuO–water nanofluid increased significantly and diverged from the viscosity trend seen in the Al_2_O_3_–water nanofluid. The molecular structure of the mixture could explain the differences seen in the nanofluids made with a different material of nanoparticle. The process of dispersing the nanoparticles could also affect the viscosity. The correlations proposed by Nguyen et al. [12] for 47 nm Al_2_O_3_, 36 nm Al_2_O_3_ and 29 nm CuO respectively are given in Equations (33)–(35):

(33) $$\begin{array}{c} \mu_{nf}=\mu_{bf}\left(0.904e^{0.148\phi }\right)\\ \;\;47\;\mathrm{nm}\;{\mathrm{Al}}_{2}O_{3} \end{array}$$

(34) $$\begin{array}{c} \mu_{nf}=\mu_{bf}\left(1+0.025\phi -0.015\phi^{2}\right)\\ \;36\;\mathrm{nm}\;{\mathrm{Al}}_{2}O_{3} \end{array}$$

(35) $$\begin{array}{c} \mu_{nf}=\mu_{bf}\left(1.475-0.319\phi +0.051\phi^{2}+0.009\phi^{3}\right)\\ \;29\;\mathrm{nm}\;\mathrm{CuO} \end{array}$$

Rea et al. [97] collected experimental data for 50 nm Al_2_O_3_–water and 50 nm ZnO-water nanofluids in order to determine a model for the viscosity of nanofluids. The thermo-physical properties of the fluids were determined in a channel where the temperature was varied by the transient hotwire method. This allowed for the creation of models that considered both volume fraction and temperature. Separate models were created for both Al_2_O_3_–water nanofluids and ZnO-water nanofluids given in equations 36 and 37 respectively.

(36) $$\mu_{nf}=\mu_{bf}e^{\left(4.91\phi /0.2092-\phi \right)}$$

(37) $$\mu_{nf}=\mu_{bf}\left(1+46.801\phi +550.82\phi^{2}\right)$$

The viscosity of the base fluid varies with temperature. These models are valid for volume concentrations of 0–6% and 0–3% for the Al_2_O_3_ and ZnO models respectively.

Khanafer and Vafai [98] used experimental data from Nguyen et al. [12], Pak and Cho [99], Putra et al. [100], Anoop et al. [101], to develop three equations for the viscosity of a nanofluid using a least-square regression analysis. It was believed that the effect of temperature was not properly considered in previous models, therefore the models developed considered both temperature and volume fraction. The data these models were based on was 36 and 47 nm Al_2_O_3_ and several sizes of TiO_2_ and CuO, each nanofluid had water as a base liquid. The expression for the viscosity of Al_2_O_3_–water nanofluids based on curve fitting from experimental data [12,99,100,101], with viscosity as a function of volume fraction, temperature, and nanoparticle size is given in Equation (38).

(38) $$\mu_{nf}=-0.4491+\frac{28.4312}{T}+0.574\phi -0.1634\phi^{2}+\frac{23.053\phi^{2}}{T^{2}}+0.0132\phi^{3}-\frac{2354.735\phi }{T^{3}}+\frac{23.498\phi^{2}}{d_{p}{}^{2}}-\frac{3.0185\phi^{3}}{d_{p}{}^{2}}$$

The range of validity for this model is volume fraction from 1–9% and temperatures from 20–70 °C.

Abu-Nada [102] agreed with Khanafer and Vafai [98] that the effect of temperature was not properly considered in the models to determine the viscosity of a nanofluid. Abu-Nada proposed a model where the viscosity is a function of both the temperature and volume fraction, with consideration given to the data and correlations proposed by Nguyen et al. [12]. A new model was proposed with a maximum error 5% and an R^2^ value of 99.8% when compared to the data from Nguyen et al. [12]. The model proposed by Abu-Nada is given in Equation (39).

(39) $$\mu_{nf}=-0.155-\frac{19.582}{T}+0.794\phi +\frac{2094.47}{T^{2}}-0.192\phi^{2}-8.11\frac{\phi }{T}-\frac{27463.863}{T^{3}}+1.6044\frac{\phi^{2}}{T}+2.175\frac{\phi }{T^{2}}$$

The levels of accuracy of these models will be determined by comparing them to several sets of experimental data from a variety of sources. This will also demonstrate the correlation between viscosity and temperature, size and concentration.

## 4. Theoretical Modeling of Viscosity and Thermal Conductivity of Nanofluids

This section will investigate the implications of different variables including temperature, concentration, and size of particles in nanofluids. The data presented is third party experimental data reinforced with theoretical correlations that were presented in the previous section.

**Figure 39:**
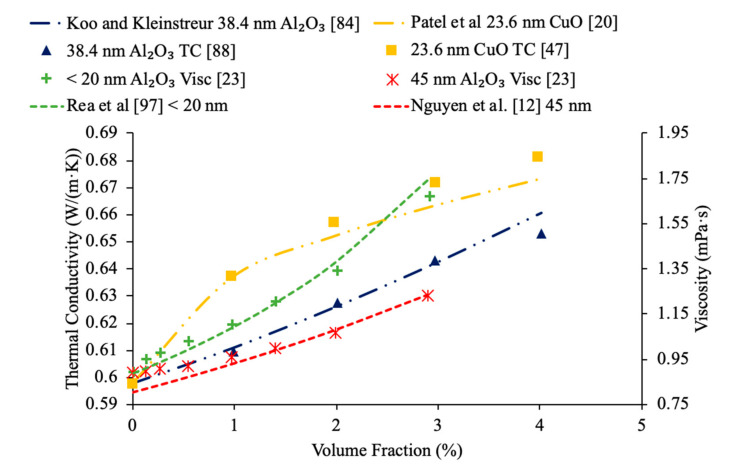
Combined figure showing nanofluid thermal conductivity (TC) and viscosity (Visc) dependence on volume fraction for various sizes of CuO–water and Al_2_O_3_–water nanofluids in comparison to different theoretical models [12,25,84,97].

The experimental data collected by Das et al. [47] for 38.4 nm Al_2_O_3_–water nanofluid and Lee et al. [88] 23.6 nm CuO–water nanofluid is compared Brownian motion models introduced by Patel et al. [20] and Koo and Kleinstreur [84]. These models are chosen since the characteristics of the nanofluids are within the range of validity for the models. The 23.6 nm CuO–water data is predicted with an average absolute error of 0.68% by the Patel et al. [20] model and the 38.4 nm Al_2_O_3_–water data is predicted with an average absolute error of 0.37% by the Koo and Kleinstreur [84] model. Additionally, viscosity data collected by Pastoriza-Gallego et al. (23) for <20 nm Al_2_O_3_–water and 45 nm Al_2_O_3_–water nanofluids is compared to models introduced by Rea et al. [97] and Nguyen et al. [12]. The models are able to accurately predict the data with the Rea et al. [97] model having an average absolute error of 2.49% for the <20 nm Al_2_O_3_–water data, while the Nguyen et al. [12] model had an average absolute error of 1.28% for the 45 nm Al_2_O_3_–water data. In Figure 39, it demonstrates the effect of concentration on both thermal conductivity and viscosity. It can be seen that as the concentration increases both the thermal conductivity and viscosity increase. The increased thermal conductivity with increased concentration can be beneficial for heat management, but the increase in viscosity with increased concentration can increase the required pumping power for the system. As will be seen in later figures, the negative consequences of increased viscosity with increased concentration can be reduced with an increase in the temperature of the nanofluid.

**Figure 40:**
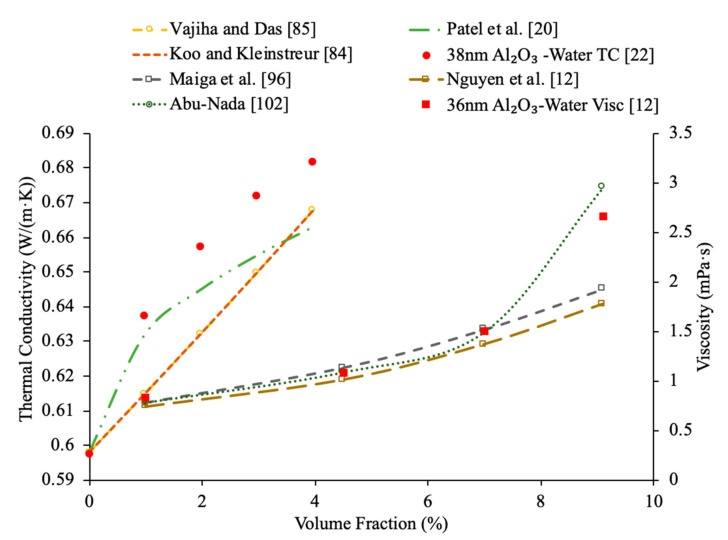
Thermal conductivity (TC) and viscosity (Visc) as a function of volume fraction for 36 and 38 nm Al_2_O_3_–water nanofluids [12,22] in comparison to various theoretical models [12,20,84,85,96,102].

Similarly, Figure 40 shows the same experimental and theoretical trend for thermal conductivity and viscosity as a function of concentration. In both cases of thermal conductivity and viscosity as the concentration increases the viscosity and thermal conductivity consequently increase. While the increased thermal conductivity is an attractive property the increasing viscosity could be detrimental to the pumping system. Requiring a more powerful pump to disperse the working fluid throughout the system.

**Figure 41:**
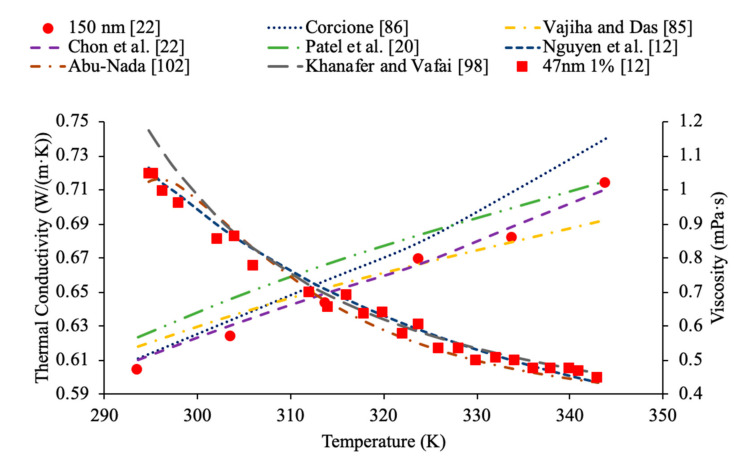
Thermal conductivity (increasing with temperature) and viscosity (decreasing with temperature) for 150 and 47 nm Al_2_O_3_–water nanofluids [12,24] in comparison to various theoretical models [12,20,22,85,86,98,102].

Figure 41 demonstrates the effect of temperature on both thermal conductivity and viscosity through the use of both experimental data and theoretical models. The 150 nm Al_2_O_3_–water at a volume fraction of 1% data from Chon et al. [22] is used to demonstrate the effect of temperature on thermal conductivity. It can be clearly seen that as the temperature increases the thermal conductivity simultaneously increases as well. The same trend between temperature and thermal conductivity is also reflected in the theoretical models introduced by Corcione [86], Vajiha and Das [85], Chon et al. [22] and Patel et al. [20]. These models all consider the effect of Brownian motion of the nanoparticles and are able to predict the thermal conductivity with an average percent error of 8% or less. The correlation between viscosity and temperature can also be seen in Figure 41, where the increase in temperature causes a decrease in viscosity. 47 nm Al_2_O_3_–water at a volume fraction of 1% data from Nguyen et al. [12] is compared to theoretical models introduced by Nguyen et al. [12], Abu-Nada [102] and Khanafer and Vafai [98]. The theoretical models all accurately follow the experimental data with average absolute errors of 3.17%, 4.47%, and 2.10% for the Nguyen et al. [12], Abu-Nada [102] and Khanafer and Vafai [98] models, respectively.

**Figure 42:**
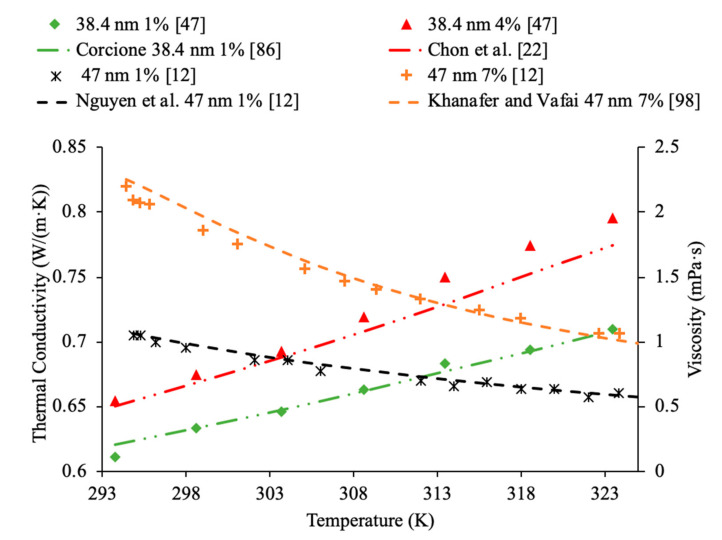
Thermal conductivity (increasing with temperature) and viscosity (decreasing with temperature) for 38.4 nm Al_2_O_3_–Water nanofluid at concentrations of 1% and 4% [47] and for 47 nm Al_2_O_3_–water nanofluid at concentrations of 1% and 7% [12] in comparison to different theoretical models [12,22,86,98].

In Figure 42, thermal conductivity data for 38.4 nm Al_2_O_3_–water nanofluids at a volume fraction of 1% and 4% and viscosity for 47 nm Al_2_O_3_–water nanofluids at a volume fraction of 1% and 7% from Das et al. [47] and Nguyen et al. [12], respectively, demonstrate the correlation with concentration and temperature. Previously Figure 41 demonstrated the increase in viscosity caused by an increase in the concentration of the nanofluid. In Figure 42, however, it can be seen that while at low temperatures the viscosity of the 47 nm Al_2_O_3_–water nanofluid at a volume fraction of 7% is significantly higher than the 47 nm Al_2_O_3_–water nanofluid at a volume fraction of 1%, at higher temperatures the difference in viscosity decreases significantly. Therefore, by increasing the temperature of the nanofluid the concentration can be increased as well to further enhance the thermal conductivity, without as significant of an increase in viscosity. Allowing for the properties of the nanofluid to be optimized by maximizing the increase in thermal conductivity, while limiting the increase in viscosity. The experimental data also demonstrate very close agreement with the theoretical models introduced by Nguyen et al. [12], Chon et al. [22], Corcione [86] and Khanafer and Vafai [98].

**Figure 43:**
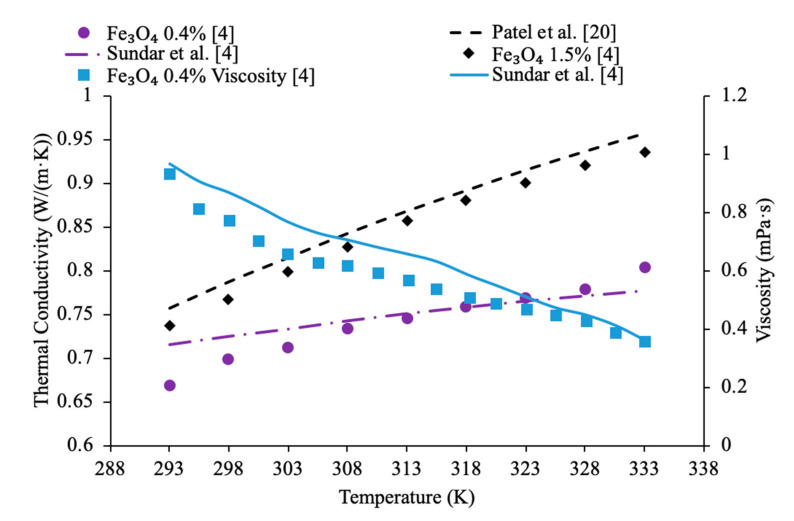
Thermal conductivity (increasing with temperature) and viscosity (decreasing with temperature) for 13 nm Fe_3_O_4_–water nanofluids at 0.4% and 1.5% [4] in comparison to various theoretical models [4,20].

Similar to Figure 42 and Figure 43 shows viscosity and thermal conductivity as a function of temperature. Data is taken from Sundar et al. [4] of a Fe_3_O_4_–water nanofluid consisting of 13 nm particles at concentrations of 0.4% and 1.5%. Similar trends are observed even with this iron-oxide particle. As the temperature increases, the viscosity tends to decrease as the particles continue to get energized allowing the fluid to flow easier. The thermal conductivity, on the other hand, further increases as the temperature increases. Theoretical correlations from Patel et al. [20] and Sundar et al. [4] are in good agreement with the experimental trends and can be observed in Figure 43.

**Figure 44:**
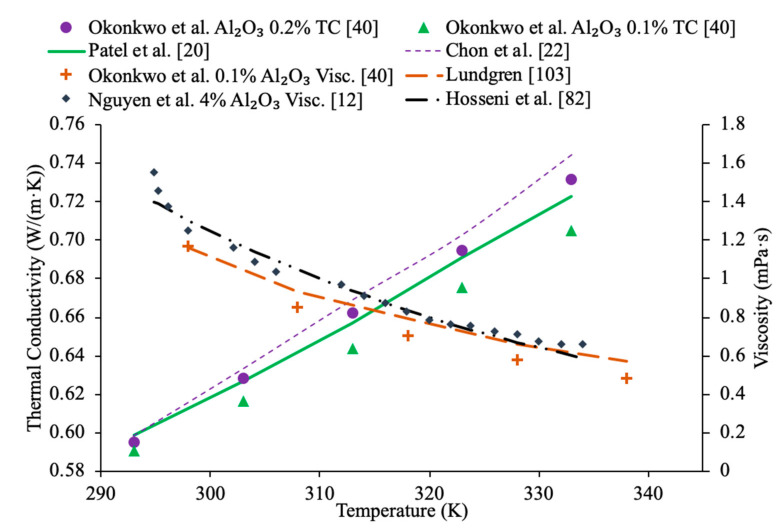
Thermal conductivity (increasing with temperature) and viscosity (decreasing with temperature) for Al_2_O_3_–water nanofluid at 0.1%, 0.2% [70] (40) and 4% concentrations [12] in comparison to various theoretical models [20,22,82,103].

Figure 44 shows the effect of thermal conductivity and viscosity with respect to temperature for Al_2_O_3_–water nanofluids. Specifically, Figure 44 shows data from Okonkwo et al. [40] and Nguyen et al. [12] at concentrations of 0.1%, 0.2% and 4%. An observation can be made when looking at the graph that the increased concentration increases both viscosity and thermal conductivity. Observing the viscosity data, the 4% concentration has a greater viscosity at room temperature than the 0.1%. Similarly looking at the gathered thermal conductivity data, the 0.2% concentration has a greater room temperature and end temperature value than that of the 0.1% concentration. On the other hand, as the temperature increases, the viscosity decreases and the thermal conductivity increases. Theoretical equations from Patel et al. [20], Chon et al. [22], Lundgren [103] and Hosseni et al. [82] are used to approximate the values of thermal conductivity and viscosity with changing temperature and are in close agreement with the experimental data, further reinforcing the proposed trends.

**Figure 45:**
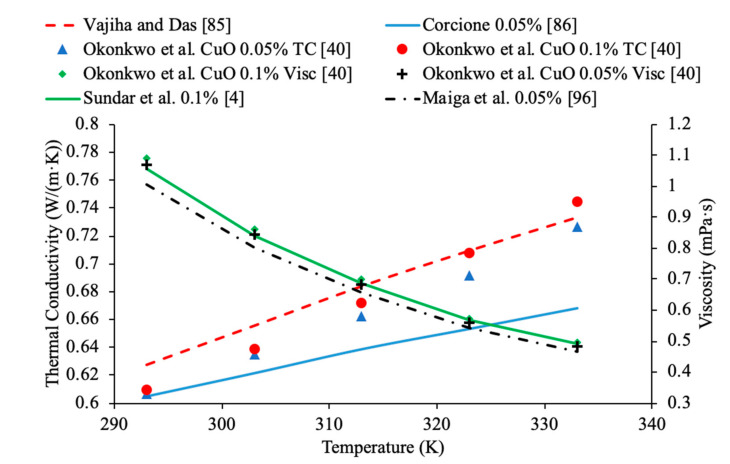
Thermal conductivity (increasing with temperature) and viscosity (decreasing with temperature) for CuO–water nanofluids at 0.1% and 0.05% [70] (40) concentrations in comparison to different theoretical models [4,85,86,96].

Lastly, Figure 45 shows the thermal conductivity and viscosity for CuO–water nanofluid data gathered from Okonkwo et al. [40] at concentrations of 0.05% and 0.1%. The same trend is apparent regardless of material of particles. Again, as the temperature increases the thermal conductivity is further enhanced while the viscosity decreases significantly. Vajiha and Das [85], Corcione [86], Sundar et al. [4] and Maiga et al. [96] theoretical models are utilized to approximate the values of the experimental data. The models closely approximate the values with absolute average errors of less than 4%.

Both the thermal conductivity and viscosity are important parameters to consider in the use of a nanofluid in any of the applicable fields for nanofluid use. Electronic is one field where the ability of the working fluid to remove a sufficient amount of heat to maintain an appropriate temperature is vital, but it is necessary to be able to pump the fluid as well. This demand for efficient cooling makes the increased thermal conductivity of nanofluids a very attractive property. However, the increased viscosity of nanofluids, especially at low temperature, and the accompanying increase in pumping power needed limits the application of nanofluids. Both theoretical and experimental results demonstrate that as the temperature of nanofluids increases the thermal conductivity increases, while the viscosity decreases reducing the need for concern over pumping power. Frequently the operating temperature of the working fluid electronics is between 37–72 °C. In this temperature range the viscosity of the nanofluid is significantly reduced compared to room temperature, while the thermal conductivity is enhanced. Additional details for the theoretical data analyzed within this review paper are shown below in Table A2 in Appendix A.

## 5. Plan of Future Work

Furthermore, future work in this field would include a greater focus on studying the effects of the various parameters on nanofluid thermal conductivity and viscosity in combination. Since the parameters affect one another more focus is needed on studying the parameters in combination rather than individually. Additionally, since the optimization of nanofluids requires both maximizing thermal conductivity and minimizing viscosity, more work is required to study these two nanofluid properties, simultaneously. The effect of the parameters discussed must be determined for both thermal conductivity and viscosity. Of the parameters reviewed in this paper, the effect of nanoparticle size and nanoparticle shape posed the greatest challenge to determine their effect on the nanofluid thermal conductivity and viscosity. As a result, these parameters may require additional focus. Finally, since there are not yet conclusive models for either the thermal conductivity or viscosity of a nanofluid, more comparisons between the experimental data and the theoretical models are needed. These comparisons will allow for greater understanding of the limitations of the models so they can be improved to better predict the nanofluid thermal conductivity and viscosity.

## 6. Conclusions

This paper consisted of combining the effects of concentration, various surfactants, temperature, base liquid, and nanoparticle characteristics such as size, shape, and material on the thermal conductivity and viscosity of nanofluids. This paper investigated how to optimize the effects of nanoparticles in nanofluids by achieving the maximum thermal conductivity, while minimizing the viscosity. The results were limited to the given data that was analyzed. Therefore, the results may have been different if further data was analyzed.

This investigation found:

- An increase in nanoparticle concentration led to an increase in thermal conductivity, which is due to increase in Brownian motion, thermophoresis of particles, and particle collisions. Brownian motion creates microconvection in the surrounding liquid molecules helping to increase the thermal conductivity. Thermophoresis causes particles to collide more, which increases heat transfer. Similarly, an increase in nanoparticle concentration led to an increase in viscosity due to an increase in interaction between nanoparticle-nanoparticle and nanoparticle-molecule of base liquid.
- An increase in nanoparticle size, can increase or decrease thermal conductivity, but in a majority of studies the smaller nanoparticles had a higher thermal conductivity. Additionally, an increase in nanoparticle size decreases the viscosity of the nanofluid, but as the nanoparticles become too large it becomes unstable limiting the maximum size of nanoparticles. As the nanoparticle size increases there is a decrease in thermal conductivity due to a decrease in Brownian motion and a lower surface area to volume ratio. Enhancing Brownian motion creates more paths for heat transfer, helping to increase the thermal conductivity. The bigger particles have a lower surface area to volume ratio, which decreases thermal conductivity as heat transfer is a function of surface area. In addition, an increase in nanoparticle size decreases the viscosity of the nanofluid due to the forming of less agglomerates. Particle agglomeration involves the process of putting particles into close proximity to each other, which helps to increase the viscosity of the nanofluid.
- The addition of surfactants at low concentrations help to increase thermal conductivity, but at high concentrations of surfactants, they help to reduce thermal conductivity of the nanofluid. Thermal conductivity increases with the addition of surfactants due to the particles having more freedom to move. A surfactant can negatively charge the nanoparticles causing them to repel each other leading to more movement, which leads to more particle collisions to transfer energy and decrease the nanofluid viscosity.
- As temperature increases, thermal conductivity increases and viscosity decreases. Thermal conductivity of nanofluids increases with temperature due to the increase in Brownian motion and an increase in kinetic energy of particles. An increase in Brownian motion allows for more convection, leading to a higher thermal conductivity. An increase in kinetic energy of particles, means more particles colliding, which increases heat transfer. In addition, as temperature rises the viscosity of a nanofluid decreases due to weakening of intermolecular forces between nanoparticles. When weakening the intermolecular forces, the particles, it causes the nanofluid to become less stable, decreasing the viscosity of the nanofluid.
- Water was one of the main base liquids studied and it had the highest thermal conductivity and lowest viscosity. The addition of the base liquids of ethylene glycol and propylene glycol to the water reduced the thermal conductivity and increased viscosity of the nanofluid. This is due to the fact that ethylene glycol and propylene glycol have a lower thermal conductivity than water, so when more ethylene glycol and propylene glycol is added to the water the overall thermal conductivity of the nanofluid decreases. Ethylene glycol and propylene glycol have a viscosity higher than water, so when they are added to water, the overall viscosity of the nanofluid increases.
- The effects of nanoparticle shape on thermal conductivity and viscosity need to be investigated further, but cubic shaped nanoparticles within a nanofluid had a higher thermal conductivity than a rod or spherical shaped nanoparticles within a nanofluid. The cubic shaped nanoparticles have a higher surface area to volume ratio when compared to the rod and spherical shaped nanoparticles, but this may change depending on the radius and height of the nanoparticles used. A higher surface area to volume ratio means higher heat transfer as heat transfer is a function of surface area. Additionally, the effect of the shape of the nanoparticles on the viscosity of a nanofluid was studied. There was not much of a change in the viscosity of a nanofluid when changing the shape of the nanoparticles.
- The long single wall carbon nanotubes (L-SWCNT) had a higher thermal conductivity than the short single wall carbon nanotubes (S-SWCNT) and multiwall carbon nanotubes (MWCNT) because L-SWCNT particles having a higher aspect ratio than the S-SWCNT and MWCNT, which creates more contact between the base fluid, leading to higher heat transfer abilities. Additionally, the effect of material of nanoparticles on the viscosity of a nanofluid was studied. It has been found that the material of nanoparticles does play a role on the viscous properties of a nanofluid.
- For some hybrid nanofluids the thermal conductivity and viscosity increased when comparing it to the individual nanofluids. Therefore, hybrid nanofluids have better properties when it comes to its thermal conductivity, but not always when it comes to viscosity. Sometimes a hybrid nanofluid can have a lower thermal conductivity than the individual nanofluids on their own, and have a higher viscosity than both, but most hybrid nanofluids studied had a higher thermal conductivity than the nanofluids themselves, and a higher viscosity than the nanofluids. Moreover, further investigation is needed in order to understand the effects of second particles or engineer the nanofluid to have the most ideal effects on thermal conductivity and viscosity.
- Lastly, based on the data analyzed the Brownian models closely matched the experimental data. This may not hold true if additional data was analyzed. Therefore, there is not a clear adequate model for the thermal conductivity of nanofluids. Additionally, an adequate model for determining the viscosity of a nanofluid could not be determined as the accuracy of the models varied based on the experimental data.

## Figures and Tables

**Table 1:** Empirical constants for different types of nanoparticles at ϕ ≥ 1% and a temperature range of 300 K ≤ T ≤ 325 K.

Type of Particle	ζ	Concentration	Temperature
${\mathrm{Al}}_{2}O_{3}$	$0.0011{\left(100\phi \right)}^{-0.7272}$	ϕ≥ 1%	300 K ≤ T ≤ 325 K
CuO	$0.0017{\left(100\phi \right)}^{-0.0841}$	ϕ≥ 1%	300 K ≤ T ≤ 325 K

**Table 2:** Empirical constants for different types of nanoparticles and concentrations with a temperature range of 298 K ≤ T ≤ 363 K.

Type of Particle	ζ	Concentration	Temperature
${\mathrm{Al}}_{2}O_{3}$	$8.4407{\left(100\phi \right)}^{-1.07304}$	1% ≤ ϕ ≤ 10%	298 K ≤ T ≤ 363 K
CuO	$9.8810{\left(100\phi \right)}^{-0.9446}$	1% ≤ ϕ ≤ 6%	298 K ≤ T ≤363 K
ZnO	$8.4407{\left(100\phi \right)}^{-1.07304}$	1% ≤ ϕ ≤ 7%	298 K ≤ T ≤ 363 K

## Data Availability

Data sharing is not applicable. No new data were created or analyzed in the study. Data sharing is not applicable to this article.

## Nomenclature

Nomenclature for existing theoretical modeling investigations.

k	Thermal conductivity of nanofluid ($W/\left(m\cdot K\right))$
v	Kinematic viscosity (m^2^/s)
$k_{b}$	Boltzmann Constant (-)
T	Temperature (K)
A	Empirical constant (-)
Re	Reynolds number (-)
Pr	Prandtl number of base fluids (-)
M	Empirical constant (-)
$T_{0}$	Reference temperature (K)
$c_{p}$	Specific heat (J/kg·K)
$R_{b}$	Thermal boundary resistance ($\left(m^{2}\cdot K)/W\right)$
r	Radius (m)
n	Empirical shape factor (-)
Bi	Biot number (-)
d	Diameter (m)
$l_{bf}$	Mean free path (nm)
**Greek Symbols**	
ϕ	Volume fraction of nanoparticle in base fluid(-)
$\delta_{nl}$	Nanolayer thickness (-)
$\beta_{nl}$	Dimensionless nanolayer parameter (-)
β	Empirical correlation (-)
γ	Ratio of nanolayer thickness to nanoparticle radius (-)
ρ	Density ($\mathrm{kg}/m^{3})$
ζ	Empirical constant (-)
α	Thermal diffusivity ($m^{2}/s)$
μ	Dynamic viscosity (mPa·s)
**Subscripts**	
p	Particle
bf	Base fluid
m	Matrix
nf	Nanofluid

## Appendix A

**Table A1:** Summary of existing experimental investigations.

Ref.	Specifications	Remarks
Yaganeh et al. [3] (2010)	Nanoparticle Characteristics: Nano diamond, 10 nm Base liquid: Water Range of concentration: 0.8–3% vol Range of temperature: 30–50 °C	Addition of nanoparticles increases thermal conductivity Thermal conductivity increases with increase in volume fraction nonlinearly Thermal conductivity increases with temperature Classical models underestimate thermal conductivity
Sundar et al. [4] (2013)	Nanoparticle Characteristics: Magnetic Fe_3_O_4_, 13 nm, cubic Base liquid: Water Possible surfactant: CTAB Range of concentration: 0–2% vol Range of temperature: 20–60 °C	Thermal conductivity and viscosity increase with increase in concentration Thermal conductivity increases with increase in temperature Viscosity decreases with increase in temperature Brownian motion increases thermal conductivity Fluids have Newtonian behavior
Gao et al. [5] (2020)	Nanoparticle Characteristics: Fe_3_O_4_, 12–25 nm, spherical Base liquid: Water Possible surfactant: PEG-4000 Range of concentration: 0.05–2% vol Range of temperature: 10–65 °C	Thermal conductivity and viscosity increase with an increase in concentration Thermal conductivity increases with increase in temperature Viscosity decreases with an increase in temperature
Afrand et al. [6] (2016)	Nanoparticle Characteristics: Magnetic Fe_3_O_4_, 20–30 nm, spherical Base liquid: Water Range of concentration: 0.1–3% vol Range of temperature: 20–55 °C	Thermal conductivity increases with increase in temperature and concentration
Godson et al. [7] (2010)	Nanoparticle Characteristics: Ag, 60 nm Base liquid: Water Range of concentration: 0.3–0.9% vol Range of temperature: 50–90 °C	Thermal conductivity increases with an increase in concentration and temperature. Viscosity increases with an increase in concentration and decreases with an increase in temperature. Brownian motion increases with an increase in temperature. Existing models under predict thermal conductivity and viscosity
Li et al. [8] (2006)	Nanoparticle Characteristics: CuO-29 nm, non-spherical Al_2_O_3_-36 nm, spherical Base liquid: Water Range of concentration: 2–10% vol Range of temperature: 27.5–34.7 °C	Thermal conductivity increases with increase in temperature and concentration. Thermal conductivity is more a function of concentration than temperature. Maxwell model under predicts thermal conductivity.
Pryazhnikov et al. [9] (2017)	Nanoparticle Characteristics: SiO_2_, Al_2_O_3_, TiO_2_, ZrO_2_, CuO, 10–150 nm Base liquid: Water, ethylene glycol, engine oil Possible surfactant: Acrylic polymer Range of concentration: 0.25–8% vol Range of temperature: Room temperature	Classical theories under predict thermal conductivity. Material does have a significant effect on thermal conductivity Base liquid significantly impacts thermal conductivity
Xie et al. [10] (2010)	Nanoparticle Characteristics: MgO, TiO_2_, ZnO, Al_2_O_3_, and SiO_2_, 20 nm Base liquid: Ethylene glycol Possible surfactant: SDS Range of concentration: 0.5–5% vol Range of temperature: 10–60 °C	MgO nanofluids had the highest thermal conductivity and lowest viscosity. Thermal conductivity increases as temperature increases. Models under predict thermal conductivity and viscosity. Nanofluid thermal conductivity corresponds to base fluid thermal conductivity.
Yu et al. [11] (2009)	Nanoparticle Characteristics: ZnO Base liquid: Ethylene Glycol Range of concentration: 5% vol Range of temperature: 10–60 °C	Thermal conductivity increases non-linearly with concentration increasing Volume of 5% had a 26.5% increase in thermal conductivity compared to base liquid
Nguyen et al. [12] (2007)	Nanoparticle Characteristics: Al_2_O_3_-36 nm, 47 nm CuO-29 nm Base liquid: water Range of concentration: 1–12% vol Range of temperature: 22–75 °C	Viscosity increases with increase in concentration Viscosity decreases with increase in temperature Particle size has more effect at high concentrations CuO has higher viscosity Classic models not accurate at predicting viscosity
Yiamsawas et al. [13] (2013)	Nanoparticle Characteristics: Al_2_O_3_-120 nm, TiO_2_-21 nm Base liquid: water Range of concentration: 1–8% vol Range of temperature: 15–60 °C	Viscosity decreases as temperature increases Viscosity increases as concentration Al_2_O_3_ nanofluid has higher viscosity than TiO_2_ nanofluid Larger size of Al_2_O_3_ nanoparticles thought to be the cause if the higher viscosity Classical models do not accurately model viscosity
Kole et al. [14] (2010)	Nanoparticle Characteristics: Al_2_O_3_ < 50 nm Base liquid: Engine Coolant Possible surfactant: Oleic Acid Range of concentration: 0.1–1.5% vol Range of temperature: 10–50 °C	Nanofluids are non-Newtonian Viscosity increases with increased concentration Viscosity decreases with increased temperature Classical models underpredict viscosity Models considering Brownian motion are more accurate
Malekzadeh et al. [15] (2016)	Nanoparticle Characteristics: Magnetic Fe_3_O_4_-20–30 nm Base liquid: water Range of concentration: 0–1% vol Range of temperature: 25–45 °C	Viscosity increases with an increase in concentration Viscosity decrease with an increase in concentration Magnetic field increases viscosity Increased concentration and magnetic field caused chain like structures to form which increased viscosity
Yu et al. [16] (2011)	Nanoparticle Characteristics: AlN,165 nm Base liquid: Propylene Glycol, Ethylene Glycol Range of concentration: 5–10% vol Range of temperature: 10–60 °C	Thermal conductivity of nanofluid follows same trend as base fluid Nanofluids have Newtonian behavior below 5% Nanofluids demonstrate shear-shinning behavior above 5%
Sundar et al. [17] (2016)	Nanoparticle Characteristics: ND Base liquid: Water Range of concentration: 0.2–1% vol Range of temperature: 20–60 °C	Thermal conductivity increased with an increase in concentration Viscosity increased with an increase in concentration
Pastoriza-Gallego et al. [18] (2011)	Nanoparticle Characteristics: Al_2_O_3_ Base liquid: Water–ethylene glycol mixture Range of concentration: 1.5–8.6% vol (thermal conductivity) 0.5–6.6% vol (viscosity) Range of temperature: 10–50 °C	Thermal conductivity increased with an increase in concentration Viscosity increased with an increase in concentration
Sundar et al. [19] (2014)	Nanoparticle Characteristics: Al_2_O_3_ 30 nm Base liquid: Water–ethylene glycol mixture Range of concentration: 0.3–1.5% vol Range of temperature: 20–60 °C	Thermal conductivity increased with an increase in temperature and concentration Viscosity decreases with an increase in temperature Viscosity increase with an increase in concentration Classical models fail to predict thermal conductivity and viscosity
Patel et al. [20] (2009)	Nanoparticle Characteristics: Al_2_O_3_ 11–150 nm Base liquid: Ethylene glycol Range of concentration: 1–3% vol Range of temperature: 20–50 °C	Decrease in thermal conductivity with an increase in particle size Decrease in thermal conductivity caused by decrease in Brownian motion and increase in surface area to volume ratio Thermal conductivity increased and viscosity decreased with temperature increasing
Gangadevi et al. [21] (2018)	Nanoparticle Characteristics: Al_2_O_3_, CuO and Al_2_O_3_–CuO hybrid nanofluids Base liquid: Water Range of concentration: 0.2% vol Range of temperature: 20–60 °C	Thermal conductivity increased and viscosity decreased with temperature increasing
Chon et al. [22] (2005)	Nanoparticle Characteristics: Al_2_O_3_ 11–150 nm Base liquid: Water Range of concentration: 2% vol Range of temperature: 20–70 °C	There was a decrease in thermal conductivity with an increase in nanoparticle size Decreased in thermal conductivity explained by Brownian velocity Larger particles mean lower Brownian velocity which leads to lower energy transfer
Kwek et al. [24] (2010)	Nanoparticle Characteristics: Al_2_O_3_ 10–150 nm Base liquid: Water Range of concentration: 5% vol Range of temperature: 15–60 °C	Initial decline in thermal conductivity as diameter (size) of nanoparticles increased At 35 nm the thermal conductivity started to increase as diameter (size) of nanoparticles increased Decrease in thermal conductivity thought to be due to Brownian motion Increase in thermal conductivity thought to be due to diffusive heat transfer
Beck et al. [25] (2008)	Nanoparticle Characteristics: Al_2_O_3_ 8–282 nm Base liquid: Water Range of concentration: 4% vol Range of temperature: Room temperature	Initial decline in thermal conductivity as diameter (size) of nanoparticles increased Then, thermal conductivity increases with particle size, but no clear relationship Increase in thermal conductivity thought to be due to particle aggregation
Rudyak et al. [26] (2018)	Nanoparticle Characteristics: SiO_2_, Al_2_O_3_, and TiO_2_ 10–150 nm Base liquid: Water Range of concentration: 2% vol Range of temperature: 25–60 °C	All nanofluids tested increased in thermal conductivity with particle size increasing No decrease was shown with thermal conductivity when particle size increased Classical models to not predict thermal conductivity and viscosity Increase in particle size decreases viscosity
Kim et al. [28] (2006)	Nanoparticle Characteristics: ZnO-10–60 nm, TiO_2_-10–70 nm, Al_2_O_3_-38 nm Base liquid: Water, EG Possible surfactant: SDS Range of concentration: 0.3–3% vol Range of temperature: Constant 20 °C	All nanofluids decreased in thermal conductivity with an increase in nanoparticle size Not a huge decrease in thermal conductivity as size increased suggesting that particle size may not play a significant role in the thermal conductivity properties of a nanofluid Decrease in thermal conductivity thought to be due to a decrease in Brownian motion
Murshed et al. [31] (2008)	Nanoparticle Characteristics: Al_2_O_3_ 80 nm Base liquid: Ethylene glycol Range of concentration: 1% vol Range of temperature: 20–50 °C	Thermal conductivity increases as temperature increases
Esfe et al. [32] (2015)	Nanoparticle Characteristics: Al_2_O_3_ Base liquid: Ethylene glycol (EG) Range of concentration: 0.2–5% vol Range of temperature: 24–50 °C	Thermal conductivity increases with nanoparticle concentration
Omrani et al. [33] (2019)	Nanoparticle Characteristics: Carbon nanotubes (MWCNT) 8–50 nm Base liquid: Water Possible surfactant: SDBS and Tr-X Range of concentration: 0.05% vol Range of temperature: 10–45 °C	All nanofluids increased in thermal conductivity with an increase in nanoparticle size Largest increase found in nanofluids containing MWCNTs just above 8 nm Smallest increase found in nanofluids containing nanoparticles just below 50 nm Increase of aspect ratio increases thermal conductivity Nanofluids have Newtonian behavior
Turgut et al. [34] (2009)	Nanoparticle Characteristics: TiO_2_, 21 nm Base liquid: Water Range of concentration: 0.2–3% vol Range of temperature: 13–55 °C	Thermal conductivity increases with an increase in concentration Viscosity increases with an increase in concentration Viscosity enhancement is greater than predicted by Einstein model
Jia-Fei et al. [35] (2009)	Nanoparticle Characteristics: SiO_2_ 7–40 nm Base liquid: Water Range of concentration: 0.1–2% vol Range of temperature: Constant 25 °C	Found that viscosity decreased with an increase in nanoparticle size Decrease in viscosity thought to be due to the difference in the aggregates formed in the nanofluids
Abdul Hamid et al. [37] (2015)	Nanoparticle Characteristics: Al_2_O_3_ Base liquid: 40% ethylene glycol-60% water Range of concentration: 0.5–2% vol Range of temperature: 30–70 °C	Decrease in viscosity with an increase in temperature
Bidgoli et al. [38] (2016)	Nanoparticle Characteristics: CuO, 40 nm Base liquid: Water-EG Range of concentration: 0.1–2% vol Range of temperature: 20–60 °C	Viscosity increases with increasing concentration of nanoparticles Viscosity decreases with increasing temperature
Okonkwo et al. [40] (2020)	Nanoparticle Characteristics: Al_2_O_3_ 29.2 nm Base liquid: Water Range of concentration: 0.2% vol Range of temperature: 25–65 °C	As temperature increases the thermal conductivity increased while the viscosity of the nanofluid decreased for nanoparticles of 29.2 nm
Khairul et al. [43] (2016)	Nanoparticle Characteristics: CuO and Al_2_O_3_ Base liquid: Water Possible surfactant: SDBS Range of concentration: 0.05–0.15 wt Range of temperature: Constant 25 °C	The SDBS surfactant caused the nanoparticles to repel each other creating a more stable nanofluid Thermal conductivity increased with the addition of the surfactant and reached a maximum value
Das et al. [44] (2018)	Nanoparticle Characteristics: TiO_2_ Base liquid: Water Possible surfactant: CTAB and SDS Range of concentration: 0.1–1% vol Range of temperature: 20–60 °C	Thermal conductivity was higher at all concentrations and temperatures for the TiO_2_ with the SDS surfactant than the TiO_2_ with the CTAB surfactant
Freitas et al. [45] (2020)	Nanoparticle Characteristics: MWCNT Base liquid: Water Possible surfactant: AG, TrX, and COOH Range of concentration: 0.5–1% vol Range of temperature: 30–60 °C	Thermal conductivity increased with an increase in temperature and weight fraction Highest thermal conductivity was found in the MWCNT with the COOH as a surfactant
Das et al. [46] (2017)	Nanoparticle characteristics: Al_2_O_3_, 20–70 nm, rod shaped Base liquid: water Possible surfactant: SDS, SDBS, CTAB Range of concentration: 0.1–2% vol Range of temperature: 20–60 °C	SDBS offers the best stabilization SDBS reduces particle clustering Thermal conductivity increases with increase in temperature and concentration Viscosity increases with an increase in concentration Viscosity decreases with an increase in temperature
Das et al. [47] (2003)	Nanoparticle Characteristics: Al_2_O_3_ Base liquid: Water Range of concentration: 1–4% vol Range of temperature: 21–51 °C	Thermal conductivity increased with an increase in temperature Increase in thermal conductivity thought to be due to an increase in Brownian motion
Esfe et al. [48] (2015)	Nanoparticle Characteristics: Al_2_O_3_, 5 nm, spherical Base liquid: Water Range of concentration: 0.25–5% vol Range of temperature: 26–55 °C	Thermal conductivity increases as temperature and concentration increase
Krishnakumar et al. [49] (2018)	Nanoparticle Characteristics: Al_2_O_3_ Base liquid: Ethylene glycol Range of concentration: 0–1% vol Range of temperature: 25–50 °C	Each of the volume fractions tested an increase in thermal conductivity was found with an increase in temperature Increase in thermal conductivity was nonlinear Percent increase was higher for the nanofluid than for the base liquid
Maheshwary et al. [50] (2017)	Nanoparticle Characteristics: TiO_2_ Base liquid: Water Range of concentration: 0.5–2.5 wt % Range of temperature: 30–80 °C	Each weight percent tested an increase in thermal conductivity was found with an increase in temperature
Shima et al. [51] (2010)	Nanoparticle Characteristics: Fe_3_O_4_ Base liquid: Water Range of concentration: 1.02% vol Range of temperature: 25–50 °C	Thermal conductivity increased with temperature increasing Increase in thermal conductivity attributed to an increase in Brownian motion
Esfe et al. [52] (2015)	Nanoparticle Characteristics: Mg (OH)_2_ Base liquid: Ethylene glycol Range of concentration: 0.1–2% vol Range of temperature: 24–65 °C	Viscosity decreased with an increase in temperature Higher concentration of nanoparticles experienced a greater decrease in viscosity with temperature increasing
Sundar et al. [53] (2013)	Nanoparticle Characteristics: Fe_3_O_4_ Base liquid: Water–Ethylene glycol Range of concentration: 0.2–2% vol Range of temperature: 20–60 °C	Thermal conductivity increased with temperature increasing Increase suggested to be the case due to an increase in Brownian motion Increase in thermal conductivity highest in nanofluids with greater amount of water
Chiam et al. [54] (2017)	Nanoparticle Characteristics: Al_2_O_3_ Base liquid: 40% ethylene glycol-60% water Range of concentration: 0.2–1% vol Range of temperature:30–70 °C	Decrease in viscosity as temperature increases
Li et al. [55] (2016)	Nanoparticle Characteristics: SiC Base liquid: Engine coolant Range of concentration: 0.1–0.5% vol Range of temperature: 10–50 °C	Thermal conductivity increased with temperature increasing Viscosity decreased with temperature increasing
Urmi et al. [56] (2020)	Nanoparticle Characteristics: Hybrid made up of Al_2_O_2_-13 mm and TiO_2_-5–6 nm, 80% TiO_2_-20% Al_2_O_3_ Base liquid: Water and ethylene glycol Range of concentration: 0.02–0.1% vol Range of temperature: 30–80 °C	Al_2_O_3_–TiO_2_ hybrid nanofluid has bigger thermal conductivity than either TiO_2_ or Al_2_O_3_ nanofluid Thermal conductivity increases with temperature and nanoparticles concentration Fluids exhibit Newtonian behavior Combined effect of thermal conductivity and viscosity makes hybrid nanofluid optimal for heat transfer
Wang et al. [58] (2012)	Nanoparticle Characteristics: Graphene and MWCNT Base liquid: [HMIM]BF_4_ Range of concentration: 0.03–0.06 wt % Range of temperature: 25–65 °C	The base liquid of [HMIM]BF_4_ acts similar to other base liquid in how thermal conductivity increased with temperature and concentration of nanoparticles increasing
AL-Waeli et al. [59] (2019)	Nanoparticle Characteristics: SiC Base liquid: Water, ethylene glycol, propylene glycol Possible surfactant: CTAB Range of concentration: 0.5 wt % Range of temperature: 25–65 °C	Thermal conductivity of SiC nanofluid with changing base liquids did not change much Concluding the base liquid does not play an important role in the thermal conductivity of a nanofluid
Kumar et al. [61] (2019)	Nanoparticle Characteristics: Hybrid made of Al_2_O_3_ and CuO 50:50 Base liquid: Water, ethylene glycol, propylene glycol Possible surfactant: Range of concentration: 0–1.5% vol Range of temperature: 50–70 °C	The viscosity increased in the hybrid nanofluid with increasing mass fractions of EG and PG EG and PG have higher viscosities than water The nanofluid with EG and water had a lower viscosity than the nanofluid with PG and water EG has a lower viscosity than PG
Timofeeva et al. [62] (2011)	Nanoparticle Characteristics: SiC 16–90 nm Base liquid: Water and ethylene glycol Range of concentration: 1–4% vol Range of temperature: 15–85 °C	Relative viscosity was higher for the SiC-water nanofluid than the SiC-EG nanofluid
Esfe et al. [63] (2015)	Nanoparticle Characteristics: CuO Base liquid: Water-EG Range of concentration: 0.1–2% vol Range of temperature: 20–50 °C	As temperature and concentration thermal conductivity increases Effect of temperature is greater in higher concentrations
Main et al. [65] (2020)	Nanoparticle Characteristics: Al_2_O_3_ Base liquid: [C4mim] [NTf2] Range of concentration: 1 wt Range of temperature: 10–90 °C	Each nanofluid had a viscosity greater than the base liquid Decrease in the effect of the nanoparticles on the viscosity at high temperatures
Zhu et al. [66] (2018)	Nanoparticle Characteristics: CuO Base liquid: Dimethicone Range of concentration: 0.15–0.75% vol Range of temperature: 25–65 °C	As the concentration of nanoparticles increased at a temperature of 25 °C, the viscosity increased as well However, no clear difference between the viscosity of the two nanofluids with different shaped nanoparticles Proposed to be due to a lower volume concentration of nanoparticles.
Timofeeva et al. [67] (2009)	Nanoparticle Characteristics: AlO (OH) Platelets 9 nm, cylinders 80 × 10 nm, blades 60 × 10 nm, and bricks 40 nm. Base liquid: Water Range of concentration: 1–7% vol Range of temperature: 15–85 °C	Platelet shaped nanoparticles within a nanofluid have a higher viscosity than cylindrical, blade and bricklike shaped nanoparticles within a nanofluid
Xing et al. [68] (2015)	Nanoparticle Characteristics: Carbon nanotubes (MWCNT) with S-SWNT and L-SWNT Base liquid: Water Surfactant: CTAB Range of concentration: 0.05–0.48% vol Range of temperature: 10–60 °C	Thermal conductivity increases with an increase in concentration and temperature Highest thermal conductivity in L-SWNT followed by S-SWNT the MWNT Higher aspect ratio in carbon nanotubes leads to higher thermal conductivity
Sundar et al. [71] (2014)	Nanoparticle Characteristics: Hybrid made of multiwall carbon nanotube (MWCNT) and Fe_3_O_4_ Base liquid: Water Range of concentration: 0.1–0.3% vol Range of temperature: 20–60 °C	Thermal conductivity increases as concentration and temperature increase Viscosity decrease as temperature increase Viscosity increase as concentration increases
Lyu et al. [72] (2020)	Nanoparticle Characteristics: MWCNT, 7 nm Base liquid: water Range of concentration: 0.1–0.5% vol Range of temperature: 25–60 °C	Thermal conductivity increases as temperature and concentration increases As temperature increases viscosity decreases As concentration increases viscosity increases
Sundar et al. [73] (2016)	Nanoparticle Characteristics: Hybrid made up of Nanodiamond (ND) and Fe_3_O_4_ Base liquid: Water, Water-EG Range of concentration: 0.05–0.20% vol Range of temperature: 20–60 °C	ND–Fe_3_O_4_ hybrid nanofluids are stable enough to be used as a created Classical models do not accurately predict thermal conductivity and viscosity Increase in concentration of nanoparticles increases both thermal conductivity and viscosity Increase in temperature increases thermal conductivity Increase in temperature decreases viscosity
Wanatasanappan et al. [74] (2020)	Nanoparticle Characteristics: Hybrid made up of Al_2_O_3_-8 and CuO-24 nm Base liquid: Water and ethylene glycol Surfactant: LAS Range of concentration: 1% vol Range of temperature: 30–70 °C	Highest thermal conductivity for hybrid nanofluid made with 60% Al_2_O_3_–40% CuO Thermal conductivity increases as temperature increases Lowest viscosity was measured for nanofluid with 20%-Al_2_O_3_–80% CuO

**Table A2:** Summary of existing theoretical modeling investigations.

Reference	Theoretical Expression	Remarks & Assumptions
Sundar et al. [4] (2013)	$k_{nf}=k_{bf}{\left(1+10.5\phi \right)}^{.1051}$	EMT based model that considers variation of temperature. Based on experimental data of Fe_3_O_4_—water nanofluid. Valid from 0 < ϕ < 2% and 20 °C < T < 60 °C
Patel et al. [20] (2010)	$k_{nf}=k_{bf}(1+0.135{\left(\frac{k_{p}}{k_{bf}}\right)}^{0.273}\phi^{0.467}{\left(\frac{T}{20}\right)}^{0.547}(\frac{100}{d_{p}}{)}^{0.234})$	Took linear regression analysis over large sets of experimental data. Including Al_2_O_3_ and CuO nanofluids. Considers thermal conductivity as function of temperature.
Vajiha & Das [85] (2009)	$k_{nf}=[\frac{k_{p}+2k_{bf}-2\phi \left(k_{bf}-k_{p}\right)}{k_{p}+2k_{bf}+\phi \left(k_{bf}-k_{p}\right)}]k_{bf}+[5\times 10^{4}\zeta \phi \rho_{bf}c_{bf}\sqrt{\frac{k_{b}T}{\rho_{p}d_{p}}}f\left(T,\phi ,\;etc.\right)]k_{bf}$	$\mathrm{Utilized}\;\mathrm{same}\;\mathrm{equation}\;\mathrm{Koo}\;\mathrm{and}\;\mathrm{Kleinstreur}\;\mathrm{proposed}\;\mathrm{with}\;\mathrm{modified}\;f\left(T,\phi ,\;etc.\right)$ and correlation Considers temperature as a variable for modifying thermal conductivity.
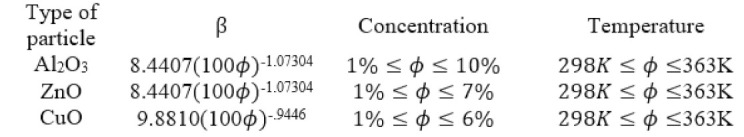
Leong et al. [79] (2006)	$k_{nf}=\frac{\left(k_{p}-k_{nl}\right)\varphi k_{nl}[2\left(\beta_{nl}{)}^{3}-\beta^{3}+1\right]+\left(k_{p}+2k_{nl}\right)\beta_{nl}{}^{3}[\phi \beta^{3}\left(k_{nl}-k_{bf}\right)+k_{bf}}{\beta_{nl}{}^{3}\left(k_{p}+2k_{nl}\right)-\left(k_{p}-k_{nl}\right)\phi \left(\beta_{nl}{}^{3}+\beta^{3}-1\right)}$	Proposed nanolayer is major mechanism in thermal conductivity enhancement. $\mathrm{Assumption}\;\mathrm{that}\;k_{nl}=2k_{bf}$ commonly used.
Xie et al. [78] (2005)	$\begin{array}{c} k_{nf}-k_{bf}=\left(3\Theta \phi +\frac{3\Theta^{2}\phi^{2}}{1-\Theta \phi }\right)k_{bf}\\ \Theta =\frac{\left(\frac{k_{nl}-k_{bf}}{k_{nl}+2k_{bf}}\right)[\left(1+\frac{\delta_{nl}}{r_{p}}{)}^{3}-\left[\frac{\left(k_{p}-k_{nl}\right)\left(k_{bf}+2k_{nl}\right)}{\left(k_{p}+2k_{nl}\right)\left(k_{bf}-k_{nl}\right)}\right]\right]}{{\left(1+\frac{\delta_{nl}}{r_{p}}\right)}^{3}+2\left[\left(\frac{k_{nl}-k_{bf}}{k_{nl}+2k_{bf}}\right)\left(\frac{k_{p}-k_{nl}}{k_{p}-2k_{nl}}\right)\right]} \end{array}$	Impact of nanolayer considered through the nanolayer conductivity and size.
Prasher et al. [83] (2005)	knf=[1+AReMPr0.333ϕkp1+2Bi+2km+2ϕ(kp1−Bi−km)kp1+2Bi+2km−ϕ(kp1−Bi−km)]kbf	Considers Brownian Motion induced convection. A and M are experimentally determined constants.
Chon et al. [22] (2005)	$\begin{array}{c} k_{nf}=(1+64.7\phi^{0.746}{\left(\frac{d_{bf}}{d_{p}}\right)}^{0.369}\left(\frac{k_{np}}{k_{bf}}{)}^{0.746}Pr^{0.9955}Re^{1.2321}\right)k_{bf}\\ Re=\frac{\rho_{bf}k_{b}T}{3\pi \mu_{bf}{}^{2}l_{bf}}\;\mathrm{and}\;Pr=\frac{c_{pbf}\mu_{bf}}{k_{bf}} \end{array}$	Utilized Buckingham—Pi theorem analyzing large sets of Al_2_O_3_—water nanofluids. $\mathrm{Molecular}\;\mathrm{diameter}\;\mathrm{of}\;\mathrm{water}\;d_{bf}$ = 0.384 nm used. $\mathrm{Mean}\;\mathrm{free}\;\mathrm{path}\;\mathrm{assumed}\;\mathrm{to}\;\mathrm{be}\;l_{bf}$ = 0.17 nm
Koo & Kleinstreur [84] (2004)	$k_{nf}=[\frac{k_{p}+2k_{bf}-2\phi \left(k_{bf}-k_{p}\right)}{k_{p}+2k_{bf}+\phi \left(k_{bf}-k_{p}\right)}]k_{b}+[5\times 10^{4}\zeta \phi \rho_{bf}c_{bf}\sqrt{\frac{k_{b}T}{\rho_{p}d_{p}}}f\left(T,\phi \right)]k_{bf}$	Indicated that Brownian motion produces micro mixing, making it dominant in thermal conductivity enhancement. The effective thermal conductivity is the sum of the thermal conductivity of static dilute suspension and thermal conductivity due to Brownian motion.
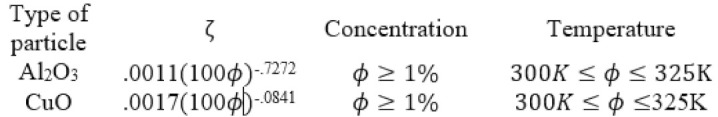
Yu & Choi [81] (2003)	$k_{nf}=\frac{k_{p}+2k_{bf}+2\left(k_{p}-k_{bf}\right){\left(1-\gamma \right)}^{3}\phi }{k_{p}+2k_{bf}-\left(k_{p}-k_{bf}\right){\left(1+\gamma \right)}^{3}\phi }k_{bf}$	Analyses the mechanism of the thermal conductivity of a nanofluid Through temperature increase, the particle surface energy decrease would reduce nanoparticle agglomeration, improving Brownian motion in the process.
Wasp et al. [77] (1977)	$k_{nf}=k_{bf}\frac{k_{p}+2k_{bf}-2\phi \left(k_{bf}-k_{p}\right)}{k_{p}+2k_{bf}+\phi \left(k_{bf}{-}_{p}\right)}$	EMT based model. Static and homogenous suspension of particles.
Tinga et al. [80] (1973)	$\frac{k_{nf}}{k_{bf}}=(1+\frac{3\phi \left[\left(\beta^{3}-1\right)\left(2k_{nl}+k_{p}\right)\left(k_{nl}-k_{bf}\right)-\left(k_{nl}-k_{p}\right)\left(2k_{nl}+k_{bf}\right)\right]}{\left(2k_{bf}+k_{nl}\right)\left(2k_{nl}+k_{p}\right)-\frac{2}{\beta^{3}-1}\left(k_{nl}-k_{bf}\right)\left(k_{nl}-k_{p}\right)-3\phi k_{nl}\left(k_{p}-k_{bf}\right)}$	Simplified model used in order to calculate thermal conductivity instead of dielectric constant. Assuming solid particle water as interfacial layer and air as host medium.
Hamilton & Crosser [76] (1962)	$k_{nf}=k_{bf}\left[\frac{k_{p}+\left(n-1\right)k_{bf}-\left(n-1\right)\left(k_{bf}-k_{p}\right)\phi }{k_{p}+\left(n-1\right)k_{bf}+\left(k_{bf}-k_{p}\right)\phi }\right]$	Based on static and homogenous EMT theory Considered particle shape due to agglomeration where n = 3 for spheres and n = 6 for cylinders.
Maxwell [75] (1904)	$k_{nf}=\frac{k_{p}+2k_{bf}+2\phi (k_{p}-k_{bf)}}{k_{p}+2k_{bf}-\phi (k_{p}-k_{bf)}}k_{bf}$	Valid for spherical particles under low concentration < 2% Based on static and homogenous dispersion of particles within base fluid.
Corcione [86] (2011)	$\begin{array}{c} k_{nf}=k_{bf}(1+4.4Re^{0.4}Pr^{0.66}{\left(\frac{T}{T_{fr}}\right)}^{10}{\left(\frac{k_{p}}{k_{bf}}\right)}^{0.03}\phi^{0.66}\\ Re=\frac{2\rho_{bf}k_{bf}T}{\pi \mu_{bf}{}^{2}d_{p}} \end{array}$	Took data from over 13 different sources of CuO, Al_2_O_3_ and TiO_2_ particles. With water and ethylene glycol base fluids. Line of best fit on large data set.
Godson et al. [7] (2010)	$k_{nf}=k_{bf}\left(0.9692\phi +0.9508\right)$	Linear regression taken over Ag—water experimental data
Khanafer & Vafai [98] (2011)	$\mu_{nf}=-0.4491+\frac{28.4312}{T}+0.574\phi -0.1634\phi^{2}+\frac{23.053\phi^{2}}{T^{2}}+0.0132\phi^{3}-\frac{2354.735\phi }{T^{3}}+\frac{23.498\phi^{2}}{d_{p}{}^{2}}-\frac{3.0185\phi^{3}}{d_{p}{}^{2}}$	Modelled using data from over 5 different sources. Valid for particles ranging in size from 13–131 nm Valid for concentrations of 1%– 9% and temperature between 20–70 °C.
Abu-Nada [102] (2011)	$\mu_{nf}=-0.155-\frac{19.582}{T}+0.794\phi +\frac{2094.47}{T^{2}}-0.192\phi^{2}-8.11\frac{\phi }{T}-\frac{27463.863}{T^{3}}+1.6044\frac{\phi^{2}}{T}+2.175\frac{\phi }{T^{2}}$	Modeled based on two-dimensional regression analysis of Nguyen’s [12] data of Al_2_O_3_ -water nanofluids.
Godson et al. [7] (2010)	$\mu_{nf}=\mu_{bf}\left(1.005+0.497\phi -0.1149\phi^{2}\right)$	Based on linear regression analysis of experimental Ag–water nanofluid.
Rea [97] (2008)	$\begin{array}{c} \mu_{nf}=\mu_{bf}e^{\left(4.91\phi /0.2092-\phi \right)}\\ \;\;\;{\mathrm{Al}}_{2}O_{2}\\ \mu_{nf}=\mu_{bf}\left(1+46.801\phi +550.82\phi^{2}\right)\\ \;\;\;\mathrm{Zn}2O- \end{array}$	Developed using regression analysis. Models for Al_2_O_3_—water and ZnO—water nanofluids.
Nguyen et al. [12] (2007)	$\begin{array}{c} \mu_{nf}=\mu_{bf}\left(0.904e^{0.148\phi }\right)\\ \;\;36\;\mathrm{nm}\;{\mathrm{Al}}_{2}O_{2}\\ \mu_{nf}=\mu_{bf}(1+0.025\phi -0.015\phi^{2}\\ \;\;47\;\mathrm{nm}\;{\mathrm{Al}}_{2}O_{2}\\ \mu_{nf}=\mu_{bf}\left(1.475-0.319\phi +0.051\phi^{2}+0.009\phi^{3}\right)\\ \;\;29\;\mathrm{nm}\;\mathrm{Cu}O \end{array}$	Developed through best fit of experimental data of 36 nm, 47 nm Al_2_O_3_ and 29 nm CuO particles in water-based fluid.
Maiga et al. [96] (2004)	$\mu_{nf}=\mu_{bf}\left(1+7.3\phi -123\phi^{2}\right)$	Model based on regression analysis based on experimental data of TiO_2_ nanofluids.
Lundgren [103] (1972)	$\mu_{nf}=\mu_{bf}\left(1+2.5\phi +\frac{25}{4}\phi^{2}+\phi^{3}\right)$	Took Einstein’s model and applied Taylor Series extension.
Brinkman [94] (1952)	$\mu_{nf}=\mu_{bf}(\frac{1}{{\left(1-\phi \right)}^{2.5}})$	Expanded Einstein’s research to increase validity for moderate concentrations.
Einstein [93] (1905)	$\mu_{nf}=\left(1+2.5\phi \right)\mu_{bf}$	Early best fit model based on experimental data Valid for low concentrations of <0.02%.

## References

[1] Arulprakasajothi M., Dineshbabu M., Jothishanmugam C., Saikrishnan V. Convective Heat Transfer Characteristics of Nanofluids. Proceedings of the Frontiers in Automobile and Mechanical Engineering.

[2] Choi U.S., Eastman J.A. Enhancing Thermal Conductivity of Fluids with Nanoparticles. Proceedings of the International Mechanical Engineering Congress and Exhibition.

[3] Yeganeh M., Shahtahmasebi N., Kompany A., Goharshadi E., Youssefi A., Šiller L. (2010). Volume fraction and temperature variations of the effective thermal conductivity of nanodiamond fluids in deionized water. Int. J. Heat Mass Transf..

[4] Sundar L.S., Singh M.K., Sousa A.C. (2013). Investigation of thermal conductivity and viscosity of Fe_3_O_4_ nanofluid for heat transfer applications. Int. Commun. Heat Mass Transf..

[5] Gao D., Bai M., Hu C., Lv J., Wang C., Zhang X. (2020). Investigating control of convective heat transfer and flow resistance of Fe_3_O_4_/deionized water nanofluid in magnetic field in laminar flow. Nanotechnology.

[6] Afrand M., Toghraie D., Sina N. (2016). Experimental study on thermal conductivity of water-based Fe_3_O_4_ nanofluid: Development of a new correlation and modeled by artificial neural network. Int. Commun. Heat Mass Transf..

[7] Godson L., Raja B., Lal D.M., Wongwises S. (2010). Experimental Investigation on the Thermal Conductivity and Viscosity of Silver-Deionized Water Nanofluid. Exp. Heat Transf..

[8] Li C.H., Peterson G.P. (2006). Experimental investigation of temperature and volume fraction variations on the effective thermal conductivity of nanoparticle suspensions (nanofluids). J. Appl. Phys..

[9] Pryazhnikov M., Minakov A., Rudyak V., Guzei D. (2017). Thermal conductivity measurements of nanofluids. Int. J. Heat Mass Transf..

[10] Xie H., Yu W., Chen W. (2010). MgO nanofluids: Higher thermal conductivity and lower viscosity among ethylene glycol-based nanofluids containing oxide nanoparticles. J. Exp. Nanosci..

[11] Yu W., Xie H., Chen L., Li Y. (2009). Investigation of thermal conductivity and viscosity of ethylene glycol based ZnO nanofluid. Thermochim. Acta.

[12] Nguyen C., Desgranges F., Roy G., Galanis N., Maré T., Boucher S., Mintsa H.A. (2007). Temperature and particle-size dependent viscosity data for water-based nanofluids—Hysteresis phenomenon. Int. J. Heat Fluid Flow.

[13] Yiamsawas T., Dalkilic A.S., Mahian O., Wongwises S. (2013). Measurement and Correlation of the Viscosity of Water-Based Al_2_O_3_ and TiO_2_ Nanofluids in High Temperatures and Comparisons with Literature Reports. J. Dispers. Sci. Technol..

[14] Kole M., Dey T. (2010). Viscosity of alumina nanoparticles dispersed in car engine coolant. Exp. Therm. Fluid Sci..

[15] Malekzadeh A., Pouranfard A.R., Hatami N., Banari A.K., Rahimi M.R. (2016). Experimental Investigations on the Viscosity of Magnetic Nanofluids under the Influence of Temperature, Volume Fractions of Nanoparticles and External Magnetic Field. J. Appl. Fluid Mech..

[16] Yu W., Xie H., Li Y., Chen L. (2011). Experimental investigation on thermal conductivity and viscosity of aluminum nitride nanofluid. Particuology.

[17] Sundar L.S., Hortiguela M.J., Singh M.K., Sousa A.C. (2016). Thermal conductivity and viscosity of water based nanodiamond (ND) nanofluids: An experimental study. Int. Commun. Heat Mass Transf..

[18] Pastoriza-Gallego M., Lugo L., Legido J., Piñeiro M.M. (2011). Thermal conductivity and viscosity measurements of ethylene glycol-based Al_2_O_3_ nanofluids. Nanoscale Res. Lett..

[19] Sundar L.S., Ramana E.V., Singh M.K., Sousa A.C. (2014). Thermal conductivity and viscosity of stabilized ethylene glycol and water mixture Al_2_O_3_ nanofluids for heat transfer applications: An experimental study. Int. Commun. Heat Mass Transf..

[20] Patel H.E., Sundararajan T., Das S.K. (2009). An experimental investigation into the thermal conductivity enhancement in oxide and metallic nanofluids. J. Nanopart. Res..

[21] Gangadevi R., Vinayagam B.K. (2018). Experimental determination of thermal conductivity and viscosity of different nanofluids and its effect on a hybrid solar collector. J. Therm. Anal. Calorim..

[22] Chon C.H., Kihm K.D., Lee S.P., Choi S.U.S. (2005). Empirical correlation finding the role of temperature and particle size for nanofluid (Al_2_O_3_) thermal conductivity enhancement. Appl. Phys. Lett..

[23] Pastoriza-Gallego M.J., Casanova C., Páramo R., Barbés B., Legido J.L., Piñeiro M.M. (2009). A study on stability and thermophysical properties (density and viscosity) of Al_2_O_3_ in water nanofluid. J. Appl. Phys..

[24] Kwek D., Crivoi A., Duan F. (2010). Effects of Temperature and Particle Size on the Thermal Property Measurements of Al_2_O_3_−Water Nanofluids. J. Chem. Eng. Data.

[25] Beck M.P., Yuan Y., Warrier P., Teja A.S. (2008). The effect of particle size on the thermal conductivity of alumina nanofluids. J. Nanopart. Res..

[26] Rudyak V.Y., Minakov A.V. (2018). Thermophysical properties of nanofluids. Eur. Phys. J. E.

[27] Ceotto D., Rudyak V.Y. (2016). Phenomenological formula for thermal conductivity coefficient of water-based nanofluids. Colloid J..

[28] Kim S.H., Choi S.R., Kim D. (2008). Thermal Conductivity of Metal-Oxide Nanofluids: Particle Size Dependence and Effect of Laser Irradiation. J. Heat Transf..

[29] Chopkar M., Sudarshan S., Das P., Manna I. (2008). Effect of Particle Size on Thermal Conductivity of Nanofluid. Metall. Mater. Trans. A.

[30] Teng T.-P., Hung Y.-H., Teng T.-C., Mo H.-E., Hsu H.-G. (2010). The effect of alumina/water nanofluid particle size on thermal conductivity. Appl. Therm. Eng..

[31] Murshed S., Leong K., Yang C. (2008). Investigations of thermal conductivity and viscosity of nanofluids. Int. J. Therm. Sci..

[32] Esfe M.H., Karimipour A., Yan W.-M., Akbari M., Safaei M.R., Dahari M. (2015). Experimental study on thermal conductivity of ethylene glycol based nanofluids containing Al_2_O_3_ nanoparticles. Int. J. Heat Mass Transf..

[33] Omrani A., Esmaeilzadeh E., Jafari M., Behzadmehr A. (2019). Effects of multi walled carbon nanotubes shape and size on thermal conductivity and viscosity of nanofluids. Diam. Relat. Mater..

[34] Turgut A., Tavman I., Chirtoc M., Schuchmann H.P., Sauter C., Tavman S. (2009). Thermal Conductivity and Viscosity Measurements of Water-Based TiO2 Nanofluids. Int. J. Thermophys..

[35] Jia-Fei Z., Zhong-Yang L., Ming-Jiang N., Ke-Fa C. (2009). Dependence of Nanofluid Viscosity on Particle Size and pH Value. Chin. Phys. Lett..

[36] Timofevva E.V., Smith D.S., Yu W., France D.M., Singh D., Routbort J.L. (2010). Particle size and interfacial effects on thermos-physical and heat transfer characteristics of water-based α-SiC nanofluids. Nanotechnology.

[37] Abdul Hamid K., Azmi W., Mamat R., Usri N., Najafi G. (2015). Investigation of Al_2_O_3_ Nanofluid Viscosity for Different Water/EG Mixture Based. Energy Procedia.

[38] Bidgoli M.R., Kolahchi R., Karimi M.S. (2016). An experimental study and new correlations of viscosity of ethylene glycol-water based nanofluid at various temperatures and different solid concentrations. Struct. Eng. Mech..

[39] Vandrangi S.K., Hassan S., Sharma K.V., Akilu S., Emani S., Nabipour N. (2020). Effect of base fluids on thermo-physical properties of SiO_2_ nanofluids and development of new correlations. Math. Methods Appl. Sci..

[40] Okonkwo E.C., Wole-Osho I., Kavaz D., Abid M., Al-Ansari T. (2020). Thermodynamic evaluation and optimization of a flat plate collector operating with alumina and iron mono and hybrid nanofluids. Sustain. Energy Technol. Assess..

[41] Wang X.-J., Zhu D.-S., Yang S. (2009). Investigation of pH and SDBS on enhancement of thermal conductivity in nanofluids. Chem. Phys. Lett..

[42] Li X., Zhu D., Wang X., Wang N., Gao J., Li H. (2008). Thermal conductivity enhancement dependent pH and chemical surfactant for Cu-H_2_O nanofluids. Thermochim. Acta.

[43] Khairul M., Shah K., Doroodchi E., Azizian R., Moghtaderi B. (2016). Effects of surfactant on stability and thermo-physical properties of metal oxide nanofluids. Int. J. Heat Mass Transf..

[44] Das P.K., Mallik A.K., Ganguly R., Santra A.K. (2018). Stability and thermophysical measurements of TiO_2_ (anatase) nanofluids with different surfactants. J. Mol. Liq..

[45] Freitas S.S., Silveira V., Jabardo J.M.S., Arce A.C. (2020). MWCNT and COOH–MWCNT aqueous nanofluids: Thermophysical and rheological characterization. J. Braz. Soc. Mech. Sci. Eng..

[46] Das P.K., Islam N., Santra A.K., Ganguly R. (2017). Experimental investigation of thermophysical properties of Al_2_O_3_–water nanofluid: Role of surfactants. J. Mol. Liq..

[47] Das S.K., Putra N., Thiesen P., Roetzel W. (2003). Temperature Dependence of Thermal Conductivity Enhancement for Nanofluids. J. Heat Transf..

[48] Esfe M.H., Afrand M., Yan W.-M., Akbari M. (2015). Applicability of artificial neural network and nonlinear regression to predict thermal conductivity modeling of Al_2_O_3_–water nanofluids using experimental data. Int. Commun. Heat Mass Transf..

[49] Krishnakumar T.S., Viswanath S.P., Varghese S.M., Prakash M.J. (2018). Experimental studies on thermal and rheological properties of Al_2_O_3_–ethylene glycol nanofluid. Int. J. Refrig..

[50] Maheshwary P., Handa C., Nemade K. (2017). A comprehensive study of effect of concentration, particle size and particle shape on thermal conductivity of titania/water based nanofluid. Appl. Therm. Eng..

[51] Shima P.D., Philip J., Raj B. (2010). Synthesis of Aqueous and Nonaqueous Iron Oxide Nanofluids and Study of Temperature Dependence on Thermal Conductivity and Viscosity. J. Phys. Chem. C.

[52] Esfe M.H., Saedodin S., Asadi A., Karimipour A. (2015). Thermal conductivity and viscosity of Mg (OH)_2_-ethylene glycol nanofluids. J. Therm. Anal. Calorim..

[53] Sundar L.S., Singh M.K., Sousa A.C. (2013). Thermal conductivity of ethylene glycol and water mixture based Fe_3_O_4_ nanofluid. Int. Commun. Heat Mass Transf..

[54] Chiam H., Azmi W., Usri N., Mamat R., Adam N. (2017). Thermal conductivity and viscosity of Al_2_O_3_ nanofluids for different based ratio of water and ethylene glycol mixture. Exp. Therm. Fluid Sci..

[55] Li X., Zou C., Qi A. (2016). Experimental study on the thermo-physical properties of car engine coolant (water/ethylene glycol mixture type) based SiC nanofluids. Int. Commun. Heat Mass Transf..

[56] Urmi W., Rahman M., Hamzah W. (2020). An experimental investigation on the thermophysical properties of 40% ethylene glycol based TiO_2_-Al_2_O_3_ hybrid nanofluids. Int. Commun. Heat Mass Transf..

[57] Sundar L.S., Ramana E.V., Singh M., Sousa A.D. (2012). Viscosity of low volume concentrations of magnetic Fe_3_O_4_ nanoparticles dispersed in ethylene glycol and water mixture. Chem. Phys. Lett..

[58] Wang F., Han L., Zhang Z., Fang X., Shi J., Ma W. (2012). Surfactant-free ionic liquid-based nanofluids with remarkable thermal conductivity enhancement at very low loading of graphene. Nanoscale Res. Lett..

[59] Al-Waeli A.H., Chaichan M.T., Sopian K., Kazem H.A. (2019). Influence of the base fluid on the thermo-physical properties of PV/T nanofluids with surfactant. Case Stud. Therm. Eng..

[60] Sundar L.S., Ramana E.V., Singh M.K., Gracio J., Sousa A.C.M. (2014). Preparation, Thermal and Rheological Properties of Propylene Glycol and Water Mixture Based Fe_3_O_4_ Nanofluids. J. Nanofluids.

[61] Kumar V., Sahoo R.R. (2019). Viscosity and thermal conductivity comparative study for hybrid nanofluid in binary base fluids. Heat Transf. Asian Res..

[62] Timofeeva E.V., Yu W., France D.M., Singh D., Routbort J.L. (2011). Base fluid and temperature effects on the heat transfer characteristics of SiC in ethylene glycol/H2O and H2O nanofluids. J. Appl. Phys..

[63] Esfe M.H., Saedodin S., Akbari M., Karimipour A., Afrand M., Wongwises S., Dahari M. (2015). Experimental investigation and development of new correlations for thermal conductivity of CuO/EG–water nanofluid. Int. Commun. Heat Mass Transf..

[64] Namburu P.K., Kulkarni D.P., Misra D., Das D.K. (2007). Viscosity of Copper Oxide Nanoparticles Dispersed in Ethylene Glycol and Water Mixture. Exp. Therm. Fluid Sci..

[65] Main K., Eberl B., Mcdaniel D., Tikadar A., Paul T.C., Khan J.A. Nanoparticles Shape Effect on Viscosity and Thermal Conductivity of Ionic Liquids Based Nanofluids. Proceedings of the 5th Thermal and Fluids Engineering Conference (TFEC).

[66] Zhu D., Wang L., Yu W., Xie H. (2018). Intriguingly high thermal conductivity increment for CuO nanowires contained nanofluids with low viscosity. Sci. Rep..

[67] Timofeeva E.V., Routbort J.L., Singh D. (2009). Particle shape effects on thermophysical properties of alumina nanofluids. J. Appl. Phys..

[68] Xing M., Yu J., Wang R. (2015). Experimental study on the thermal conductivity enhancement of water based nanofluids using different types of carbon nanotubes. Int. J. Heat Mass Transf..

[69] Singh K., Kumar S. (2020). An Experimental Study on Characterization of CuO/Water Nanofluid. Int. Res. J. Eng. Technol..

[70] Sundar L.S., Sharma K., Singh M.K., Sousa A. (2017). Hybrid nanofluids preparation, thermal properties, heat transfer and friction factor—A review. Renew. Sustain. Energy Rev..

[71] Sundar L.S., Singh M.K., Sousa A. (2014). Enhanced heat transfer and friction factor of MWCNT–Fe_3_O_4_/water hybrid nanofluids. Int. Commun. Heat Mass Transf..

[72] Lyu Z., Asadi A., Alarifi I.M., Ali V., Foong L.K. (2020). Thermal and Fluid Dynamics Performance of MWCNT-Water Nanofluid Based on Thermophysical Properties: An Experimental and Theoretical Study. Sci. Rep..

[73] Sundar L.S., Ramana E.V., Graça M., Singh M.K., Sousa A.C. (2016). Nanodiamond-Fe_3_O_4_ nanofluids: Preparation and measurement of viscosity, electrical and thermal conductivities. Int. Commun. Heat Mass Transf..

[74] Wanatasanappan V.V., Abdullah M., Gunnasegaran P. (2020). Thermophysical properties of Al_2_O_3_-CuO hybrid nanofluid at different nanoparticle mixture ratio: An experimental approach. J. Mol. Liq..

[75] Maxwell J.C. (1891). Electricity and Magnetism. A Treatise on Electricity Magnetism.

[76] Hamilton R.L., Crosser O.K. (1962). Thermal Conductivity of Heterogeneous Two-Component Systems. Ind. Eng. Chem. Fundam..

[77] Wasp F.J., Kenny J.P., Gandhi R.L. (1977). Solid-Liquid Slurry Pipeline Transportation.

[78] Xie H., Fujii M., Zhang X. (2005). Effect of interfacial nanolayer on the effective thermal conductivity of nanoparticle-fluid mixture. Int. J. Heat Mass Transf..

[79] Leong K., Yang C., Murshed S. (2006). A model for the thermal conductivity of nanofluids—The effect of interfacial layer. J. Nanopart. Res..

[80] Tinga W.R., Voss W.A.G., Blossey D.F. (1973). Generalized approach to multiphase dielectric mixture theory. J. Appl. Phys..

[81] Yu W., Choi S. (2003). The Role of Interfacial Layers in the Enhanced Thermal Conductivity of Nanofluids: A Renovated Maxwell Model. J. Nanopart. Res..

[82] Hosseini S.M., Moghadassi A.R., Henneke D.E. (2010). A new dimensionless group model for determining the viscosity of nanofluids. J. Therm. Anal. Calorim..

[83] Prasher R., Bhattacharya P., Phelan P.E. (2005). Brownian-Motion-Based Convective-Conductive Model for the Effective Thermal Conductivity of Nanofluids. J. Heat Transf..

[84] Koo J., Kleinstreuer C.A. (2005). A New Thermal Conductivity Model for Nanofluids. J. Nanopart. Res..

[85] Vajiha R.S., Das D.K. (2009). Experimental Determination of Thermal Conductivity of Three Nanofluids and Development of New Correlations. ASME J. Heat Mass Transf..

[86] Corcione M. (2011). Empirical correlating equations for predicting the effective thermal conductivity and dynamic viscosity of nanofluids. Energy Convers. Manag..

[87] Eastman J.A., Phillpot S.R., Choi S.U.S., Keblinski P. (2004). Thermal Transport in Nanofluids. Annu. Rev. Mater. Res..

[88] Lee S., Choi S.U.-S., Li S., Eastman J.A. (1999). Measuring Thermal Conductivity of Fluids Containing Oxide Nanoparticles. J. Heat Transf..

[89] Murshed S., Leong K., Yang C. (2008). Thermophysical and electrokinetic properties of nanofluids—A critical review. Appl. Therm. Eng..

[90] Shima P.D., Philip J., Baldev R. (2009). Role of Microconvection Induced by Brownian Motion of Nanoparticles in the Enhanced Thermal Conductivity of Stable Nanofluids. Appl. Phys. Lett..

[91] Yu-Hua L., Wei Q., Jian-Chao F. (2008). Temperature Dependence of Thermal Conductivity of Nanofluids. Chin. Phys. Lett..

[92] Xu J., Yu B., Zou M., Xu P. (2006). A New Model for Heat Conduction of Nanofluids Based on Fractal Distributions of Nanoparticles. J. Appl. Phys..

[93] Einstein A. (1905). Investigations on the Theory of the Brownian Movement.

[94] Brinkman H.C. (1952). The Viscosity of Concentrated Suspensions and Solutions. J. Chem. Phys..

[95] Wang X., Xu X., Choi S.U.S. (1999). Thermal Conductivity of Nanoparticle-Fluid Mixture. J. Thermophys. Heat Transf..

[96] Maïga B.S., Nguyen C.T., Galanis N., Roy G. (2004). Heat transfer behaviours of nanofluids in a uniformly heated tube. Superlattices Microstruct..

[97] Rea U., Mckrell T., Hu L.-W., Buongiorno J. (2004). Laminar convective heat transfer and viscous pressure loss of alumina–water and zirconia–water nanofluids. Int. J. Heat Mass Transf..

[98] Khanafer K., Vafai K. (2011). A critical synthesis of thermophysical characteristics of nanofluids. Int. J. Heat Mass Transf..

[99] Pak B.C., Cho Y.I. (1998). Hydrodynamic and Heat Transfer Study of Dispersed Fluids with Submicron Metallic Oxide Particles. Exp. Heat Transf..

[100] Putra N., Roetzel W., Das S.K. (2003). Natural Convection of Nanofluids. Heat Mass Transf..

[101] Anoop K.B., Kabelac S., Sundararajan T., Das S.K. (2009). Rheological and flow characteristics of nanofluids: Influence of electroviscous effects and particle agglomeration. J. Appl. Phys..

[102] Abu-Nada E. (2011). Rayleigh-Bénard convection in nanofluids: Effect of temperature dependent properties. Int. J. Therm. Sci..

[103] Lundgren T.S. (1972). Slow flow through stationary random beds and suspensions of spheres. J. Fluid Mech..

